# Assessment of fecal DNA extraction protocols for metagenomic studies

**DOI:** 10.1093/gigascience/giaa071

**Published:** 2020-07-13

**Authors:** Fangming Yang, Jihua Sun, Huainian Luo, Huahui Ren, Hongcheng Zhou, Yuxiang Lin, Mo Han, Bing Chen, Hailong Liao, Susanne Brix, Junhua Li, Huanming Yang, Karsten Kristiansen, Huanzi Zhong

**Affiliations:** School of Future Technology, University of Chinese Academy of Sciences, Beijing 101408, China; BGI-Shenzhen, Bei Shan Industrial Area, Yantian, Shenzhen 518083, China; BGI Europe A/S, COBIS, 2200 Copenhagen, Denmark; Laboratory of Genomics and Molecular Biomedicine, Department of Biology, University of Copenhagen, 2100 Copenhagen, Denmark; BGI Europe A/S, COBIS, 2200 Copenhagen, Denmark; BGI-Shenzhen, Bei Shan Industrial Area, Yantian, Shenzhen 518083, China; Laboratory of Genomics and Molecular Biomedicine, Department of Biology, University of Copenhagen, 2100 Copenhagen, Denmark; China National Genebank, Jinsha Road, Dapeng District, Shenzhen 518120, China; BGI-Shenzhen, Bei Shan Industrial Area, Yantian, Shenzhen 518083, China; BGI-Shenzhen, Bei Shan Industrial Area, Yantian, Shenzhen 518083, China; Laboratory of Genomics and Molecular Biomedicine, Department of Biology, University of Copenhagen, 2100 Copenhagen, Denmark; BGI-Shenzhen, Bei Shan Industrial Area, Yantian, Shenzhen 518083, China; China National Genebank, Jinsha Road, Dapeng District, Shenzhen 518120, China; Department of Biotechnology and Biomedicine, Technical University of Denmark, 2800 Kgs. Lyngby, Denmark; BGI-Shenzhen, Bei Shan Industrial Area, Yantian, Shenzhen 518083, China; School of Biology and Biological Engineering, South China University of Technology, Guangzhou 510006, China; BGI-Shenzhen, Bei Shan Industrial Area, Yantian, Shenzhen 518083, China; James D. Watson Institute of Genome Sciences, Hangzhou 310058, China; BGI-Shenzhen, Bei Shan Industrial Area, Yantian, Shenzhen 518083, China; Laboratory of Genomics and Molecular Biomedicine, Department of Biology, University of Copenhagen, 2100 Copenhagen, Denmark; BGI-Shenzhen, Bei Shan Industrial Area, Yantian, Shenzhen 518083, China; Laboratory of Genomics and Molecular Biomedicine, Department of Biology, University of Copenhagen, 2100 Copenhagen, Denmark

**Keywords:** DNA extraction, gut microbiota, human fecal sample, shotgun metagenomic sequencing

## Abstract

**Background:**

Shotgun metagenomic sequencing has improved our understanding of the human gut microbiota. Various DNA extraction methods have been compared to find protocols that robustly and most accurately reflect the original microbial community structures. However, these recommendations can be further refined by considering the time and cost demands in dealing with samples from very large human cohorts. Additionally, fungal DNA extraction performance has so far been little investigated.

**Results:**

We compared 6 DNA extraction protocols, MagPure Fast Stool DNA KF Kit B, Macherey Nagel™ NucleoSpin™®Soil kit, Zymo Research Quick-DNA™ Fecal/Soil Microbe kit, MOBIO DNeasy PowerSoil kit, the manual non-commercial protocol MetaHIT, and the recently published protocol Q using 1 microbial mock community (MMC) (containing 8 bacterial and 2 fungal strains) and fecal samples. All samples were manually extracted and subjected to shotgun metagenomics sequencing. Extracting DNA revealed high reproducibility within all 6 protocols, but microbial extraction efficiencies varied. The MMC results demonstrated that bead size was a determining factor for fungal and bacterial DNA yields. In human fecal samples, the MagPure bacterial extraction performed as well as the standardized protocol Q but was faster and more cost-effective. Extraction using the PowerSoil protocol resulted in a significantly higher ratio of gram-negative to gram-positive bacteria than other protocols, which might contribute to reported gut microbial differences between healthy adults.

**Conclusions:**

We emphasize the importance of bead size selection for bacterial and fungal DNA extraction. More importantly, the performance of the novel protocol MP matched that of the recommended standardized protocol Q but consumed less time, was more cost-effective, and is recommended for further large-scale human gut metagenomic studies.

## Background

The adult human gut harbors highly complex and diverse microbial communities, including bacteria, archaea, fungi, viruses, and protozoa [1]. The composition of the gut bacterial community has been demonstrated to exhibit associations with multiple human diseases, including type 2 diabetes [2–4], obesity [5–7], and colorectal cancer [8, 9]. However, many studies have shown how different experimental processing pipelines affect the results [10, 11] and how especially DNA extraction affects the quantitative characterization of bacterial components [11–13], emphasizing the need for a standardized and robust protocol for profiling of the gut microbiota to enable true comparison between studies.

During the past 2 decades, PCR-based amplicon sequencing, a flexible and cost-effective method to determine microbial composition, has greatly improved our understanding of the human microbiome. However, considering the known effects of PCR conditions on amplification biases such as primers, specific hypervariable regions, and annealing temperature [14, 15], amplicon sequencing is insufficient for accurately evaluating the quantitative performance of bacterial DNA extraction protocols. In comparison, shotgun metagenomic sequencing is a more accurate tool for analyzing the microbiota. A recent shotgun sequencing–based benchmark study has comprehensively investigated bacterial extraction performances of 21 fecal DNA extraction protocols, including widely used extraction kits and non–kit-based protocols [11]. By evaluation of DNA quantity and quality, community diversity, and extraction efficiency of gram-positive and gram-negative bacteria, this study has proposed protocol Q, a manual protocol based on a modified version of Qiagen's QIAamp® DNA Stool Mini Kit, as a standard protocol for human fecal bacterial DNA extraction [11]. However, there is still room for improvement to establish less labor-intensive and more cost-effective alternative standardized protocols, especially for large-scale gut microbiome studies. Additionally, assessment of fungal DNA extraction performance in fecal samples, the often neglected important players in the overall gut microbiome [16–18], is still scarce.

In the present study, we assessed the DNA extraction performance of 6 protocols on a microbial mock community (MMC) comprising 8 bacterial and 2 yeast strains, and on fecal samples from 6 healthy human individuals, using protocol Q as a reference method. On the basis of extractions of the MMC, we established a positive correlation between the bead size and extraction efficiency of yeast DNA, providing information for the selection of appropriate DNA extraction protocols for fungal-related studies. On the basis of extractions from human fecal samples, we found that a time- and cost-effective kit-based protocol, protocol MP, exhibited bacterial DNA extraction performance similar to protocol Q regarding DNA yield, bacterial community diversity, and relative abundances of gram-positive and gram-negative bacteria.

## Data Description

**Figure 1:**
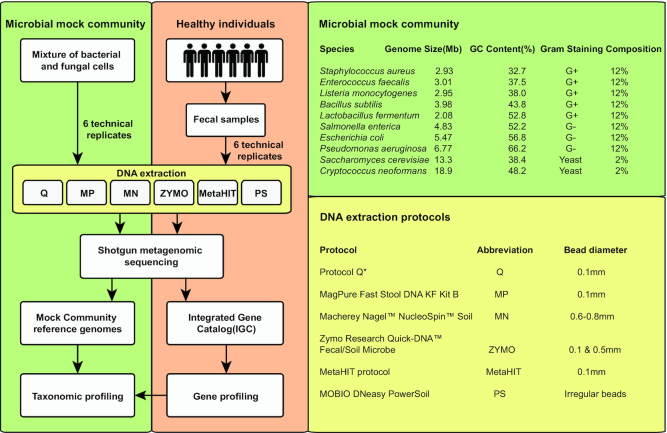
Schematic workflow of study design. Comparison of 6 DNA extraction protocols using a microbial mock community (MMC) and fecal samples from 6 individuals via shotgun metagenomics sequencing. Tables present strain information of the MMC (8 bacterial and 2 yeast strains) (top right) and 6 DNA extraction protocols (bottom right). G+: gram-positive; G−: gram negative.

DNA extraction protocols (Supplementary Table S1) on 2 types of biological samples, including a 10-species MMC and human fecal samples from 6 healthy individuals (Fig. 1, Methods). The MMC (Catalog No. D6300), containing cells of 8 species of bacteria (each making up 12%) and 2 yeast strains (each contributing 2%), was purchased from Zymo Research (Fig. 1). Among the 6 protocols, 3 kit-based methods including MagPure Fast Stool DNA KF Kit B (MP, Guangzhou, China), Macherey Nagel™ NucleoSpin™®Soil kit (MN, Düren, Germany), and Zymo Research Quick-DNA™ Fecal/Soil Microbe kit (ZYMO, Freiburg, Germany) had not been thoroughly evaluated in the previous studies [19, 20]. In addition, we also included 3 protocols used in the benchmark study [11], including protocol Q (Hilden Germany), MOBIO DNeasy PowerSoil kit (PS, Hilden, Germany), and a non–kit-based manual protocol adopted by MetaHIT (Metagenomics of the Human Intestinal Tract Consortium) for evaluating the reproducibility of the DNA extraction protocols. DNA of all samples was manually extracted in the laboratory of BGI Europe A/S, COBIS, Copenhagen, Denmark. All 6 protocols used in this study included a step of mechanical cell disruption by bead beating (see full standard operating procedure [SOP] of each protocol in Supplementary File F1). For each protocol, 6 technical replicates were generated from the MMC and each human fecal sample. In total, 233 qualified DNA samples (36 MMC extractions and 197 human fecal DNA extractions) were subjected to shotgun sequencing and further quantitative analyses (Supplementary Table S2).

## Analyses

### Assessment of processing time and DNA yield

Among the 6 protocols, 4 kit-based protocols (MP, MN, ZYMO, and PS) were much more effective in relation to DNA processing time than the 2 manual protocols (Q and MetaHIT) (40–100 minutes vs 156–380 minutes per extraction) (Supplementary Table S1). We next compared DNA yields between the protocols. Using the amount of starting material as given in the Methods section, extraction of MMC yielded on average 0.77 μg DNA per sample, whereas extraction of human fecal samples on average yielded 4.31 μg DNA per sample (Supplementary Table S2). The PS kit gave significantly lower DNA yields than protocol MN and ZYMO on the MMC. The PS kit also showed significantly lower DNA yields than all other protocols on human fecal samples except for protocol ZYMO (Benjamini-Hochberg [BH]-adjusted Dunn*P* < 0.05, Supplementary Table S3, Supplementary Fig. S1), in line with previous observations [12, 21–23]. On the other hand, we found inconsistent performances of protocol Q in retrieving DNA from the MMC and human fecal samples. Protocol Q delivered significantly lower DNA yields than protocols MP, MN, and ZYMO on the MMC (BH-adjusted Dunn*P* < 0.05, Supplementary Fig. S1A) but showed similar DNA yields on human fecal samples when compared with protocols MP, MN, and ZYMO (BH-adjusted Dunn *P* > 0.05, Supplementary Fig. S1B).

### Evaluation of DNA extraction protocols on the mock community

**Figure 2:**
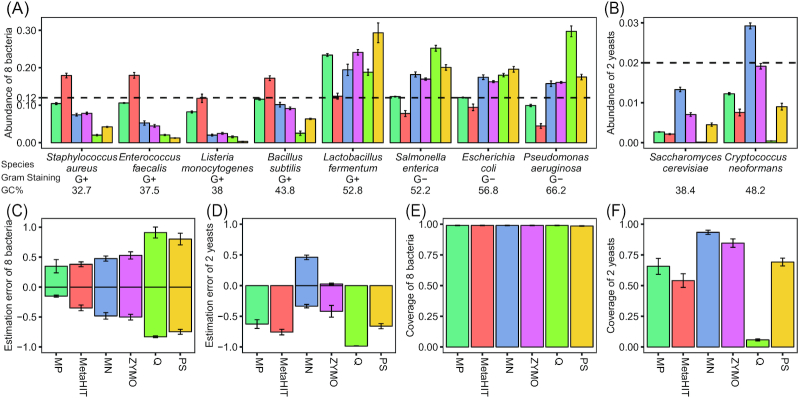
Performance of the 6 different DNA extraction protocols on an MMC. **A, B**, Bar plot showing the mean observed relative abundances of 8 bacteria (A) and 2 yeasts (B) using the 6 extraction protocols. **C, D**, Estimation error (EE) of 8 bacteria (C) and 2 yeasts (D) in all technical replicates for each protocol. **E, F**, Genome coverage of 8 bacteria (E) and 2 yeasts (F) using the 6 extraction protocols. Genome coverage is calculated as the proportion of the genome reference covered by ≥1 read. The error bars show standard error of the mean in all panels.

We first estimated the relative abundances of the bacterial and yeast strains obtained using the 6 protocols and based on the reference genomes of the MMC (see details in the Methods section). Focusing on the 8 bacterial strains, we found that except for the protocol MetaHIT, 6 replicates from each of the remaining 5 protocols tended to consistently underestimate gram-positive bacteria including *Staphylococcus aureus, Enterococcus faecalis, Listeria monocytogenes*, and *Bacillus subtilis* but overestimated all 3 gram-negative members (*Salmonella enterica, Escherichia coli*, and *Pseudomonas aeruginosa*) (Fig. 2A). By combining results from all 8 bacterial strains, we observed that the protocol MP showed a relatively higher mean accuracy in bacterial abundance estimations than the other protocols (mean estimation error [MEE]: 0.22, Fig. 2C), followed by protocol MetaHIT and protocol MN (MEE < 0.5, Fig. 2C). All 6 protocols provided almost complete genome recovery of the 8 bacterial strains (genome coverage, mean ± SD: 98.90% ± 1.5%, Fig. 2E). However, the recovery of the 2 yeast genomes (*Saccharomyces cerevisiae* and *Cryptococcus neoformans*) was much lower than that of the bacterial genomes and varied considerably between protocols (genome coverage, mean ± SD: 62.11% ± 31.52%, Fig. 2F). Of note, 2 protocols using relatively large beads (MN with 0.6–0.8 mm diameter beads and ZYMO with 0.5 mm diameter beads) ensured higher relative abundances and genome coverages of the 2 yeast strains than protocols with 0.1 mm diameter beads (MP, MetaHIT, and Q) (Fig. 2B, D, and F). Additionally, we also observed very low intra-protocol variabilities in performance on microbial abundance estimation (Fig. 2A and B) and genome recovery (Fig. 2E and F), indicating high reproducibility of each protocol.

Asking whether there was a robust positive correlation between bead size and fungal DNA yield, we subsequently conducted a bead size–dependent extraction experiment. Briefly, we tested protocol MP using 3 types of bead conditions (500 μL of φ0.1 mm; 250 μL of φ0.1 mm plus 250 μL of φ0.6–0.8 mm; 500 μL of φ0.6–0.8 mm) on cell cultures of *E. coli* K-12 MG1655 (*E. coli* MG1655), *S. cerevisiae* BY4741, and a mixture of *E. coli* MG1655 and *S. cerevisiae* BY4741 (2:1, v/v), with 10 extraction replicates per condition. By quantifying and comparing DNA yields between groups (Supplementary Table S4), we found that protocol MP using beads of 0.6–0.8 mm diameter either alone or in combination with beads of 0.1 mm diameter gave significantly higher DNA yields of *S. cerevisiae* than the protocol using beads of 0.1 mm diameter (Wilcoxon rank-sum test, *P* < 0.05, Supplementary Fig. S2). By contrast, the protocol using beads of 0.1 mm diameter showed significantly higher DNA yields of *E. coli* than the protocol containing only beads of 0.6–0.8 mm diameter or the combination of these beads with beads of 0.1 mm diameter (Wilcoxon rank-sum test, *P* < 0.05, Supplementary Fig. S2), indicating the difficulty of simultaneously unbiased bacterial and fungal DNA extraction.

### Evaluation of the DNA extraction protocols on human fecal samples

**Figure 3:**
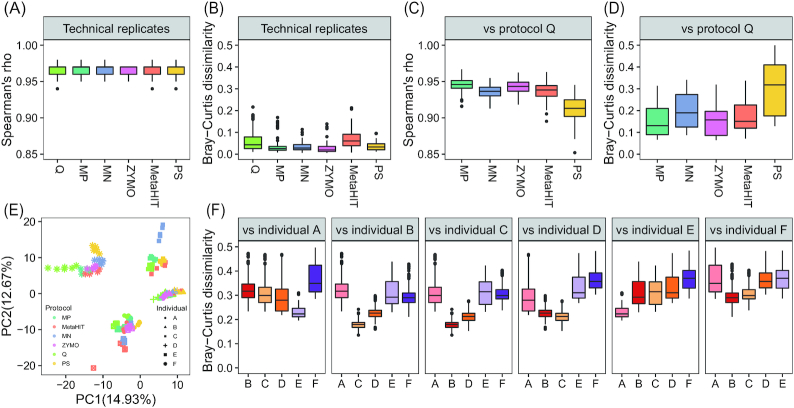
Intra- and interprotocol consistency in species quantification using human fecal samples. **A, B**, Spearman ρ (A) and Bray-Curtis dissimilarities (B) between 6 technical replicates within each protocol. **C, D**, Spearman ρ (C) and Bray-Curtis dissimilarities (D) between protocol Q and the 5 other protocols. **E**, Principal component analysis (PCA) based on species profile. Colors indicate different protocols: light green, protocol Q; green, protocol MP; blue, protocol MN; purple, protocol ZYMO; orange, protocol MetaHIT; yellow, protocol PS. Different shapes indicate DNA samples from different individuals. **F**, Box plots showing the inter-individual Bray-Curtis dissimilarities using the same protocol. Each panel indicates Bray-Curtis dissimilarities between samples from a given individual and that from others. The boxs represent the range between the first and the thired quartiles and the vertical line inside the box represent the median.

We next evaluated the intra- and interprotocol performance on human fecal samples. Spearman rank correlation analysis revealed high coefficient values between technical replicates at both gene (Supplementary Fig. S3A, averaged Spearman ρ = 0.875) and species level (Fig. 3A, averaged Spearman ρ = 0.964). Likewise, the average Bray-Curtis dissimilarities between intraprotocol replications were 0.142 at the gene level (Supplementary Fig. S3B) and 0.046 at the species level (Fig. 3B). These results suggest high intraprotocol reproducibility in the quantification of relative abundance of human gut microbial genes and species.

There were no significant differences in microbial richness between protocols at the gene and the species level (Kruskal-Wallis test, *P* > 0.05, Supplementary Fig. S4A and B, Supplementary Table S5). However, we observed significantly lower microbial diversity in samples extracted by protocol Q compared to protocols MN and ZYMO, the 2 large bead-based protocols (BH-adjusted Dunn *P* < 0.05, Supplementary Fig. S4C and D, Supplementary Table S5). Interprotocol analyses further demonstrated smaller values of Spearman rank coefficients (Supplementary Fig. S3C, Fig. 3C) and greater microbial Bray-Curtis dissimilarities (Supplementary Fig. S3D, Fig. 3D) of microbial profiles between samples extracted by the PS and protocol Q compared to those between other protocols and protocol Q. On the other hand, regardless of DNA extraction protocols, datasets from the same individual were grouped on a principal component analysis (PCA) plot (Fig. 3E) and showed greater dissimilarities between each other than between intra- or interprotocol replications (Fig. 3F). This is in agreement with the previous notion that interindividual variation exceeds the variation resulting from different protocols [ 13, 22, 24–26].

**Figure 4:**
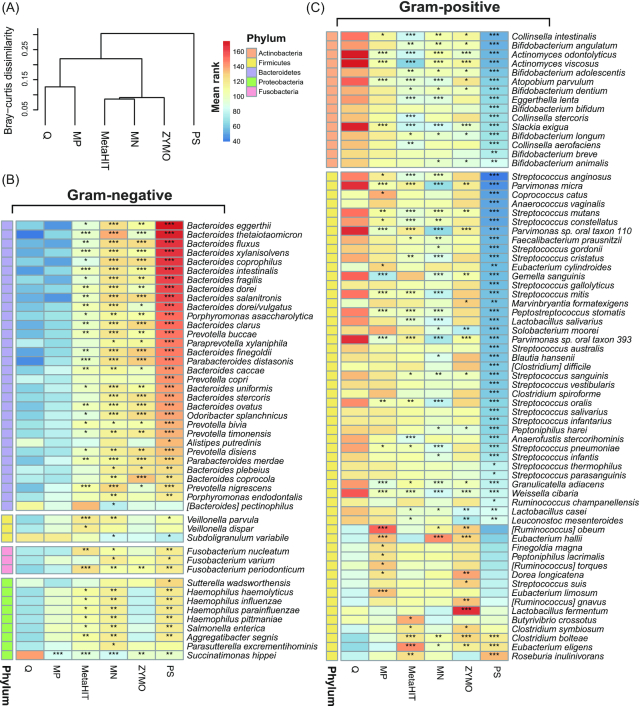
Protocol-dependent differences in the relative abundance of gut bacterial species. **A**, Clustering of samples extracted by the different protocols based on species-level Bray-Curtis dissimilarities. **B, C**, Heat map showing gram-negative (B) and gram-positive (C) species that differ significantly in abundance between protocol Q and the other protocols. Color key indicates the mean rank of relative abundance of each species between comparisons in the Kruskal-Wallis test. Pairwise comparisons using the Dunn test were followed by the Kruskal-Wallis test. *BH-adjusted Dunn *P* < 0.05; **BH-adjusted Dunn *P* < 0.01; ***BH-adjusted Dunn *P* < 0.001. The color bar indicates phylum assignment of each species: orange, Actinobacteria; yellow, Firmicutes; purple, Bacteroidetes; green, Proteobacteria; pink, Fusobacteria. A list of all species that differ significantly in abundance among the 6 protocols is presented in Supplementary Table S6.

Based on cluster analysis, we further revealed larger species compositional dissimilarities between PS-extracted samples and samples extracted using the other protocols (Fig. 4A). In addition, we found comparable species composition comparing samples extracted by protocols MP and Q, and between samples extracted using protocols MN and ZYMO, respectively (Fig. 4A). We assessed differences in the quantification performance of individual species between protocols by confining our analyses to 210 common species of ≥20% occurrence among samples (see details in the Methods section). Of note, 72.38 (152 of 210) differed significantly in relative abundance between ≥2 protocols (Kruskal-Wallis test, BH-adjusted *P* < 0.05, Supplementary Table S6). In line with the benchmark study [11], the relative abundances of multiple gram-positive species were significantly higher in Q-extracted samples than those extracted using the protocol PS, including species from the genera *Bifidobacterium, Collinsella, Streptococcus*, and *Parvimonas* (Fig. 4C, BH-adjusted Dunn*P* < 0.05). By contrast, the relative abundances of multiple gram-negative species annotated to the genera *Bacteroides, Prevotella*, and *Haemophilus* were consistently and significantly lower in Q- and MP-extracted samples compared with those extracted using the other protocols (Fig. 4B, BH-adjusted Dunn *P* < 0.05).

Furthermore, we found that PS-extracted samples exhibited significantly lower abundances of gram-positive species but higher abundances of gram-negative species than samples extracted by using the other 5 protocols (Supplementary Fig. S5, BH-adjusted Dunn*P* < 0.05). By plotting the abundance distributions of selected abundant gut species, including 6 gram-positive species (*Bifidobacterium adolescentis, Bifidobacterium longum, Faecalibacterium prausnitzii, Collinsella intestinalis, Streptococcus anginosus*, and *Streptococcus cristatus*) and 6 gram-negative species (*Alistipes putredinis, Bacteroides coprocola, Bacteroides dorei, Bacteroides dorei/vulgatus, Bacteroides ovatus*, and *Prevotella copri*), we found that species-related quantitative biases between PS and the other protocols were consistent among all individuals (Supplementary Fig. S6). We further replicated a consistent and significant enrichment of 52 species comparing metagenomic datasets of PS-extracted samples and the 3 Qiagen kit-based protocols from the benchmark study (Supplementary Fig. S7, BH-adjusted Dunn*P* < 0.05) [11].

In the current shotgun metagenomic datasets, we only detected very low levels of fungi species (0.03–2.32%) in fecal samples from individuals C and F using MetaPhlAn2 [27] (Supplementary Table S7). However, by extraction of human fecal samples, we did not observe the same clear relation between bead size and fungal DNA extraction yield as observed using the MMC, further underscoring the difficulties in choosing an extraction protocol providing a robust, accurate representation of both bacterial and fungal DNA.

### DNA extraction biases may contribute to reported country-specific signatures

**Figure 5:**
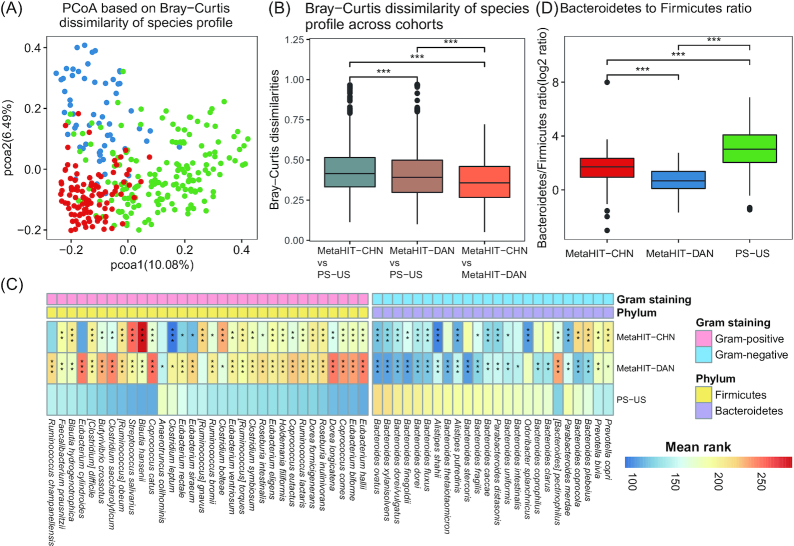
Links between country-specific gut microbial signatures and the corresponding fecal DNA extraction protocols. **A**, Principal coordinate analysis (PCoA) based on species-level Bray-Curtis dissimilarities between the 3 cohorts. Red, Chinese adults (protocol MetaHIT); blue, Danish adults (protocol MetaHIT); green, US adults (protocol PS). B, Comparison of Bray-Curtis dissimilarities at the species level between the 3 cohorts. Grey, Chinese vs US adults; brown, Danish vs US adults; orange, Chinese vs Danish adults. **C**, Heat map showing the significantly differed gram-negative species from Bacteroidetes, and gram-positive species from Firmicutes between Chinese, Danish, and US adults. Color key indicates the mean rank of relative abundance of each species between comparisons in the Kruskal-Wallis test. D, Bacteroidetes to Firmicutes ratio (B/F ratio) between Chinese, Danish, and US adults. Y-axis indicates log_2_-transformed values of the B/F ratio. For **B–-D**, pairwise comparisons using the Dunn test were performed after the Kruskal-Wallis test. *BH-adjusted Dunn *P* < 0.05; **BH-adjusted Dunn *P* < 0.01; ***BH-adjusted Dunn *P* < 0.001.

To investigate to what extent differences between the performance of DNA extraction protocols might influence reported results on country-specific gut microbial signatures, we compared available shotgun metagenomic datasets of healthy Chinese (n = 60) and Danish adults (n = 100) (protocol MetaHIT) [28] to healthy US adults (n = 167) from the Human Microbiome Project (HMP, protocol PS) [29]. Samples from the 3 countries separated clearly from each other in principal coordinate analysis (PCoA) plots (Fig. 5A). Still, we noted that species profiles of Chinese and Danish adults, whose fecal samples were extracted using the protocol MetaHIT, exhibited less Bray-Curtis dissimilarity than that observed between US adults (Fig. 5B). Furthermore, we found that PS-extracted US samples exhibited significantly higher abundances of multiple gram-negative species and lower abundances of gram-positive species than those of MetaHIT-extracted samples from both Chinese and Danish adults (BH-adjusted Dunn*P* < 0.05, Fig. 5C). Such quantitative differences may contribute to a significantly higher Bacteroidetes to Firmicutes ratio in US adults as compared to Chinese and Danish adults (Fig. 5D). More detailed comparisons of samples from different countries need to be scrutinized using identical extraction and sequencing protocol to determine to what extent these differences truly reflect country/ethnicity-dependent differences. These observations emphasize that cautions must be taken in interpreting gut microbial findings observed using different DNA extraction methods, and that standardized extraction protocols are needed for reliable comparison of samples from different ethnic groups.

## Discussion

In this study, 6 DNA extraction protocols were assessed using both MMC and human fecal samples subjected to shotgun metagenomics sequencing. Experiments using MMC revealed that protocols with smaller bead size yielded higher bacterial DNA recovery, whereas protocols with larger bead size yielded higher fungal DNA recovery. However, the latter could not be replicated using human fecal samples. Assessment of human fecal samples showed a varied extraction efficiency of gram-positive and gram-negative species between protocols, especially between the PS and the other protocols. We propose that such protocol-dependent differences might contribute to the reported gut microbial differences between cohorts from different countries and of different ethnicity. We report that protocol MP, a time- and cost-effective method, compared with the other protocols evaluated in this study, exhibited an extraction performance in characterizing and quantifying bacterial communities similar to the recently proposed standard protocol Q [11]. Therefore, we propose protocol MP as a robust and alternative standard protocol for human fecal DNA extraction in future large-scale metagenomics studies. However, we emphasize that the performance of the extraction protocols tested on fecal samples in the present study needs to be evaluated for use on other human-related samples (e.g., saliva and skin) because the microbial composition, as well as physical and chemical properties of such samples, is quite distinct from those of fecal samples. Future large-scale metagenomics projects will need to use automated DNA extraction. Thus, a limitation of this study is that the performance of the MagPure kit in relation to a robotized extraction system was not evaluated, and further efforts are required to assess the stability and consistency between manual and automated DNA extraction using the MagPure kit.

With a known species composition, MMC allowed us to investigate the DNA extraction efficiency of both bacteria and fungi. All 6 protocols in this study included a bead-beating step, the most effective mechanical lysis method [13, 24, 30–32], with different sizes and composition of beads. Regardless of technical differences between the protocols, we found that 2 protocols (MN and ZYMO) with large beads (0.5–0.8 mm) showed significantly better performance in the recovery of fungal genomes and theoretical abundances than other protocols with beads of 0.1 mm diameter. Of note, our experiments on a mock community of *E. coli* MG1655, *S. cerevisiae* BY4741, and a simple mixture of *E. coli* MG1655 and *S. cerevisiae* BY4741 showed that a large bead–based method (φ0.6–0.8 mm) secured high extraction efficiency of yeast but simultaneously sacrificed the extraction efficiency of bacteria. Therefore, extraction methods with combinations of beads of different sizes seem warranted for further studies aiming to achieve an accurate and reliable representation of microbial communities with both bacteria and fungi even though the combinations of small and large bead sizes used in the present study were unable to improve simultaneous recovery of bacterial and fungal DNA. We were unable to evaluate the fungal DNA extraction efficiency using human fecal samples owing to low levels of detection of fungal taxa from the 6 volunteers in the current shotgun metagenomic datasets. It has been demonstrated that the number of fungi in human feces is far less than that of bacteria [1, 33–36], with 10^5^–10^6^ fungal cells per gram of feces compared with 10^11^ bacterial cells per gram [36]. In addition, the genome sizes of fungi are much larger than those of bacteria. Thus, a much greater amount of sequencing data than we generated in the present study would be needed to evaluate the performance of fecal mycobiome extraction across protocols. Amplicon-based approaches (18S ribosomal RNA–based or internal transcribed spacer–based) seem still to be more cost-effective and appropriate to assess and interpret the mycobiome in human fecal samples, and such amplicon-based approaches have been successfully applied in several studies [35, 37, 38].

Another observation was the inconsistency in relation to the extraction efficiency of gram-positive and gram-negative species using MMC and human fecal samples extracted by the same protocols. For instance, except for MetaHIT, all other protocols including protocol Q underestimated the relative abundance of 4 gram-positive strains (*S. aureus, E. faecalis, L. monocytogenes*, and *B. subtilis*) and overestimated the relative abundance of the 3 gram-negative strains tested (*S. enterica, E. coli*, and *P. aeruginosa*) in the MMC samples. Likewise, the benchmark study [11] showed that regardless of whether DNA was extracted from a mock community or from a fecal sample with a spike-in mock community, protocol Q underestimated the abundances of gram-positive bacteria including *Clostridium perfringens, Clostridioides difficile*, and *Lactobacillus plantarum* and overestimated the abundances of 3 gram-negative members including *S. enterica, Prevotella melaninogenica*, and *Fusobacterium nucleatum*. By contrast, human fecal DNA samples extracted by protocol Q displayed better performance in the quantification of gram-positive species than the other protocols. In addition, the mock communities from both studies were both composed of human pathogenic bacteria or bacteria isolated from a non-human environment, which do not reflect the human gut microbial composition. Furthermore, such simple mixtures of bacteria and fungi do not contain other compounds in feces such as humic acids, polysaccharides, bile acids, and lipids, which might potentially inhibit the activity of enzymes used for subsequent PCR-based library construction and sequencing [39]. Thus, extraction performance based on MMC may not precisely and unbiasedly reflect extraction performance on human fecal samples. Finally, for both studies, quantitative performance on extracting human gut microbiome between protocols has been interpreted on the basis of relative bacterial abundances, but not absolute abundances, which we measured in the MMC. Further efforts are still needed to quantify absolute microbial abundances in fecal mock materials with a mixture of both abundant gut microbes and additional fecal compounds, and in real fecal samples to accurately assess the quantification biases of different protocols.

## Potential Implications

DNA extraction protocols affect the outcome of metagenomics studies, and standardized, validated, and cost- and time-effective protocols are needed for large-scale metagenomics projects. We compared 6 commonly used DNA extraction protocols using 1 MMC and fecal samples. Evaluation of the results based on shotgun metagenomic sequencing revealed the importance of bead sizes for bacterial and fungal DNA extraction. Microbial extraction efficiencies varied among protocols. The performance of the novel MagPure Fast Stool DNA KF Kit B matched that of the recommended standardized protocol Q but consumed less time, was more cost-effective, and is recommended for large-scale studies.

## Methods

### Sample collection and preparation

#### Microbial mock community

ZymoBIOMICS Microbial Community Standard, Catalog No. D6300 (Microbial Mock Community, MMC), was obtained from Zymo Research. The MMC contains 8 bacteria with the same abundance: *Staphylococcus aureus, Enterococcus faecalis, Listeria monocytogenes, Bacillus subtilis, Salmonella enterica, Lactobacillus fermentum, Escherichia coli*,and*Pseudomonas aeruginosa* and 2 yeast species, also with the same abundance: *Saccharomyces cerevisiae* and *Cryptococcus neoformans*. The theoretical relative abundance of each bacterial strain is 12% and that of each fungal strain 2% (Fig. 1).

#### Human fecal sample collection

Six healthy volunteers including a 4-year-old child and 5 adults (32 ± 3 years old) were recruited from BGI Europe employees or family members, Copenhagen, Denmark (see detailed information in Supplementary Table 2). All volunteers or the guardian provided informed consent to provide fecal samples for this study. Roughly 10–15 grams of stool were freshly collected by participants at home by using a 50-mL sterile conical tube, and copies of printed instructions were used to guide the adult volunteers or the child's legal guardian for self-collection of fecal samples. After collection, samples were stored at −20°C and transported to the laboratory on the second day with ice packs in 40 minutes. Then, each sample was diluted with 1–1.5 volumes (15 mL) of Tris-EDTA (10 mM Tris pH 8.0 and 1 mM EDTA, Thermo Fisher Scientific) buffer, homogenized, and divided into 36 aliquots (500 μL per aliquot). All stool aliquots were stored at −80°C before DNA extraction.

### DNA extraction, library preparation, and sequencing

All DNA extraction experiments examining the 6 different protocols were performed manually by the same technician at the BGI Europe laboratory, Copenhagen, Denmark, and the bead size experiments were performed at BGI-Shenzhen. The DNA extraction was conducted in accordance with the manufacturer's instructions or protocols provided (see full SOP of each protocol in Supplementary File F1). For both mock community and human fecal samples, 6 technical replicates were generated using each protocol. The DNA concentration was detected by Qubit® 2.0 fluorometer (Invitrogen). Considering the different starting volume used in each protocol, we normalized the DNA yield to the volume of starting material.

All 36 DNA samples from the MMC were successfully extracted by the 6 extraction protocols, and library construction and sequencing were successful for all 36 DNA samples. Six fecal samples extracted using protocol PS (individual E) and 13 fecal samples extracted using protocol ZYMO (6 of individual A, 6 of individual C, and 1 of individual F) that yielded <500 ng and failed for library preparation were removed from further processing. Library preparation and shotgun metagenomic sequencing were performed on the BGISEQ-500 platform using the paired-end 100 mode [40]. Low-quality reads and human-derived reads were filtered to generate high-quality non-human reads as described previously [40], resulting in an averaged proportion of high-quality non-human reads of 94.33% per sample (including MMC and human fecal samples, coefficient of variation [CV] = 6.63%) (Supplementary Table S2). In total, 233 shotgun metagenomic datasets from 36 MMC DNA extractions and 197 human fecal DNA extractions were generated and evaluated for the performance of the 6 protocols (Supplementary Table S2).

### Comparison of DNA extraction kits using mock communities

The 10 microbial reference genomes of the MMC are available online (see Availability of Supporting Data and Materials). To minimize the potential impacts of sequencing depth on quantitative and qualitative assessment of the composition of the MMC, we randomly downsized each sample to 20 million high-quality paired reads and aligned the reads to the reference genomes using SOAP 2.22 (m = 0, x = 1000, r = 1, l = 30, M = 4, S, *p* = 6, v = 5, S, c = 0.95).

For all protocols, the total mapping ratio, defined as the ratio of the total number of mapped reads to the total number of high-quality reads, reached 98.32% on average (CV = 0.27%). The relative abundance of each strain was calculated as the ratio of the number of mapped reads onto the reference genome to the total number of mapped reads onto all reference genomes. Genome coverage of each strain was calculated as the proportion of the genome reference covered by ≥1 read (SOAP coverage 2.7.7). For each species, the estimation error (EE) was used to represent the extraction bias, defined as

\begin{eqnarray*}
{\mathrm{EE}} = \frac{{{\mathrm{Observed\ relative\ abundance}} - {\mathrm{Theoretical\ relative\ abundance}}}}{{{\mathrm{Theoretical\ relative\ abundance}}}}. \end{eqnarray*}

For each protocol, the MEE was proposed to represent the extraction accuracy, i.e.,

\begin{equation*} {\mathrm{MEE}} = \overline {\left| {EE} \right|}, \end{equation*}

where $\overline {| {EE} |} \ $ is the mean absolute value of the EE for all species in all technical replicates for each protocol.

A second-round DNA extraction experiment was performed to validate the positive correlation between bead sizes of DNA extraction protocols and DNA yield of yeast. Three types of bead conditions were assessed, including (i) 500 μL of φ0.1 mm beads, (ii) 250 μL of φ0.1 mm beads mixed with 250 μL of φ0.6–0.8 mm beads, and (iii) 500 μL of φ0.6–0.8 mm beads based on the MagPure Fast Stool DNA KF Kit B (MP). Three simple cell cultures were prepared for extraction testing, each in a volume of 1 mL, including (i) only *E. coli* K-12 MG1655 (*E. coli* MG1655), (ii)*S. cerevisiae* BY4741, and (iii) a combination of two-thirds volume of *E. coli* MG1655 and one-third volume of *S. cerevisiae* BY4741. Extractions were carried out with 10 technical replicates for each type of bead conditions on each kind of sample. In total, DNA yields of 90 extractions were measured and compared between the different bead conditions (Supplementary Table S4).

### Comparison of DNA extraction kits using human fecal samples

#### Taxonomic profiling of shotgun metagenomic sequencing data from human fecal samples

High-quality and non-human reads were first aligned to the Integrated Gene Catalog (IGC) (SOAP 2.22 m = 0, x = 1000, r = 2, l = 30, M = 4, S, *p* = 6, v = 5, S, c = 0.95) [28]. On average, 79.67% (CV = 2.03%) high-quality reads could be aligned to ≥1 gene from IGC. Uniquely mapped reads were then downsized to 20 million pairs for each sample to calculate gene relative abundance. The relative abundance of each species was computed on the basis of the sum of relative abundance of genes annotated to the given species as described previously [28]. A total of 477 bacterial and archaeal species were identified in this study. We then confined our species-based comparison analyses to common species, which was defined as species with >100 annotated genes in all samples and with an occurrence in >20% of the samples.

#### Taxonomic profiling using MetaPhlAn2

The IGC-based taxonomic annotation pipeline was previously developed on the basis of 3,449 bacterial and archaeal taxa [28], lacking the information of fungal taxa. Aiming to evaluate fungal quantitative performance in human fecal samples, we next performed taxonomic annotation and quantification using MetaPhlAn2 (version 2.7.0) [27] and generated microbial profiles including bacteria, eukaryotes, archaea, and viruses for all 197 human fecal samples.

#### α diversity and richness analyses

To estimate the richness and evenness of the microbial community in fecal samples, we calculated α diversity using the Shannon index at the gene and species level using the function diversity in the R package vegan (R version 3.4.1). Richness was defined as the number of observed genes or species in each sample.

### Available shotgun metagenomic datasets from published studies

To validate the reliability of the observed difference between gram-positive and gram-negative species between different protocols, we selected 28 human fecal sample datasets from a published benchmark study [11], including 8 datasets from DNA extracted by protocol PS and 20 datasets from DNA extracted by 3 Qiagen QIAamp® DNA Stool Mini Kit-based protocols (8 datasets from Q-6, 8 datasets from Q-9, and 4 datasets from Q-15) (Supplementary Table S8).

To investigate whether there are potential links between country-specific gut microbial signatures and the corresponding fecal DNA extraction protocols, shotgun metagenomic datasets of fecal DNA were retrieved from 60 healthy Chinese adults and 100 healthy Danish adults extracted using protocol MetaHIT [28] and from 167 healthy US adults (HMP) extracted using protocol PS [29]. Detailed information of these 327 metagenomic datasets is provided in Supplementary Table S9. IGC-based taxonomic assignment and quantification of all published datasets were performed as described above but without downsizing of the 327 country-specific signature comparison datasets.

## Statistical Analyses

### Correlation analysis

The Spearman correlation coefficient was calculated using function cor.test from the R package stats to estimate a rank-based measure of association.

### Bray-Curtis dissimilarity and PCoA

Bray-Curtis dissimilarities at the gene and species level were calculated using the vegdist (method = “bray”) function from the R package vegan.PCoA was performed to visualize the Bray-Curtis dissimilarities using the R package ade.

### Kruskal-Wallis test

To determine which species differed significantly in abundance between samples extracted by different extraction protocols, and samples from different countries, the Kruskal-Wallis test was performed using the function kruskal.test from the R package stats. The Benjamini-Hochberg (BH) method was applied for adjustment of *P*-values of the Kruskal-Wallis tests, using the p.adjust (method = “BH”) function from R package stats. A BH-adjusted Kruskal-Wallis *P*-value < 0.05 was considered as statistically significant between multiple groups (≥3). Pairwise tests for multiple comparisons were followed by the Kruskal-Wallis test, using the function posthoc.kruskal.dunn.test from the R package PMCMR. Dunn*P*-values were calculated for each pairwise comparison and a BH-adjusted Dunn*P-*value < 0.05 was considered as statistically significant between each 2 groups.

## Availability of Supporting Data and Materials

Metagenomic sequence data of the 36 MMC samples and 197 fecal DNA samples have been deposited in the CNSA [41] of China National GeneBank Database (CNGBdb) with accession No. CNP0000497 and in the European Nucleotide Archive (ENA) under BioProject ERP121404. Twenty-eight published shotgun metagenomic sequencing datasets from the benchmark study are available at the ENA under BioProject ERP016524. Published shotgun metagenomic sequencing datasets of 60 Chinese and 100 Danish adults are available at ENA with BioProject ID ERP004605 and ERP003612, respectively. Published shotgun metagenomic sequencing datasets of 167 US adults are available at the SRA [42] and the Database of Genotypes and Phenotypes (dbGaP [43]) under the 2 studies SRP002163 (BioProject PRJNA48479) and SRP056641 (BioProject PRJNA275349). The 10 microbial reference genomes of the MMC are available at https://s3.amazonaws.com/zymo-files/BioPool/ZymoBIOMICS.STD.genomes.ZR160406.zip. Other data further supporting this work are openly available in the *GigaScience* database, GigaDB [44].

## Additional Files

**Supplementary TableS1**. Key parameters of the 6 DNA extraction protocols used in this study.

**Supplementary Table S2**. Summary of metagenomic sequencing data of the 36 microbial mock community (MMC) samples and 197 human fecal samples.

**Supplementary Table S3**. Statistical differences of DNA yields of MMC and human fecal samples between protocols.

**Supplementary Table S4**. DNA yields of bacteria and yeast using different bead conditions.

**Supplementary Table S5**. Statistical differences of Shannon index and richness at the gene and species level between DNA extraction protocols.

**Supplementary Table S6**. List of 152 common species that differ significantly in abundance between the 6 DNA extraction protocols.

**Supplementary Table S7**. Summary of taxonomic assignments of the 197 human fecal samples using MetaPhlAn2.

**Supplementary Table S8**. List of retrieved samples from a published benchmark study for comparison of protocol PS and 3 Q-based protocols.

**Supplementary Table S9**. List of retrieved metagenomic samples from published studies for country-specific signatures comparison.

**Supplementary File F1**. Full SOP of 6 DNA extraction proto-cols.

**SupplementaryFigure S1**. Comparison of DNA yields from MMC (a) and human fecal samples (b) between protocols. Thirty-six available MMC datasets (6 datasets per protocol) and 107 available human fecal metagenomic datasets (individual B, n = 36; individual D, n = 36; and individual F, n = 35) were used. Comparisons between protocol Q vs the other protocols, and protocol PS vs the other protocols are shown in this figure. *Adjusted Dunn*P <* 0.05; **adjusted Dunn*P* < 0.01; ***adjusted Dunn*P* < 0.001. Detailed results of the 6 protocols are provided in Supplementary Table S3.

**Supplementary Figure S2**. Comparison of bacterial and fungal DNA yields using different bead conditions. DNA extraction performance was assessed using 3 cell cultures including only *Escherichia coli* K-12 MG1655 (*E. coli* MG1655) (Bacteria, left), *Saccharomyces cerevisiae* BY4741 (*S. cerevisiae* BY4741) (Yeast, right) and a combination of two-thirds volume of *E. coli* MG1655 and one-third volume of *S. cerevisiae* BY4741 (Mixture, middle). Color bars indicate bead conditions based on MagPure Fast Stool DNA KF Kit B (MP); green, 500 μL of φ0.1 mm beads; purple, 250 μL of φ0.1 mm beads mixed with 250 μL of φ0.6–0.8 mm beads; orange, 500 μL of φ0.6–0.8 mm beads. For each experimental group, extractions were carried out with 10 technical replicates. Wilcoxon rank-sum test, *P* < 0.05. *P < 0.05; **P < 0.01; ***P < 0.001.

**Supplementary Figure S3**. Gene-level intra- and interprotocol consistency using human fecal samples for extraction and analysis. Spearman ρ (a) and Bray-Curtis dissimilarities (b) between 6 technical replicates within each protocol. Spearman ρ (c) and Bray-Curtis dissimilarities (d) between protocol Q and the 5 other protocols.

**Supplementary Figure S4**. Comparison of gene count (a), species count (b), gene-based Shannon diversity (c), and species-based Shannon diversity (d) between protocol Q and the other 5 protocols. One hundred seven available human fecal metagenomic datasets (individual B, n = 36; individual D, n = 36; and individual F, n = 35) were used. *Adjusted Dunn *P* < 0.05; **adjusted Dunn *P* < 0.01; ***adjusted Dunn *P* < 0.001. NS, not significant. Detailed results between 6 protocols are provided in Supplementary Table S5.

**Supplementary Figure S5**. Heat map showing gram-negative (a) and gram-positive (b) species that differ significantly in abundance between protocol PS and other protocols. Color key indicates the mean rank of relative abundance of each species between comparisons in the Kruskal-Wallis test. Dunn post hoc tests were followed by the Kruskal-Wallis test and Dunn *P*-values were adjusted by the BH method. *Adjusted Dunn *P* < 0.05; **adjusted Dunn *P* < 0.01; ***adjusted Dunn *P* < 0.001. The color bar indicates phylum assignment of each species: orange, Actinobacteria; yellow, Firmicutes; purple, Bacteroidetes; green, Proteobacteria; pink, Fusobacteria. A list of all species that differ significantly in abundance between the 6 protocols is provided in Supplementary Table S6.

**Supplementary Figure S6**. Relative abundance distributions of representative gut bacterial species at the individual level. (a) Gram-positive species, (b) gram-negative species. Each point indicates the relative abundance of a given species from an individual sample. Light green, protocol Q; green, protocol MP; blue, protocol MN; purple, protocol ZYMO; orange, protocol MetaHIT; yellow, protocol PS. X-axis indicates 6 individuals (A–F); Y-axis indicates log_2_-transformed relative abundance of a given species.

**Supplementary Figure S7**. Heat map showing species that differ significantly in abundance between protocol PS and 3 Q-based protocols. Comparisons were performed on 28 published metagenomic datasets (protocol PS, n = 8; protocol Q-6, n = 8; protocol Q-9, n = 8; protocol Q-15, n = 4) from a published benchmark study [11]. Color key indicates the mean rank of relative abundance of each species between comparisons in the Kruskal-Wallis test. Dunn post hoc tests were followed by the Kruskal-Wallis test. *Adjusted Dunn *P* < 0.05; **adjusted Dunn *P* < 0.01; ***adjusted Dunn *P* < 0.001. The color bar indicates phylum assignment of each species: orange, Actinobacteria; yellow, Firmicutes; purple, Bacteroidetes; green, Proteobacteria; pink, Fusobacteria and the gram staining characteristics of species: red, gram-positive; blue, gram-negative. Blue indicates species with the same enrichment direction between protocol PS and Q in the present study.

giaa071_GIGA-D-20-00011_Original_Submission

giaa071_GIGA-D-20-00011_Revision_1

giaa071_Response_to_Reviewer_Comments_Original_Submission

giaa071_Reviewer_1_Report_Original_Submission Emily Vogtmann -- 2/19/2020 Reviewed

giaa071_Reviewer_2_Report_Original_Submission Young-Do Nam -- 2/27/2020 Reviewed

giaa071_Reviewer_2_Report_Revision_1 Young-Do Nam -- 4/7/2020 Reviewed

giaa071_Supplemental_Files

## Abbreviations

BH: Benjamini-Hochberg; CV: coefficient of variation; EE: estimation error; GC: guanine-cytosine; HMP: Human Microbiome Project; IGC: Integrated Gene Catalog; Mb: megabase pairs; MMC: microbial mock community; MetaHIT: Metagenomics of the Human Intestinal Tract Consortium; MP: MagPure Fast Stool DNA KF Kit B; MN: Macherey Nagel™ NucleoSpin™®Soil kit; SOP: standard operating procedure; MEE: mean estimation error; PCA: principal component analysis; PCoA: principal coordinate analysis; PS: MOBIO DNeasy PowerSoil kit; SRA: Sequence Read Archive; ZYMO: Zymo Research Quick-DNA™ Fecal/Soil Microbe kit.

## Competing Interests

The authors declare that they have no competing interests.

## Funding

This research was funded by the National Science and Technology Major Project of China (No. 2017ZX10303406) and Shenzhen Municipal Government of China(No. JCYJ20170817145809215).

## Authors' Contributions

H. Zhong, J.S., and K.K. designed the study. J.S. and H. Luo performed fecal sample collection and DNA extraction experiments on ZYMO mock community and human fecal samples. F.Y., H. Zhou, M.H., B.C., and H. Liao designed and performed independent DNA extraction experiments with varied bead conditions on mock communities with *E. coli* and/or *S. cerevisiae*. H. Zhong and J.S. designed and supervised the data analyses. F.Y., H.R., and Y.L. performed the metagenomic data analyses. F.Y. and J.S. wrote the first version of the manuscript. H. Zhong, J.L., S.B., and K.K. revised the manuscript. All authors participated in discussions and contributed to shape the manuscript. All authors read and approved the final manuscript.

## Ethics Approval and Consent to Participate

The study was approved by the institutional review board of BGI under ethical document BGI-R039–1. Participants in this study provided written informed consent before sample collection.
