# Supplementary material for: Assessment of fecal DNA extraction protocols for metagenomic studies
Source: Gigascience. 2020 Jul 13;9(7):giaa071. doi: 10.1093/gigascience/giaa071 (PMC7355182; doi:10.1093/gigascience/giaa071)
Supplement: giaa071_GIGA-D-20-00011_Revision_1 [file giaa071_giga-d-20-00011_revision_1.pdf]

GigaScience

Assessment of fecal DNA extraction protocols for metagenomic studies

--Manuscript Draft--

Manuscript Number:	GIGA-D-20-00011R1
Full Title:	Assessment of fecal DNA extraction protocols for metagenomic studies
Article Type:	Research
Funding Information:	National Science and Technology Major Project of China (No:2017ZX10303406) — Dr. Junhua Li<br>Shenzhen Municipal Government of China (No. JCYJ20170817145809215) — Mrs Huanzi Zhong
Abstract:	Background<br><br>Shotgun metagenomic sequencing has greatly improved our understanding of the human gut microbiota. Efforts have been made to evaluate the performance of various DNA extraction methods to recommend protocols that robustly and most accurately reflect the original microbial community structures. However, so far recommended standardized bacterial DNA extraction protocols still may be improved, especially considering future demands in relation to time and cost dealing with samples from very large cohorts. Additionally, fungal DNA extraction performance has so far been little investigated.<br><br>Results<br><br>Here, we compared six DNA extraction protocols, MagPure Fast Stool DNA KF Kit B (MP), Macherey Nagel™ NucleoSpin™®Soil kit (MN), Zymo Research Quick-DNA™ Fecal/Soil Microbe kit (ZYMO), MOBIO DNeasy PowerSoil kit (PS), the manual protocol MetaHIT, and the recently published protocol Q using one microbial mock community (MMC) (containing eight bacterial and two fungal strains) and fecal samples. Evaluation of results using the MMC demonstrated that bead size was a determining factor for fungal and bacterial DNA yields. Evaluation of human fecal samples revealed that the bacterial extraction performance of protocol MP matched that of the standardized protocol Q, but consumed less time and was more cost-effective. Extraction using the protocol PS resulted in a significantly higher ratio of gram-negative to gram-positive bacteria than other protocols, which might potentially contribute to reported gut microbial differences between healthy US, Chinese and Danish adults, where fecal DNA samples were extracted using protocols PS and MetaHIT.<br><br>Conclusions<br><br>We reveal the importance of bead size selection for bacterial and fungal DNA extraction. More importantly, we demonstrate that the novel, time- and cost- effective protocol MP in terms of consistency and performance was equal to standardized protocol Q, and we recommend the use of MP for further large-scale metagenomic studies.
Corresponding Author:	Fangming Yang<br><br>CHINA
Corresponding Author Secondary Information:	
Corresponding Author's Institution:	
Corresponding Author's Secondary Institution:	
First Author:	Fangming Yang
First Author Secondary Information:	

Powered by Editorial Manager® and ProduXion Manager® from Aries Systems Corporation

**Order of Authors:**	Fangming Yang
	Jihua Sun
	Huainian Luo
	Huahui Ren
	Hongcheng Zhou
	Yuxiang Lin
	Mo Han
	Bing Chen
	Hailong Liao
	Susanne Brix
	Junhua Li
	Huanming Yang
	Karsten Kristiansen
	Huanzi Zhong
**Order of Authors Secondary Information:**	

Response to Reviewers:

We appreciate the reviewers' thorough advice and comments. By the changes made in the revised manuscript and the responses provided below, we hope that we have adequately addressed the reviewers' concerns.

Reviewer reports:
Reviewer #1:
In this study, six DNA extraction protocols were compared for whole genome shotgun metagenomic sequencing using one mock community and fecal samples. From the extraction of the mock community and individual cell cultures, larger beads were found to be associated with greater fungal extraction efficiency, but lower efficiency for bacteria. The MagPure kit had the highest mean accuracy in bacterial abundance from the mock community samples, but the five protocols other than the MetaHIT proposal tended to underestimate gram-positive bacteria and overestimate gram-negative bacteria. From the DNA extraction from human fecal samples, the PowerSoil kit appeared to have higher relative abundances of multiple gram-positive species and lower abundances of gram-negative species compared to all of the other protocols. However, individuals tended to cluster together regardless of DNA extraction method in a PCoA plot. The difference between PowerSoil extracted samples and the MetaHIT protocol were highlighted using previous data of health Chinese, Danish, and US adults where the US samples extracted using the PowerSoil had increased gram-negative and decreased gram-positive levels compared to the Chinese and Danish samples extracted using the MetaHIT protocol.

This is a valuable contribution to the literature as DNA extraction protocols need to be evaluated in order to establish recommended protocols for large-scale studies. Some specific comments on the manuscript are below.

Response:
We thank the reviewer for his/her positive comments on our manuscript. However, we need to clarify that one of our findings was that the use of the PowerSoil kit appeared to produce lower relative abundances of multiple gram-positive species and higher abundances of gram-negative species compared to all of the other protocols.

Major comments:

* Line 87: I commend the authors for including the full protocol details they used in this study. However, while looking through the listed protocols, it appears that automation was not used for any of the extraction protocols. One justification of this study was that standardized protocols are needed for large-scale population studies (line 65), but large-scale population studies would never use fully manual extractions. This should

be mentioned as a limitation of the study.

Response: We thank reviewer 1 for this constructive comment.

We have modified the manuscript (in abstract and methods) to clearly describe that all six extraction protocols were manually processed by the same technician in the same lab.

The main aim of our study was to evaluate DNA extraction performance between five protocols and the recently proposed standardized protocol Q [1] for human fecal samples. As demonstrated by Costea et al., the consistent performance of Q (modified based on the commercial QIAamp® DNA Stool Mini Kit) in efficiently extracting DNA of gram-positive bacteria from human fecal samples was extensively assessed and proven by 21 worldwide laboratories. However, the proposed protocol Q has many processing steps (~156min /per extraction, Supplementary Table 1), and it can hardly be achieved automatically. On the other hand, except for the protocol Q and protocol MetaHIT, the rest four protocols we tested were all commercial kits and can be adopted in semi-automated DNA extraction systems.

In this study, we demonstrate that the MagPure kit showed highly consistent extraction performance for human fecal samples comparable to protocol Q, but required fewer processing steps and less time (~45min /per extraction, Supplementary Table 1). We agree with the reviewer's comment that large-scale studies would never use fully manual extractions, and we believe that our results provide useful information for further developing and improving an automated, standardized fecal DNA extraction protocol/platform. We have also modified the discussion section in our manuscript to mention the limitation that the performance of the MagPure kit on an automatic extraction system was not evaluated in this study, and further efforts are required to assess the stability and consistency between manual and automated DNA extraction using this kit.

* Line 248: It would be helpful to discuss the limitations overall of this study. For example, these results may not extend to other sample types.

Response:
We thank reviewer 1 for this constructive comment.
We agree that our main conclusions concerning the performance of different DNA extraction protocols were drawn from human fecal samples, and these findings could not be directly generalized to other sample types (such as non-human environmental samples and those with high host DNA load and very low biomass) without further detailed studies on different sample types.
This limitation has been discussed in the revised version of the manuscript.

* Line 255: Were any blank samples included for each extraction method? Was sufficient DNA recovered for sequencing?

Response:
We did not include any blank samples for DNA extraction in this study.
We are aware that, for amplicon-based studies and extraction studies on low-biomass samples, blank samples (negative controls) are necessary to assess and trace the sources of possible nucleic acid contamination introduced from multiple experimental procedures.

For most samples, we did extract sufficient DNA from both the mock microbial community (average 0.77μg per sample) and real human fecal samples (average 4.31μg per sample) for shotgun metagenomic sequencing (see details of the DNA yield per sample in Supplementary Table 2). Also, all metagenomic datasets generated from DNA extracts of the mock microbial community had more than 97% of the total clean reads aligned to the ten reference genomes used in the mock community (SOAP 2.22, m=0, x=1000, r=1, l=30, M=4, S, p=6, v=5, S, c=0.95; see details of the reads mapping rate per sample in Supplementary Table 2), suggesting few contaminations were introduced during the extractions.

* Line 265: More details about the study participants and sample collection would be

helpful. For example, what proportion were women? What was the age range of adults? How was the fecal sample collected? How long did it take for the samples to be transported back to the laboratory?

Response: We apologize for the omissions in the Methods section.
We have added detailed information (sex and age) of the six participants in the revised Supplementary Table S2.
We have also modified the methods (in line 291-300) to clearly describe the process of collection, transport and storage of fecal samples before DNA extraction: "Six healthy volunteers including one four-year-old child and five adults (32 ± 3 years old) were recruited from BGI Europe employees or family members, Copenhagen, Denmark (See detailed information in Supplementary Table 2). All volunteers or the guardian consented to provide fecal samples for this study. About 10-15 grams of stool was freshly collected per participant at home by using a 50mL sterile conical tube, and copies of printed instructions were used to guide the adult volunteers or the child's legal guardian for self-collection of fecal samples. After collection, samples were stored at -20 °C and transported to the laboratory on the second day with ice packs in forty minutes. Then, each sample was diluted with 1~1.5 volumes (15 mL) of Tris-EDTA (TE, 10 mM Tris pH 8.0 and 1 mM EDTA,Thermo Fisher Scientific) buffer, homogenized and divided into 36 aliquots (500 µL per aliquot). All stool aliquots were stored at -80 °C before DNA extraction."

* Line 278: It would be important in the results to describe the failures - it looks like all samples from specific individuals failed for the PowerSoil and Zymo extractions. If you restrict to only individuals present in all extraction methods, are your results consistent?

Response:
We thank the reviewer for this valuable suggestion.
Yes, we are aware of the extraction failure in specific individuals, as also stated in the Methods section (in line 310-313) : "Six fecal samples extracted using protocol PS (individual E) and 13 fecal samples extracted using protocol ZYMO (six of individual A, six of individual C, and one of individual F) that yielded less than 500ng and failed for library preparation, were removed from further processing."

As all extractions on the human fecal samples by a given protocol were performed in parallel at the same time, the failure is unlikely to be caused by any laboratory procedures. However, we did not have enough fecal samples for a second-round extraction experiment on these failed samples (as a total of 36 samples per individual were used to generate six technical replicates for six different methods). On the other hand, we have successfully constructed sequencing libraries and sequenced DNA from all 36 extractions of the mock microbial community, although they had a lower microbial DNA yield as compared to the human fecal samples.

One explanation for the failure of samples from specific individuals could be that certain extraction kits (PowerSoil and Zymo) might not effectively remove complex compounds (such as humic acids, polysaccharides, bile acids and lipids, which were not contained in the mock microbial community) in fecal samples which possibly might act as PCR inhibitors to impact sequencing library construction[2].

As we show in Figure S6 (Relative abundance distributions of representative gut bacterial species at the individual level), the PowerSoil kit yielded obviously higher relative abundances of six selected representative gram-negative species (yellow) in all five individuals than the other five extraction protocols. Consistently, the MP and protocol Q had similar low relative abundances of all gram-negative species (light green and green) in all human fecal samples. These observations at the individual level were in line with the findings based on all samples (Figure 4).

To address the reviewer's concerns, we here also present the intra-individual relative abundances of 40 species in DNA samples extracted by all protocols.
In each individual, PS-extracted samples (yellow) showed consistently lower relative abundances of most gram-positive species (upper panel, Rebuttal Figure 1a) and higher relative abundances of most gram-negative species (lower panel, Rebuttal Figure 1b) than those extracted using the other protocols. Also, samples extracted by

Q and MP showed consistently lower relative abundances of most gram-negative species than PS and other protocols (Rebuttal Figure 1b). These observations were consistent in different individuals and were in line with our main reported findings (Figure 4).

Rebuttal Figure 1 Relative abundance distributions of representative gut bacterial species within each individual.
(a), gram-positive species, (b), gram-negative species. Each line indicates the relative species abundance in the DNA sample extracted using a given extraction protocol. Light green, protocol Q; green, protocol MP; blue, protocol MN; purple, protocol ZYMO; orange, protocol MetaHIT; yellow, protocol PS. X-axis indicates log2 transformed relative abundance of a given species, only top 20 abundant gram-positive (a) or gram-negative (b) species are shown (Y axis). Each panel indicates an individual (A to F).

Minor comments:
* Line 57: Shotgun metagenomics also has its own limitations so you cannot completely ignore 16S rRNA gene sequencing.

Response: We thank reviewer 1 for this constructive suggestion.
In the revised manuscript, we have stated: “During the past two decades, PCR-based amplicon sequencing, a flexible and cost-effective method to determine microbial composition, has greatly improved our understanding of human microbiome. However, considering the known effects of PCR conditions on amplification biases such as primers, specific hypervariable regions, and annealing temperature[3,4], amplicon sequencing is insufficient for accurately evaluating quantitative performance of bacterial DNA extraction protocols.”

* Line 245: Mock communities in a matrix similar to a fecal sample would be ideal since the artificial communities do not reflect potential inhibitors and other materials found in a fecal sample.

Response:

We fully agree with the reviewer that a mock community in a matrix similar to a human fecal sample would be an ideal material to evaluate the performance of fecal DNA extraction protocols. However, due to the difficulty of culturing various kinds of anaerobic gut microbes in the laboratory, there is still no available standardized, commercial mock microbial community related to human feces. Also, as we discussed above, a mixture of microbial communities could hardly mimic the complex mixture of compounds in real fecal samples, which might inhibit the activities of enzymes for PCR-based library construction.

In the revised manuscript, we have extended the limitations of the microbial mock community in line 262-266: “In addition, the mock communities from both studies were both composed of human pathogenic bacteria or bacteria isolated from a non-human environment, which do not reflect the human gut microbial composition. Furthermore, such simple mixtures of bacteria and fungi do not contain other compounds in feces such as humic acids, polysaccharides, bile acids and lipids, which might potentially inhibit the activity of enzymes used for subsequent PCR-based library construction and sequencing[2].”

Reviewer #2:
The manuscript titled "Assessment of fecal DNA extraction protocols for metagenomics studies" by Yang et al. describe the higher efficacy of MP method for the fecal DNA extraction procedure. This study also compares validity and reproducibility of six different DNA extraction method with mock and human fecal samples. As mentioned by the authors, standardized and robust DNA extraction protocol is still needed for the comparison between globally produced gut microbiome data. In addition, I agree with the necessity of new analytical methods for comprehensive and accurate understand of gut mycobiome as well. In that respect, I think your manuscript is timely necessary and important. However, there are some point might be considered in revision.

Powered by Editorial Manager® and ProduXion Manager® from Aries Systems Corporation

The most important point is that the microorganisms contained in currently used mock community are not abundant members of human gut microbiome. In the recent study by Sunagawa et al., as referred in your manuscript, the authors construct a mock community considering human gut microbial composition. While MP showed higher mean accuracy in bacterial abundance estimation than other protocol and Q showed lowest recovery of the two yeast genomes in mock sample analysis. Microbiome extracted with Q protocol still have distinct community composition compared to the other method, even with MP protocol, especially in G+ bacteria. So, I recommend the authors check where this discrepancy come from with the other mock samples or manually constructed human microbiome mock samples.

Response: We thank reviewer 2 for this constructive comment.

In the current study, we used a commercial mock community (ZymoBIOMICS Microbial Community Standard, Catalog No. D6300) containing cells of eight bacteria (each making up 12%) and two yeast strains (each contributing 2%). All these species are human pathogens or isolated from a non-human environment, facultative anaerobes (easy to be cultured), and are not high-abundant residents in the human gut.

Similarly, the benchmark study (Sunagawa et al, metioned by the reviewer should be Costea et al, 10.1038/nbt.3960) also used a mock community containing 10 bacterial species that were generally absent from the healthy gut microbiota, including Prevotella melaninogenica (G-), Clostridium perfringens (G+), Salmonella enterica (G-, also used in the current study), Clostridium difficile (G+), Lactobacillus plantarum (G+), Clostridium saccharolyticum (G+), Yersinia pseudotuberculosis (G-), Vibrio cholerae (G-), Blautia hansenii (G+) and Fusobacterium nucleatum (G-)(Costea et al, 10.1038/nbt.3960, Figure 6). Thus, both studies did not use representative and high-abundant gut microbes for the mock materials.

(Figure 6, Costea et al, 10.1038/nbt.3960)

We are aware that the assessment of mock microbial community might not fully reflect the extraction performance in real human fecal samples. Also, we did observe the inconsistency of the extraction efficiency of gram-positive bacteria between the mock community and fecal samples. Except for the MetaHIT protocol, all other five protocols underestimated the abundance of four among the five gram-positive bacteria in the mock (Staphylococcus aureus, Enterococcus faecalis, Listeria monocytogenes and Bacillus subtilis) but overestimated the abundance of gram-positive Lactobacillus fermentum (Figure 2). By contrast, four protocols (MN, ZYMP, Q and PS) overestimated the abundance of all three gram-negative bacteria (Salmonella enterica, Escherichia coli and Pseudomonas aeruginosa) (Figure 2). As reported by Costea et al (10.1038/nbt.3960, Figure 6), regardless of extracting DNA from the mock itself or from fecal sample with a spike-in mock community, protocol Q (blue) underestimated the abundance of gram-positive bacteria including C. perfringens, C. difficile and L. plantarum and overestimated the abundance of three gram-negative members including P. melaninogenica, S. enterica and F. nucleatum. My suggestion: regardless of whether DNA was extracted from a mock community or from a fecal sample with a spike-in mock community, protocol Q underestimated the abundances of gram-positive bacteria including Clostridium perfringens, C. difficile and Lactobacillus plantarum and overestimated the abundance of three gram-negative members including S. enterica, Prevotella melaninogenica and Fusobacterium nucleatum. Thus, the observations based on mock communities in the two studies were somehow consistent, suggesting overall different efficiencies of obtaining whole-genome DNA from gram-positive and gram-negative bacteria, as well as variable efficiencies between different gram-positive species.
We note the discrepancy between the DNA extraction performance on mock communities and fecal samples in our study as well as the benchmark study (Costea et al). Both studies have demonstrated that the fecal DNA samples extracted by protocol Q displayed higher relative abundances of multiple gram-positive species than other methods.

As we note in our response to the Reviewer 1, so far, it is still challenging to create a mock microbial community that can mimic human feces. The two reasons are that 1) most of the gut residents are anaerobic and hard to culture and 2) a simple mixture of

microbial species will not reflect the complex, highly variable chemical and physical properties of human feces, which might potentially impact the activities of enzymes for downstream library construction and sequencing. Thus, extraction performance based on an MMC will not precisely and unbiasedly reflect extraction performance on human fecal samples.

Also, for both studies, quantitative performance on extracting human gut microbiome between protocols has been interpreted based on bacterial relative abundance but not absolute abundance, which we measured in the mock. Further efforts are still needed to quantify absolute microbial abundances in fecal mock materials with a mixture of both abundant gut microbes and non-living fecal compounds, and in real fecal samples to accurately assess the quantification biases of different protocols.

We have extensively revised our manuscript to discuss the limitations of our study as well as previous ones (in line 251-270), and we hope we have addressed the reviewer's concerns about the discrepancy of the DNA extraction performance on mock communities and fecal samples.

2. As described by the authors, very low levels of mycobiome only in few fecal samples were detected with tested protocols and fungal sequence reads were only identified in one sample both MP and Q protocols. Therefore, I am not sure we can determine that MP is a more effective method for human metagenomic analysis than Q protocol, even though MP showed greater efficacy in mock sample analysis than Q protocol.

Response:
First, a measurable fungal abundance was only detected in few samples in this study. However, these observations do not necessarily imply that there were no fungal genomes extracted in human feces by the six protocols.
Previous studies have demonstrated very low levels of fungi in human fecal samples[5–7]. As reported by Richard et al, the number of fungi in faces has been shown to be far lower than that of bacteria, with 105 to 106 fungal cells per gram of fecal matter compared with 1011 bacterial cells per gram [7]. In addition, the genome sizes of fungi are also much greater than that of bacteria. Thus, a much greater amount of sequencing data than we generated in the current study is needed to evaluate the performance of fecal mycobiome extraction across protocols. Amplicon-based approaches (18S rRNA-based or ITS-based) seem still to be more cost-effective and appropriate in order to assess the mycobiome in human fecal samples, and such amplicon-based approaches have been successfully applied in several studies[8–10].

Second, our study has shown a positive correlation between the bead size and extraction efficiency of yeast DNA (Figure 2d,f and Supplementary Figure 2), and the two protocols (MN using 0.6-0.8mm beads, and ZYMO using a mixture of 0.1&0.5 beads) have shown better performance in recovering genome coverage and abundance of the two yeast strains than other three protocols using 0.1mm beads (MetaHIT, MP and Q). We concur that a limitation of our study is that we did not apply amplicon-based sequencing to assess whether the performance of extracting fungi DNA using the different protocols is the same using mock or fecal samples.

We suggest researchers to consider using protocols with larger beads when their research interests are mainly focused on the mycobiome. Due to the limited sequencing depth of mycobiome from our shotgun metagenomic datasets, we were not able to compare the extracting performance of mycobiome between the MP and Q, or between any two protocols. We have modified our results (in line 188-189 and discussions (in line 243-250) to clearly describe our findings to help the reviewers and readers better understand our results.
References
1. Costea PI, Zeller G, Sunagawa S, Pelletier E, Alberti A, Levenez F, et al. Towards standards for human fecal sample processing in metagenomic studies. Nature Biotechnology [Internet]. Nature Publishing Group; 2017;35:1069–76. Available from: http://dx.doi.org/10.1038/nbt.3960
2. Schrader C, Schielke A, Ellerbroek L, Johne R. PCR inhibitors - occurrence, properties and removal. Journal of Applied Microbiology. 2012;113:1014–26.
3. Orpana AK, Ho TH, Stenman J. Multiple heat pulses during PCR extension enabling amplification of GC-rich sequences and reducing amplification bias. Analytical

	Chemistry. 2012;<br>4. Laursen MF, Dalgaard MD, Bahl MI. Genomic GC-content affects the accuracy of 16S rRNA gene sequencing bsed microbial profiling due to PCR bias. Frontiers in Microbiology. 2017;<br>5. Huffnagle GB, Noverr MC. The emerging world of the fungal microbiome. Trends in Microbiology. 2013.<br>6. Sam QH, Chang MW, Chai LYA. The fungal mycobiome and its interaction with gut bacteria in the host. International Journal of Molecular Sciences. 2017.<br>7. Richard ML, Sokol H. The gut mycobiota: insights into analysis, environmental interactions and role in gastrointestinal diseases. Nature Reviews Gastroenterology and Hepatology [Internet]. Springer US; 2019;16:331–45. Available from: http://dx.doi.org/10.1038/s41575-019-0121-2<br>8. Frau A, Kenny JG, Lenzi L, Campbell BJ, Ijaz UZ, Duckworth CA, et al. DNA extraction and amplicon production strategies deeply influence the outcome of gut mycobiome studies. Scientific Reports [Internet]. Springer US; 2019;9:1–17. Available from: http://dx.doi.org/10.1038/s41598-019-44974-x<br>9. Nash AK, Auchtung TA, Wong MC, Smith DP, Gesell JR, Ross MC, et al. The gut mycobiome of the Human Microbiome Project healthy cohort. Microbiome. 2017;<br>10. Zuo T, Wong SH, Cheung CP, Lam K, Lui R, Cheung K, et al. Gut fungal dysbiosis correlates with reduced efficacy of fecal microbiota transplantation in Clostridium difficile infection. Nature Communications. 2018;
**Additional Information:**	
**Question**	**Response**
Are you submitting this manuscript to a special series or article collection?	No
**Experimental design and statistics**<br><br>Full details of the experimental design and statistical methods used should be given in the Methods section, as detailed in our Minimum Standards Reporting Checklist. Information essential to interpreting the data presented should be made available in the figure legends.<br><br>Have you included all the information requested in your manuscript?	Yes
**Resources**<br><br>A description of all resources used, including antibodies, cell lines, animals and software tools, with enough information to allow them to be uniquely identified, should be included in the Methods section. Authors are strongly encouraged to cite Research Resource Identifiers (RRIDs) for antibodies, model organisms and tools, where possible.	Yes

Powered by Editorial Manager® and ProduXion Manager® from Aries Systems Corporation

Have you included the information requested as detailed in our Minimum Standards Reporting Checklist?	
**Availability of data and materials**<br><br>All datasets and code on which the conclusions of the paper rely must be either included in your submission or deposited in publicly available repositories (where available and ethically appropriate), referencing such data using a unique identifier in the references and in the “Availability of Data and Materials” section of your manuscript.<br><br>Have you have met the above requirement as detailed in our Minimum Standards Reporting Checklist?	Yes

Powered by Editorial Manager® and ProduXion Manager® from Aries Systems Corporation

Manuscript with DOI
Click here to access/download;Manuscript;2020-03-24-GIGA-D-20-00011-manuscript with DOI.docx

Assessment of fecal DNA extraction protocols for metagenomic studies

Fangming Yang[1,2†], Jihua Sun[3,4†], Huainian Luo[3], Huahui Ren[2,4], Hongcheng Zhou[5], Yuxiang Lin[2], Mo Han[2,4], Bing Chen[2], Hailong Liao[5], Susanne Brix[6], Junhua Li[2,7], Huanming Yang[2,8], Karsten Kristiansen[2,4*], Huanzi Zhong[2,4*]

1 School of Future Technology, University of Chinese Academy of Sciences, Beijing 101408, China.

2 BGI-Shenzhen, Shenzhen, 518083, China.

3 BGI Europe A/S, COBIS, 2200 Copenhagen, Denmark.

4 Laboratory of Genomics and Molecular Biomedicine, Department of Biology, University of Copenhagen, 2100 Copenhagen, Denmark.

5 China National Genebank, Shenzhen, 518120, China.

6 Department of Biotechnology and Biomedicine, Technical University of Denmark, 2800 Kgs. Lyngby, Denmark.

7 School of Biology and Biological Engineering, South China University of Technology, Guangzhou 510006, China.

8 James D. Watson Institute of Genome Sciences, Hangzhou 310058, China.

* Correspondence: Karsten Kristiansen, kk@bio.ku.dk; Huanzi Zhong, zhonghuanzi@genomics.cn

† Equal contributor

Abstract

Background: Shotgun metagenomic sequencing has greatly improved our understanding of the human gut microbiota. Efforts have been made to evaluate the performance of various DNA extraction methods to recommend protocols that robustly and most accurately reflect the original microbial community structures. However, so far recommended standardized bacterial DNA extraction protocols still may be improved, especially considering future demands in relation to time and cost dealing with samples from very large human cohorts. Additionally, fungal DNA extraction performance has so far been little investigated.

Results: Here, we compared six DNA extraction protocols, MagPure Fast Stool DNA KF Kit B (MP), Macherey Nagel™ NucleoSpin™®Soil kit (MN), Zymo Research Quick-DNA™ Fecal/Soil Microbe kit (ZYMO), MOBIO DNeasy PowerSoil kit (PS), the manual non-commercial protocol MetaHIT, and the recently published protocol Q using one microbial mock community (MMC) (containing eight bacterial and two fungal strains) and fecal samples. All samples were manually extracted and subjected to shotgun metagenomics sequencing. Evaluation of the results obtained by extracting DNA from the MMC and human fecal samples revealed high reproducibility within all six protocols, but microbial extraction efficiencies varied between protocols. Evaluation of results using the MMC demonstrated that bead size was a determining factor for fungal and bacterial DNA yields. Evaluation of human fecal samples revealed that the bacterial extraction performance of protocol MP matched that of the standardized protocol Q, but consumed less time and was more cost-effective. Extraction using the protocol PS resulted in a significantly higher ratio of gram-negative to gram-positive bacteria than other protocols, which might potentially contribute to reported gut microbial differences between healthy US, Chinese and Danish adults, where fecal DNA samples were extracted using protocols PS and MetaHIT.

Conclusions: We emphasize the importance of bead size selection for bacterial and fungal DNA extraction. More importantly, we demonstrate that the novel, time- and cost-effective protocol MP in terms of consistency and performance is equal to the standardized protocol Q, and we recommend the use of MP for further large-scale human gut metagenomic studies.

Keywords: DNA extraction, gut microbiota, human fecal sample, shotgun metagenomic sequencing

Background

The adult human gut harbors highly complex and diverse microbial communities, including bacteria, archaea, fungi, viruses and protozoa [1]. The composition of the gut bacterial community has been demonstrated to exhibit associations with multiple human diseases, including type 2 diabetes [2–4], obesity [5–7], and colorectal cancer [8,9]. However, many studies have shown how different experimental processing pipelines impact on the results [10,11], and how especially DNA extraction affects the quantitative characterization of bacterial components [11–13], emphasizing the need for a standardized and robust protocol for profiling of the gut microbiota in order to enable true comparison between studies.

During the past two decades, PCR-based amplicon sequencing, a flexible and cost-effective method to determine microbial composition, has greatly improved our understanding of human microbiome. However, considering the known effects of PCR conditions on amplification biases such as primers, specific hypervariable regions, and annealing temperature [14,15], amplicon sequencing is insufficient for accurately evaluating the quantitative performance of bacterial DNA extraction protocols. In comparison, shotgun metagenomic sequencing is a more accurate tool for analyzing the microbiota. A recent shotgun sequencing-based benchmark study has comprehensively investigated bacterial extraction performances of 21 fecal DNA extraction protocols, including widely used extraction kits and non-kit-based protocols [11]. By evaluation of DNA quantity and quality, community diversity, and extraction efficiency of gram-positive and gram-negative bacteria, this study has proposed protocol Q, a manual protocol based on a modified version of Qiagen's QIAamp® DNA Stool Mini Kit, as a standard protocol for human fecal bacterial DNA extraction [11]. However, there is still room for improvement to establish less labor-intensive and more cost-effective alternative standardized protocols, especially for large-scale gut microbiome studies. Additionally, assessment of fungal DNA extraction performance in fecal samples, the often neglected important players in the overall gut microbiome [16–18], is still scarce.

In this study, we assessed the DNA extraction performance of six protocols on a microbial mock community (MMC) comprising eight bacterial and two yeast strains, and on fecal samples from six healthy human individuals, using the protocol Q as a reference method. Based on extractions of the MMC, we established a positive correlation between the bead size and extraction efficiency of yeast DNA, providing information for the selection of appropriate DNA extraction protocols for fungal-related studies. Based on extractions from human fecal samples, we found that a time- and cost-effective kit-based protocol, protocol

MP, exhibited bacterial DNA extraction performance similar to protocol Q regarding DNA yield, bacterial community diversity, and relative abundances of gram-positive and gram-negative bacteria.

Data Description

Our study applied six DNA extraction protocols (**Supplementary Table S1**) on two types of biological samples, including a 10-species microbial mock community (MMC) and human fecal samples from six healthy individuals (**Fig. 1, Methods**). The MMC (Catalog No. D6300), containing cells of eight bacteria (each making up 12%) and two yeast strains (each contributing with 2%), was purchased from ZYMO Research (**Fig. 1**). Among the six protocols, three kit-based methods including MagPure Fast Stool DNA KF Kit B (MP), Macherey Nagel™ NucleoSpin™®Soil kit (MN) and Zymo Research Quick-DNA™ Fecal/Soil Microbe kit (ZYMO)) were not thoroughly evaluated in the previous studies [19,20]. In addition, we also included three protocols used in the benchmark study [11], including protocol Q, MOBIO DNeasy PowerSoil kit (PS), and a non-kit-based manual protocol adopted by MetaHIT (METAgenomics of the Human Intestinal Tract consortium) for evaluating the reproducibility of the DNA extraction protocols. DNA of all samples was manually extracted in the laboratory of BGI Europe A/S, COBIS, Copenhagen, Denmark. All six protocols used in this study included a step of mechanical cell disruption by bead beating (See full standard operating procedure (SOP) of each protocol in **Supplementary File F1**). For each protocol, six technical replicates were generated from the MMC and each human fecal sample. In total, 233 qualified DNA samples (36 MMC extractions and 197 human fecal DNA extractions) were subjected to shotgun sequencing and further quantitative analyses (**Supplementary Table S2**).

Analyses

Assessment of processing time and DNA yield

Among the six protocols, four kit-based protocols (MP, MN, ZYMO and PS) were much more effective in relation to DNA processing time than the two manual protocols (Q and MetaHIT) (40~100 minutes vs. 156~380 minutes per extraction) (**Supplementary Table S1**). We next compared DNA yields between the protocols. Using the amount of starting material as given in the "Methods" section, extraction of MMC yielded on average 0.77μg DNA per sample, whereas extraction of human fecal samples on average yielded 4.31μg DNA per sample (**Supplementary Table S2**). The PS kit gave significantly lower DNA yields than protocol

MN and ZYMO on the MMC. The PS kit also showed significantly lower DNA yields than all other protocols on human fecal samples except for protocol ZYMO (Benjamini-Hochberg, BH-adjusted *Dunn's* $p < 0.05$, **Supplementary Table S3, Supplementary Figure S1**) in line with previous observations [12,21–23]. On the other hand, we found inconsistent performances of protocol Q in retrieving DNA from the MMC and human fecal samples. Protocol Q delivered significantly lower DNA yields than protocols MP, MN and ZYMO on the MMC (BH-adjusted *Dunn's* $p < 0.05$, **Supplementary Figure S1a**), but showed similar DNA yields on human fecal samples when compared with protocols MP, MN and ZYMO (BH-adjusted *Dunn's* $p > 0.05$, **Supplementary Figure S1b**).

Evaluation of DNA extraction protocols on the mock community

We first estimated the relative abundances of the bacterial and yeast strains obtained using the six protocols and based on the reference genomes of the MMC (see details in the "Methods" section). Focusing on the eight bacterial strains, we found that except for the protocol MetaHIT, six replicates from each of the remaining five protocols tended to consistently underestimate gram-positive bacteria including *Staphylococcus aureus*, *Enterococcus faecalis*, *Listeria monocytogenes* and *Bacillus subtilis*, but overestimated all three gram-negative members (*Salmonella enterica*, *Escherichia coli* and *Pseudomonas aeruginosa*) (**Fig. 2a**). By combining results from all eight bacterial strains, we observed that the protocol MP showed a relatively higher mean accuracy in bacterial abundance estimations than the other protocols (mean estimation error, MEE: 0.22, **Fig. 2c**), followed by protocol MetaHIT and protocol MN (MEE < 0.5, **Fig. 2c**). All six protocols provided almost complete genome recovery of the eight bacterial strains (genome coverage, mean ± sd: 98.90% ± 1.5%, **Fig. 2e**). However, the recovery of the two yeast genomes (*Saccharomyces cerevisiae* and *Cryptococcus neoformans*) was much lower than that of the bacterial genomes and varied considerably between protocols (genome coverage, mean ± sd: 62.11% ± 31.52%, **Fig. 2f**). Of note, two protocols using relatively large beads (MN with 0.6~0.8mm diameter beads and ZYMO with 0.5mm diameter beads) ensured higher relative abundances and genome coverages of the two yeast strains than protocols with 0.1mm diameter beads (MP, MetaHIT and Q) (**Fig. 2b, d, f**). Additionally, we also observed very low intra-protocol variabilities in performance on microbial abundance estimation (**Fig. 2a, b**) and genome recovery (**Fig. 2e, f**), indicating high reproducibility of each protocol.

Asking whether there was a robust positive correlation between bead size and fungal DNA yield, we subsequently conducted a bead size-dependent extraction experiment. Briefly, we tested protocol MP using three types of bead conditions (500μL of Φ0.1mm; 250μL of Φ0.1mm plus 250μL of Φ0.6~0.8mm; 500μL of

Φ0.6~0.8mm) on cell cultures of *Escherichia coli* K-12 MG1655 (*E. coli* MG1655), *Saccharomyces cerevisiae* BY4741 (*S. cerevisiae* BY4741), and a mixture of *E. coli* MG1655 and *S. cerevisiae* BY4741 (2:1, v/v), with ten extraction replicates per condition. By quantifying and comparing DNA yields between groups (**Supplementary Table S4**), we found that protocol MP using beads of 0.6~0.8mm diameter either alone or in combination with beads of 0.1mm diameter gave significantly higher DNA yields of *S. cerevisiae* than the protocol using beads of 0.1mm diameter (Wilcoxon rank-sum test, $p < 0.05$, **Supplementary Figure S2**). By contrast, the protocol using beads of 0.1mm diameter showed significantly higher DNA yields of *E. coli* than the protocol containing only beads of 0.6~0.8mm diameter or the combination of these beads with beads of 0.1mm diameter (Wilcoxon rank-sum test, $p < 0.05$, **Supplementary Figure S2**), indicating the difficulty for simultaneously unbiased bacterial and fungal DNA extraction.

Evaluation of the DNA extraction protocols on human fecal samples

We next evaluated the intra- and inter-protocol performance on human fecal samples. Spearman's rank correlation analysis revealed high coefficient values between technical replicates at both gene (**Supplementary Figure S3a**, averaged Spearman's Rho = 0.875) and species level (**Fig. 3a**, averaged Spearman's Rho = 0.964). Likewise, the average Bray-Curtis dissimilarities between intra-protocol replications were 0.142 at the gene level (**Supplementary Figure S3b**) and 0.046 at the species level (**Fig. 3b**). These results suggest high intra-protocol reproducibility in the quantification of relative abundance of human gut microbial genes and species.

There were no significant differences in microbial richness between protocols at the gene and the species level (Kruskal-Wallis test, $p > 0.05$, **Supplementary Figure S4a, b**, **Supplementary Table S5**). However, we observed significantly lower microbial diversity in samples extracted by protocol Q compared to protocols MN and ZYMO, the two large bead-based protocols (BH-adjusted *Dunn's p* < 0.05, **Supplementary Figure S4c, d, Supplementary Table S5**). Inter-protocol analyses further demonstrated smaller values of Spearman's rank coefficients (**Supplementary Figure S3c, Fig. 3c**) and greater microbial Bray-Curtis dissimilarities (**Supplementary Figure S3d, Fig. 3d**) of microbial profiles between samples extracted by the PS and protocol Q compared to those between other protocols and protocol Q. On the other hand, regardless of DNA extraction protocols, datasets from the same individual were grouped on a principal component analysis (PCA) plot (**Fig. 3e**) and showed greater dissimilarities between each other than between intra- or inter-protocol replications (**Fig. 3f**). This is in agreement with the previous notion that inter-individual variation exceeds the variation resulting from different protocols [13,22,24–26].

Based on cluster analysis, we further revealed larger species compositional dissimilarities between PS-extracted samples and samples extracted using the other protocols (**Fig. 4a**). In addition, we found comparable species composition comparing samples extracted by protocols MP and Q, and between samples extracted using protocols MN and ZYMO, respectively (**Fig. 4a**). We assessed differences in the quantification performance of individual species between protocols by confining our analyses to 210 common species of at least 20% occurrence among samples (see details in the "Methods" section). Of note, 72.38% (152 of 210) differed significantly in relative abundance between at least two protocols (Kruskal-Wallis test, BH-adjusted $p < 0.05$, **Supplementary Table S6**). In line with the benchmark study [11], the relative abundances of multiple gram-positive species were significantly higher in Q-extracted samples than those extracted using the protocol PS, including species from the genera *Bifidobacterium*, *Collinsella*, *Streptococcus*, and *Parvimonas* (**Fig. 4c**, BH-adjusted *Dunn's* $p < 0.05$). By contrast, the relative abundances of multiple gram-negative species annotated to the genera *Bacteroides*, *Prevotella*, and *Haemophilus* were consistently and significantly lower in Q- and MP-extracted samples compared with those extracted using the other protocols (**Fig. 4b**, BH-adjusted *Dunn's* $p < 0.05$).

Furthermore, we found that PS-extracted samples exhibited significantly lower abundances of gram-positive species but higher abundances of gram-negative species than samples extracted by using the other five protocols (**Supplementary Figure S5**, BH-adjusted *Dunn's* $p < 0.05$). By plotting the abundance distributions of selected abundant gram-positive and gram-negative gut species, including *Bifidobacterium adolescentis* (Gram-positive, G+), *Bifidobacterium longum* (G+), *Faecalibacterium prausnitzii* (G+), *Collinsella intestinalis* (G+), *Streptococcus anginosus* (G+), *Streptococcus cristatus* (G+), *Alistipes putredinis* (Gram-negative, G-), *Bacteroides coprocola* (G-), *Bacteroides dorei* (G-), *Bacteroides dorei/vulgatus* (G-), *Bacteroides ovatus* (G-), and *Prevotella copri* (G-), we found that species-related quantitative biases between PS and the other protocols were consistent among all individuals (**Supplementary Figure S6**). We further replicated a consistent and significant enrichment of 52 species comparing metagenomic datasets of PS-extracted samples and the three Qiagen kit-based protocols from the benchmark study (**Supplementary Figure S7**, BH-adjusted *Dunn's* $p < 0.05$) [11].

In the current shotgun metagenomic datasets, we only detected very low levels of fungi species (0.03% ~2.32%) in fecal samples from individual C and F using MetaPhlAn2 [27] (**Supplementary Table S7**). However, by extraction of human fecal samples, we did not observe the same clear relation between bead size and fungal DNA extraction yield as observed using the MMC, further underscoring the difficulties in choosing an extraction protocol providing a robust, accurate representation of both bacterial and fungal DNA.

DNA extraction biases may contribute to reported country-specific signatures

To investigate to what extent differences between the performance of DNA extraction protocols might influence reported results on country-specific gut microbial signatures, we compared available shotgun metagenomic datasets of healthy Chinese (n=60) and Danish adults (n=100) (protocol MetaHIT) [28], to healthy US adults (n=167) from the Human Microbiome Project (HMP, protocol PS) [29]. Samples from the three countries separated clearly from each other in principal coordinate analysis (PCoA) plots (**Fig. 5a**). Still, we noted that species profiles of Chinese and Danish adults, whose fecal samples were extracted using the protocol MetaHIT, exhibited less Bray-Curtis dissimilarity than that observed between US adults (**Fig. 5b**). Furthermore, we found that PS-extracted US samples exhibited significantly higher abundances of multiple gram-negative species and lower abundances of gram-positive species than those of MetaHIT-extracted samples from both Chinese and Danish adults (BH-adjusted *Dunn's p* <0.05, **Fig. 5c**). Such quantitative differences may contribute to a significantly higher Bacteroidetes to Firmicutes ratio in US adults as compared to Chinese and Danish adults (**Fig. 5d**). While more detailed comparisons of samples from different countries need to be scrutinized using identical extraction and sequencing protocol to determine to what extent these differences truly reflect country/ethnicity-dependent differences. These observations emphasize that cautions must be taken in interpreting gut microbial findings observed using different DNA extraction methods, and that standardized extraction protocols are needed for reliable comparison of samples from different ethnic groups.

Discussion

In this study, six DNA extraction protocols were assessed using both MMC and human fecal samples subjected to shotgun metagenomics sequencing. Experiments using MMC revealed that protocols with smaller bead size yielded higher bacterial DNA recovery, whereas protocols with larger bead size yielded higher fungal DNA recovery. However, the latter could not be replicated using human fecal samples. Assessment of human fecal samples showed a varied extraction efficiency of gram-positive and gram-negative species between protocols, especially between the PS and the other protocols. We propose that such protocol-dependent differences might contribute to the reported gut microbial differences between cohorts from different countries and of different ethnicity. We report that protocol MP, a time- and cost-effective method, compared to the other protocols evaluated in this study, exhibited an extraction performance in characterizing and quantifying bacterial

community similar to the recently proposed standard protocol Q [11]. Therefore, we propose protocol MP as a robust and alternative standard protocol for human fecal DNA extraction in future large-scale metagenomics studies. However, we emphasize that the performance of the extraction protocols tested on fecal samples in the present study needs to evaluate for use on other human-related samples (e.g., saliva and skin) as microbial composition as well as physical and chemical properties of such samples are quite distinct from those of fecal samples. Future large-scale metagenomics projects will need to employ automated DNA extraction. Thus, one limitation of this study is that the performance of the MagPure kit in relation to a robotized extraction system was not evaluated, and further efforts are required to assess the stability and consistency between manual and automated DNA extraction using the MagPure kit.

With a known species composition, MMC allowed us to investigate the DNA extraction efficiency of both bacteria and fungi. All six protocols in this study included a bead-beating step, the most effective mechanical lysis method [13,24,30–32] with different sizes and composition of beads. Regardless of technical differences between the protocols, we found that two protocols (MN and ZYMO) with large beads (0.5~0.8mm) showed significantly better performance in the recovery of fungal genomes and theoretical abundances than other protocols with beads of 0.1mm diameter. Of note, our experiments on a mock community of *E. coli* MG1655, *S. cerevisiae* BY4741, and a simple mixture of *E. coli* MG1655 and *S. cerevisiae* BY4741 showed that a large bead-based method (Φ0.6~0.8mm) secured high extraction efficiency of yeast, but simultaneously sacrificed the extraction efficiency of bacteria. Therefore, extraction methods with combinations of beads of different sizes seem warranted for further studies in order to possibly achieve an accurate and reliable representation of microbial communities with both bacteria and fungi even though the combination of small and large bead sizes used in the present study were unable to improve simultaneous recovery of bacterial and fungal DNA. We were unable to evaluate the fungal DNA extraction efficiency using human fecal samples due to low levels of detection of fungal taxa from the six volunteers in the current shotgun metagenomic datasets. It has been demonstrated that the number of fungi in human feces is far less than that of bacteria[1,33–36], with 10^5 to 10^6 fungal cells per gram of feces compared with 10^{11} bacterial cells per gram [36]. In addition, the genome sizes of fungi are much larger than those of bacteria. Thus, a much greater amount of sequencing data than we generated in the current study is needed to evaluate the performance of fecal mycobiome extraction across protocols. Amplicon-based approaches (18S rRNA-based or ITS-based) seem still to be more cost-effective and appropriate in order to assess and interpret the mycobiome in human fecal samples, and such amplicon-based approaches have been successfully applied in several studies[35,37,38].

Another observation was the inconsistency in relation to the extraction efficiency of gram-positive and gram-negative species using MMC and human fecal samples extracted by the same protocols. For instance, except for MetaHIT, all other protocols including protocol Q underestimated the relative abundance of four gram-positive strains (*S. aureus*, *E. faecalis*, *L. monocytogenes* and *B. subtilis*) and overestimated the relative abundance of the tested three gram-negative strains (*S. enterica*, *E. coli* and *P. aeruginosa*) in the MMC samples. Likewise, the benchmark study [11] showed that regardless of whether DNA was extracted from a mock community or from a fecal sample with a spike-in mock community, protocol Q underestimated the abundances of gram-positive bacteria including *Clostridium perfringens*, *C. difficile* and *Lactobacillus plantarum* and overestimated the abundances of three gram-negative members including *S. enterica*, *Prevotella melaninogenica* and *Fusobacterium nucleatum*. By contrast, human fecal DNA samples extracted by protocol Q displayed better performance in the quantification of gram-positive species than the other protocols. In addition, the mock communities from both studies were both composed of human pathogenic bacteria or bacteria isolated from a non-human environment, which do not reflect the human gut microbial composition. Furthermore, such simple mixtures of bacteria and fungi do not contain other compounds in feces such as humic acids, polysaccharides, bile acids and lipids, which might potentially inhibit the activity of enzymes used for subsequent PCR-based library construction and sequencing[39]. Thus, extraction performance based on MMC may not precisely and unbiasedly reflect extraction performance on human fecal samples. Finally, for both studies, quantitative performance on extracting human gut microbiome between protocols has been interpreted based on relative bacterial abundances but not absolute abundance, which we measured in the MMC. Further efforts are still needed to quantify absolute microbial abundances in fecal mock materials with a mixture of both abundant gut microbes and non-living fecal compounds, and in real fecal samples to accurately assess the quantification biases of different protocols.

Potential implications

DNA extraction protocols affect the outcome of metagenomics studies, and standardized, validated, and cost and time-effective protocols are needed for large-scale metagenomics projects. We compared six commonly used DNA extraction protocols using one microbial mock community and fecal samples. Evaluation of the results based on shotgun metagenomic sequencing revealed the importance of bead sizes for bacterial and fungal DNA extraction. Microbial extraction efficiencies varied between protocols. The performance of the novel MagPure Fast Stool DNA KF Kit B matched that of the recommended standardized protocol Q, but

consumed less time, was more cost-effective, and is recommended for large-scale studies.

Methods

Sample collection and preparation

Microbial mock community.

ZymoBIOMICS Microbial Community Standard, Catalog No. D6300 (Microbial Mock Community, MMC) was obtained from Zymo research. The mock community contains eight bacteria with the same abundance: *Staphylococcus aureus*, *Enterococcus faecalis*, *Listeria monocytogenes*, *Bacillus subtilis*, *Salmonella enterica*, *Lactobacillus fermentum*, *Escherichia coli*, *Pseudomonas aeruginosa* and two yeasts species, also with the same abundance: *Saccharomyces cerevisiae* and *Cryptococcus neoformans*. The theoretical relative abundance of each bacterial strain is 12% and that of each fungal strain 2% (**Fig. 1**).

Human fecal sample collection

Six healthy volunteers including one four-year-old child and five adults (32 ± 3 years old), were recruited from BGI Europe employees or family members, Copenhagen, Denmark (See detailed information in **Supplementary Table 2**). All volunteers or the guardian consented to provide fecal samples for this study. About 10-15 grams of stool were freshly collected by participants at home by using a 50 mL sterile conical tube, and copies of printed instructions were used to guide the adult volunteers or the child's legal guardian for self-collection of fecal samples. After collection, samples were stored at -20 °C and transported to the laboratory on the second day with ice packs in forty minutes. Then, each sample was diluted with 1~1.5 volumes (15 mL) of Tris-EDTA (TE, 10 mM Tris pH 8.0 and 1 mM EDTA, Thermo Fisher Scientific) buffer, homogenized and divided into 36 aliquots (500 μL per aliquot). All stool aliquots were stored at -80 °C before DNA extraction.

DNA extraction, library preparation and sequencing

All DNA extraction experiments examining the six different protocols were performed manually by the same technician at the BGI Europe laboratory, Copenhagen, Denmark, and the bead-size experiments were performed at BGI-Shenzhen. The DNA extraction was conducted in accordance to the manufacturer's

instructions or protocols provided (See full SOP of each protocol in **Supplementary File F1**. For both mock community and human fecal samples, six technical replicates were generated using each protocol. The DNA concentration was detected by Qubit® 2.0 fluorometer (Invitrogen). Considering the different starting volume used in each protocol, we normalized the DNA yield to the volume of starting material.

All 36 DNA samples from the MMC were successfully extracted by the six extraction protocols, and library construction and sequencing were successful for all these 36 DNA sample. Six fecal samples extracted using protocol PS (individual E) and 13 fecal samples extracted using protocol ZYMO (six of individual A, six of individual C, and one of individual F) that yielded less than 500ng and failed for library preparation, were removed from further processing. Library preparation and shotgun metagenomic sequencing were performed on the BGISEQ-500 platform using the paired-end (PE)100 mode [40]. Low-quality reads and human-derived reads were filtered to generate high-quality non-human reads as described previously [40], resulting in an averaged proportion of high-quality non-human reads of 94.33% per sample (including MMC and human fecal samples, coefficient of variation, CV%=6.63%) (**Supplementary Table S2**). In total, 233 shotgun metagenomic datasets from 36 mock community DNA extractions and 197 human fecal DNA extractions were generated and evaluated for the performance of the six protocols **(Supplementary Table S2)**.

Comparison of DNA extraction kits using mock communities

The ten microbial reference genomes of the MMC are available at ZymoBIOMICS.STD.genomes.ZR160406.zip. To minimize the potential impacts of sequencing depth on quantitative and qualitative assessment of the composition of the MMC, we randomly downsized each sample to 20 million high-quality paired reads and aligned the reads to the reference genomes using SOAP 2.22 (m=0, x=1000, r=1, l=30, M=4, S, p=6, v=5, S, c=0.95).

For all protocols, the total mapping ratio, defined as a ratio of the total number of mapped reads to the total number of high-quality reads, reached 98.32% on average (CV% = 0.27%). The relative abundance of each strain was calculated as a ratio of the number of mapped reads onto the reference genome to the total number of mapped reads onto all reference genomes. Genome coverage of each strain was calculated as the proportion of the genome reference covered by at least one read (SOAP coverage 2.7.7). For each species, the estimation error (EE) was used to represent the extraction bias, defined as

$$\mathrm{EE} = \frac{\text{Observed relative abundance} - \text{Theoretical relative abundance}}{\text{Theoretical relative abundance}}$$

For each protocol, the mean estimation error (MEE) was proposed to represent the extraction accuracy, that is

$$\text{MEE} = \overline{|EE|}$$

Where $\overline{|EE|}$ is the mean absolute value of the EE for all species in all technical replicates for each protocol.

A second-round DNA extraction experiment was performed to validate the positive correlation between bead sizes of DNA extraction protocols and DNA yield of yeast. Three types of bead conditions were assessed, including a) 500 μL of Φ0.1 mm beads, b) 250 μL of Φ0.1 mm beads mixed with 250 μL of Φ0.6~0.8 mm beads and c) 500 μL of Φ0.6~0.8 mm beads based on the MagPure Fast Stool DNA KF Kit B (MP). Three simple cell cultures were prepared for extraction testing, each in a volume of 1 mL, including a) only *Escherichia coli* K-12 MG1655 (*E. coli* MG1655), b) *Saccharomyces cerevisiae* BY4741 (*S. cerevisiae* BY4741) and c) a combination of 2/3 volume of *E. coli* MG1655 and 1/3 volume of *S. cerevisiae* BY4741. Extractions were carried out with ten technical replicates for each type of bead conditions on each kind of sample. In total, DNA yields of 90 extractions were measured and compared between the different bead conditions (**Supplementary Table S4**).

Comparison of DNA extraction kits using human fecal samples

Taxonomic profiling of shotgun metagenomic sequencing data from human fecal samples

High-quality and non-human reads were first aligned to the Integrated Gene Catalog (IGC) (SOAP 2.22 m=0, x=1000, r=2, l=30, M=4, S, p=6, v=5, S, c=0.95) [28]. On average, 79.67% (CV% = 2.03%) high-quality reads could be aligned to at least one gene from IGC. Uniquely mapped reads were then downsized to 20 million pairs for each sample to calculate gene relative abundance. The relative abundance of each species was computed based on the sum of relative abundance of genes annotated to the given species as described previously [28]. A total of 477 bacterial and archaeal species were identified in this study. We then confined our species-based comparison analyses to common species, which was defined as species with more than 100 annotated genes in all samples and with an occurrence in more than 20% of the samples.

Taxonomic profiling using MetaPhlAn2

The IGC based taxonomic annotation pipeline was previously developed based on 3,449 bacterial and archaeal taxa [28], lacking the information of fungal taxa. Aiming to evaluate to fungal quantitative performances in human fecal samples, we next performed taxonomic annotation and quantification using MetaPhlAn2 (version

2.7.0) [27] and generated microbial profiling including bacteria, eukaryotes, archaea and viruses for all 197 human fecal samples.

Alpha diversity and richness analyses

To estimate the richness and evenness of the microbial community in fecal samples, we calculated alpha diversity using the Shannon index at the gene and species level using the function diversity in the R package vegan (R version 3.4.1). Richness was defined as the number of observed genes or species in each sample.

Available shotgun metagenomic datasets from published studies

To validate the reliability of the observed difference between gram-positive and gram-negative species between different protocols, we selected 28 human fecal sample datasets from a published benchmark study[11], including eight datasets from DNA extracted by protocol PS and 20 datasets from DNA extracted by three Qiagen's QIAamp® DNA Stool Mini Kit-based protocols (eight datasets from Q-6, eight datasets from Q-9 and four datasets from Q-15) (**Supplementary Table S8**).

To investigate whether there are potential links between country-specific gut microbial signatures and the corresponding fecal DNA extraction protocols, shotgun metagenomic datasets of fecal DNA were retrieved from 60 healthy Chinese adults and 100 healthy Danish adults extracted using protocol MetaHIT [28] and from 167 healthy US adults (HMP) extracted using protocol PS [29]. Detailed information of these 327 metagenomic datasets is provided in **Supplementary Table S9**. IGC-based taxonomic assignment and quantification of all published datasets were performed as described above but without downsizing of the 327 country-specific signatures comparison datasets.

Statistical analyses

Correlation analysis

Spearman's correlation coefficient was calculated using function *cor.test* from the R package *stats* to estimate a rank-based measure of association.

Bray-Curtis dissimilarity and PCoA

Bray-Curtis dissimilarities at the gene and species level were calculated using the *vegdist* (method = "bray") function from the R package *vegan.* Principal coordinate analysis (PCoA) was performed to visualize the Bray-Curtis dissimilarities using the R package *ade*.

Kruskal-Wallis test

To determine which species differed significantly in abundance between samples extracted by different extraction protocols, and samples from different countries, the Kruskal-Wallis (KW) test was performed using the function *kruskal.test* from the R package *stats*. The Benjamini-Hochberg (BH) method was applied for adjustment of p values of the Kruskal-Wallis tests, using the *p.adjust* (method = "BH") function from R package *stats.* A BH-adjusted KW p-value below 0.05 was considered as statistically significance between multiple groups (≥3). Pairwise tests for multiple comparisons were followed by the Kruskal-Wallis test, using the function posthoc.kruskal.dunn.test from the R package PMCMR. *Dunn's p* values were calculated for each pairwise comparison and a BH-adjusted *Dunn's p*-value below 0.05 was considered as statistically significance between each two groups.

Availability of supporting data and materials

Metagenomic sequence data of the 36 microbial mock community samples and 197 fecal DNA samples have been deposited in the CNSA (https://db.cngb.org/cnsa/) of CNGBdb with accession number CNP0000497. 28 published shotgun metagenomic sequencing datasets from the benchmark study are available at the European Nucleotide Archive (ENA) under BioProject ERP016524. Published shotgun metagenomic sequencing datasets of 60 Chinese and 100 Danish adults are available at ENA with BioProject ID ERP004605 and ERP003612 respectively. Published shotgun metagenomic sequencing datasets of 167 US adults are available at the Sequence Read Archive (SRA; https://www.ncbi.nlm.nih.gov/sra) and the Database of Genotypes and Phenotypes (dbGaP; https://www.ncbi.nlm.nih.gov/gap) under the two studies: SRP002163 (BioProject PRJNA48479) and SRP056641 (BioProject PRJNA275349). Other data further supporting this work are openly available in the *GigaScience* database, GigaDB [41].

Additional files

Supplementary Table S1-9. Supplementary Tables S1-S9: **Table S1:** Key parameters of the six DNA extraction protocols used in this study. **Table S2:** Summary of metagenomic sequencing data of the 36 microbial mock community (MMC) samples and 197 human fecal samples. **Table S3:** Statistical differences of DNA yields of MMC and human fecal samples between protocols. **Table S4:** DNA yields of bacteria and

yeast using different bead conditions. **Table S5:** Statistical differences of Shannon index and richness at the gene and species level between DNA extraction protocols. **Table S6:** List of 152 common species that differ significantly in abundance between the six DNA extraction protocols. **Table S7:** Summary of taxonomic assignments of the 197 human fecal samples using MetaPhlAn2. **Table S8:** List of retrieved samples from a published benchmark study for comparison of protocol PS and three Q based protocols. **Table S9:** List of retrieved metagenomic samples from published studies for country-specific signatures comparison.

Supplementary File F1. Supplementary. Full SOP of six DNA extraction protocols.

Supplementary Figures S1-7. Supplementary Figures S1-S7

Abbreviations

MMC: Microbial mock community

TE: Tris-EDTA

SOP: Standard operating procedure

PE: Paired-end

CV: Coefficient of variation

EE: Estimation error

MEE: Mean estimation error

IGC: Integrated Gene Catalog

HMP: Human microbiome project

PCA: Principal component analysis

PCoA: Principal coordinate analysis

KW: Kruskal-Wallis

BH: Benjamini-Hochberg

G+: Gram-positive

G-: Gram-negative

Acknowledgements

We thank all the volunteers who participated in this study. We thank Yang Li and Ying Dai for technical assistance in the extraction experiments. We thank Chao Fang and Zhun Shi for discussions and providing useful analysis suggestions. We thank Dr. Dan Wang for helpful discussions and suggestions on the revised manuscript. We gratefully acknowledge colleagues at China National Gene bank for library preparation and shotgun sequencing experiments, and helpful discussions.

Funding

This research was funded by the National Science and Technology Major Project of China (No:2017ZX10303406) and Shenzhen Municipal Government of China (No. JCYJ20170817145809215).

Authors' contributions

H.Z., J.S., and K.K. designed the study. J.S. and H.L. performed fecal sample collection and DNA extraction experiments on ZYMO mock community and human fecal samples. F.Y., H. Zhou., M.H., B.C. and H. Liao designed and performed independent DNA extraction experiments with varied bead conditions on mock communities with *E. coli* and/or *S. cerevisiae*. H.Z. and J.S. designed and supervised the data analyses. F.Y., H.R. and Y.L. performed the metagenomic data analyses. F.Y. and J.S. wrote the first version of the manuscript. H.Z., J.L., S.B. and K.K. revised the manuscript. All authors participated in discussions and contributed to shape the manuscript. All authors read and approved the final manuscript.

Ethics approval and consent to participate

The study was approved by the institutional review board of BGI under ethical document BGI-R039-1. Participants in this study have written informed consent before sample collection.

Competing interests

The authors declare that they have no competing interests.

References

1. Qin J, Li R, Raes J, Arumugam M, Burgdorf KS, Manichanh C, et al. ARTICLES A human gut microbial gene catalogue established by metagenomic sequencing. 2010;464.

2. Qin J, Li Y, Cai Z, Li S, Zhu J, Zhang F, et al. A metagenome-wide association study of gut microbiota in type 2 diabetes. Nature [Internet]. Nature Publishing Group; 2012; Available from: http://dx.doi.org/10.1038/nature11450

3. Karlsson FH, Tremaroli V, Nookaew I, Bergström G, Behre CJ, Fagerberg B, et al. Gut metagenome in European women with normal, impaired and diabetic glucose control. Nature. 2013;498:99–103.

4. Forslund K, Hildebrand F, Nielsen T, Falony G, Le Chatelier E, Sunagawa S, et al. Disentangling type 2 diabetes and metformin treatment signatures in the human gut microbiota. Nature. 2015;

5. Le Chatelier E, Nielsen T, Qin J, Prifti E, Hildebrand F, Falony G, et al. Richness of human gut microbiome correlates with metabolic markers. Nature. 2013;500:541–6.

6. Cotillard A, Kennedy SP, Kong LC, Prifti E, Pons N, Le Chatelier E, et al. Dietary intervention impact on gut microbial gene richness. Nature. 2013;500:585–8.

7. Liu R, Hong J, Xu X, Feng Q, Zhang D, Gu Y, et al. Gut microbiome and serum metabolome alterations in obesity and after weight-loss intervention. Nature Medicine. 2017;23:859–68.

8. Zeller G, Tap J, Voigt AY, Sunagawa S, Kultima JR, Costea PI, et al. Potential of fecal microbiota for early-stage detection of colorectal cancer. Molecular systems biology. 2014;

9. Feng Q, Liang S, Jia H, Stadlmayr A, Tang L, Lan Z, et al. Gut microbiome development along the colorectal adenoma-carcinoma sequence. Nature communications. 2015;6:6528.

10. Quince C, Walker AW, Simpson JT, Loman NJ, Segata N. Corrigendum: Shotgun metagenomics, from sampling to analysis. Nature biotechnology. 2017.

11. Costea PI, Zeller G, Sunagawa S, Pelletier E, Alberti A, Levenez F, et al. Towards standards for human fecal sample processing in metagenomic studies. Nature Biotechnology [Internet]. Nature Publishing Group; 2017;35:1069–76. Available from: http://dx.doi.org/10.1038/nbt.3960

12. Wesolowska-Andersen A, Bahl M, Carvalho V, Kristiansen K, Sicheritz-Pontén T, Gupta R, et al. Choice of bacterial DNA extraction method from fecal material influences community structure as evaluated by metagenomic analysis. Microbiome [Internet]. 2014;2:19. Available from: http://www.microbiomejournal.com/content/2/1/19

13. Lim MY, Song EJ, Kim SH, Lee J, Nam Y Do. Comparison of DNA extraction methods for human gut microbial community profiling. Systematic and Applied Microbiology [Internet]. Elsevier GmbH.; 2018;41:151–7. Available from: http://dx.doi.org/10.1016/j.syapm.2017.11.008

14. Orpana AK, Ho TH, Stenman J. Multiple heat pulses during PCR extension enabling amplification of GC-rich sequences and reducing amplification bias. Analytical Chemistry. 2012;

15. Laursen MF, Dalgaard MD, Bahl MI. Genomic GC-content affects the accuracy of 16S rRNA gene sequencing bsed microbial profiling due to PCR bias. Frontiers in Microbiology. 2017;

16. Hallen-Adams HE, Suhr MJ. Fungi in the healthy human gastrointestinal tract. Virulence. 2017.

17. Huseyin CE, O'Toole PW, Cotter PD, Scanlan PD. Forgotten fungi-the gut mycobiome in human health and disease. FEMS microbiology reviews. 2017.

18. Paterson MJ, Oh S, Underhill DM. Host–microbe interactions: commensal fungi in the gut. Current Opinion in Microbiology. 2017.

19. Wagner AO, Praeg N, Reitschuler C, Illmer P. Effect of DNA extraction procedure, repeated extraction and ethidium monoazide (EMA)/propidium monoazide (PMA) treatment on overall DNA yield and impact on microbial fingerprints for bacteria, fungi and archaea in a reference soil. Applied Soil Ecology. 2015;

20. Maksimov P, Schares G, Press S, Fröhlich A, Basso W, Herzig M, et al. Comparison of different commercial DNA extraction kits and PCR protocols for the detection of Echinococcus multilocularis eggs in faecal samples from

foxes. Veterinary Parasitology [Internet]. Elsevier B.V.; 2017;237:83–93. Available from: http://dx.doi.org/10.1016/j.vetpar.2017.02.015

21. Mahmoudi N, Slater GF, Fulthorpe RR. Comparison of commercial DNA extraction kits for isolation and purification of bacterial and eukaryotic DNA from PAH-contaminated soils. Canadian Journal of Microbiology. 2011;

22. Kennedy NA, Walker AW, Berry SH, Duncan SH, Farquarson FM, Louis P, et al. The impact of different DNA extraction kits and laboratories upon the assessment of human gut microbiota composition by 16S rRNA gene sequencing. PLoS ONE. 2014;

23. Stinson LF, Keelan JA, Payne MS. Comparison of Meconium DNA extraction methods for use in microbiome studies. Frontiers in Microbiology. 2018;

24. Salonen A, Nikkilä J, Jalanka-Tuovinen J, Immonen O, Rajilić-Stojanović M, Kekkonen RA, et al. Comparative analysis of fecal DNA extraction methods with phylogenetic microarray: Effective recovery of bacterial and archaeal DNA using mechanical cell lysis. Journal of Microbiological Methods. 2010;81:127–34.

25. Mackenzie BW, Waite DW, Taylor MW. Evaluating variation in human gut microbiota profiles due to DNA extraction method and inter-subject differences. Frontiers in Microbiology. 2015;

26. McGaughey KD, Yilmaz-Swenson T, Elsayed NM, Cruz DA, Rodriguez RR, Kritzer MD, et al. Comparative evaluation of a new magnetic bead-based DNA extraction method from fecal samples for downstream next-generation 16S rRNA gene sequencing. PLoS ONE. 2018;

27. Truong DT, Franzosa EA, Tickle TL, Scholz M, Weingart G, Pasolli E, et al. MetaPhlAn2 for enhanced metagenomic taxonomic profiling. Nature Methods. 2015;12:902–3.

28. Li J, Wang J, Jia H, Cai X, Zhong H, Feng Q, et al. An integrated catalog of reference genes in the human gut microbiome. Nature Biotechnology. 2014;

29. Lloyd-price J, Mahurkar A, Rahnavard G, Crabtree J, Orvis J, Hall AB, et al. Strains , functions and dynamics in the expanded Human Microbiome Project. Nature Publishing Group [Internet]. Nature Publishing Group; 2017;550:61–6. Available from: http://dx.doi.org/10.1038/nature23889

30. Ariefdjohan MW, Savaiano DA, Nakatsu CH. Comparison of DNA extraction kits for PCR-DGGE analysis of human intestinal microbial communities from fecal specimens. Nutrition Journal. 2010;9:1–8.

31. Yuan S, Cohen DB, Ravel J, Abdo Z, Forney LJ. Evaluation of methods for the extraction and purification of DNA from the human microbiome. PLoS ONE. 2012;7.

32. Santiago A, Panda S, Mengels G, Martinez X, Azpiroz F, Dore J, et al. Processing faecal samples: A step forward for standards in microbial community analysis. BMC Microbiology. 2014;14:1–9.

33. Huffnagle GB, Noverr MC. The emerging world of the fungal microbiome. Trends in Microbiology. 2013.

34. Sam QH, Chang MW, Chai LYA. The fungal mycobiome and its interaction with gut bacteria in the host. International Journal of Molecular Sciences. 2017.

35. Nash AK, Auchtung TA, Wong MC, Smith DP, Gesell JR, Ross MC, et al. The gut mycobiome of the Human Microbiome Project healthy cohort. Microbiome. 2017;

36. Richard ML, Sokol H. The gut mycobiota: insights into analysis, environmental interactions and role in gastrointestinal diseases. Nature Reviews Gastroenterology and Hepatology [Internet]. Springer US; 2019;16:331–45. Available from: http://dx.doi.org/10.1038/s41575-019-0121-2

37. Frau A, Kenny JG, Lenzi L, Campbell BJ, Ijaz UZ, Duckworth CA, et al. DNA extraction and amplicon production strategies deeply influence the outcome of gut mycobiome studies. Scientific Reports [Internet]. Springer US; 2019;9:1–17. Available from: http://dx.doi.org/10.1038/s41598-019-44974-x

38. Zuo T, Wong SH, Cheung CP, Lam K, Lui R, Cheung K, et al. Gut fungal dysbiosis correlates with reduced efficacy of fecal microbiota transplantation in Clostridium difficile infection. Nature Communications. 2018;

39. Schrader C, Schielke A, Ellerbroek L, Johne R. PCR inhibitors - occurrence, properties and removal. Journal of Applied Microbiology. 2012;113:1014–26.

40. Fang C, Zhong H, Lin Y, Chen B, Han M, Ren H, et al. Assessment of the cPAS-based BGISEQ-500 platform for

metagenomic sequencing. GigaScience. 2018.

41. Yang F; Sun J; Luo H; Ren H; Zhou H; Lin Y; Han M; Chen B; Liao H; Brix S; Li J; Yang H; Kristiansen K; Zhong H: Supporting data for "Assessment of fecal DNA extraction protocols for metagenomic studies" GigaScience Database.2020. http://dx.doi.org/10.5524/100742.

Figures and Figure legends

Fig. 1 Schematic workflow of study design

Comparison of six DNA extraction protocols using a microbial mock community (MMC) and fecal samples from six individuals via shotgun metagenomics sequencing. Tables are showing strain information of the MMC (eight bacterial and two yeast strains) (top right) and six DNA extraction protocols (bottom right).

Fig. 2 Performance of the six different DNA extraction protocols on an MMC

a-b, Bar plot showing the mean observed relative abundances of eight bacteria (a) and two yeasts (b) using the six extraction protocols. Error bars showing the standard error of the mean relative strain abundance.

c-d, Estimation error (EE) of eight bacteria (c) or two yeasts (d) in all technical replicates for each protocol.

e-f, Genome coverage of eight bacteria (e) and two yeasts (f) using the six extraction protocols. Genome coverage is calculated as the proportion of the genome reference covered by at least one read.

Fig. 3 Intra- and inter-protocol consistency in species quantification using human fecal samples

a-b, Spearman's rho (a), and Bray-Curtis dissimilarities (b) between six technical replicates within each protocol.

c-d, Spearman's rho (c), and Bray-Curtis dissimilarities (d) between protocol Q and the five other protocols.

e, Principal component analysis (PCA) based on species profile. Colors indicate different protocols: light green, protocol Q; green, protocol MP; blue, protocol MN; purple, protocol ZYMO; orange, protocol MetaHIT; yellow, protocol PS. Different shapes indicate DNA samples from different individuals.

f, Box plots showing the inter-individual Bray-Curtis dissimilarities using the same protocol. Each panel indicates Bray-Curtis dissimilarities between samples from a given individual and that from others.

Fig. 4 Protocol-dependent differences in the relative abundance of gut bacterial species

a, Clustering of samples extracted by the different protocols based on species-level Bray-Curtis dissimilarities.

b-c, Heatmap showing gram-negative (b) and gram-positive (c) species that differ significantly in abundance between protocol Q and the other protocols. Color key indicates the mean rank of relative abundance of each species between comparisons in the Kruskal Wallis test. Pairwise comparisons using *Dunn*'s test were followed by the Kruskal Wallis test. Asterisks as indicators for statistical significance, *, BH-adjusted *Dunn*'s $p < 0.05$; **, BH-adjusted *Dunn*'s $p < 0.01$; ***, BH-adjusted *Dunn*'s $p < 0.001$. The color bar indicates phylum assignment of each species, orange, Actinobacteria; yellow, Firmicutes; purple, Bacteroidetes; green,

Proteobacteria; pink, Fusobacteria. A list of all species that differ significantly in abundance between the six protocols is shown in **Supplementary Table S6**.

Fig. 5 Links between country-specific gut microbial signatures and the corresponding fecal DNA extraction protocols

a, Principal coordinate analysis (PCoA) based on species-level Bray-Curtis dissimilarities between the three cohorts. Red, Chinese adults (protocol MetaHIT); blue, Danish adults (protocol MetaHIT); green, US adults (protocol PS).

b, Comparison of Bray-Curtis dissimilarities at the species level between the three cohorts. Grey, Chinese vs. US adults; brown, Danish vs. US adults; orange, Chinese vs. Danish adults.

c, Heatmap showing the significantly differed gram-negative species from Bacteroidetes, and gram-positive species from Firmicutes between Chinese, Danish and US adults. Color key indicates the mean rank of relative abundance of each species between comparisons in the Kruskal Wallis test.

d, Bacteroidetes to Firmicutes ratio (B/F ratio) between Chinese, Danish and US adults. Y-axis indicates log2 transformed values of the B/F ratio. For **b-d,** pairwise comparisons using *Dunn*'s test were followed after the Kruskal Wallis test. Asterisks as indicators for statistical significance, *, BH-adjusted *Dunn*'s $p < 0.05$; **, BH-adjusted *Dunn*'s $p < 0.01$; ***, BH-adjusted *Dunn*'s $p < 0.001$.

Additional figures and figure legends

Figure S1 Comparison of DNA yields from MMC (a) and human fecal samples (b) between protocols. Thirty-six available MMC datasets (6 datasets per protocol) and 107 available human fecal metagenomic datasets (individual B, n=36; individual D, n=36; and individual F, n=35) were used. Comparisons between protocol Q *vs.* the other protocols, and protocol PS *vs.* the other protocols are shown in this figure. Asterisks as indicators for statistical significance, *, adjusted *Dunn's* $p < 0.05$; **, adjusted *Dunn's* $p < 0.01$; ***, adjusted *Dunn's* $p < 0.001$. Detailed results of the six protocols are provided in **Supplementary Table S3.**

Figure S2 Comparison of bacterial and fungal DNA yields using different bead conditions. DNA extraction performance was assessed using three cell cultures including only *Escherichia coli* K-12 MG1655 (*E. coli* MG1655) (Bacteria, left), *Saccharomyces cerevisiae* BY4741 (*S. cerevisiae* BY4741) (Yeast, right) and a combination of 2/3 volume of *E. coli* MG1655 and 1/3 volume of *S. cerevisiae* BY4741 (Mixture, middle). Color bars indicate bead conditions based on MagPure Fast Stool DNA KF Kit B (MP); green, 500µL of Φ0.1mm beads; purple, 250µL of Φ0.1mm beads mixed with 250µL of Φ0.6~0.8mm beads; orange, 500µL of Φ0.6~0.8mm beads. For each experimental group, extractions were carried out with ten technical replicates. Wilcoxon rank-sum test, $p < 0.05$. Asterisks as indicators for statistical significance, * $p < 0.05$; ** $p < 0.01$**;** *** $p < 0.001$.

Figure S3 Gene-level intra- and inter-protocol consistency using human fecal samples for extraction and analysis. Spearman's rho (a) and Bray-Curtis dissimilarities (b) between six technical replicates within each protocol. Spearman's rho (c) and Bray-Curtis dissimilarities (d) between protocol Q and the five other protocols.

Figure S4 Comparison of gene count (a), species count (b), gene-based Shannon diversity (c), and species-based Shannon diversity (d) between protocol Q and the other five protocols. One hundred seven available human fecal metagenomic datasets (individual B, n=36; individual D, n=36; and individual F, n=35) were used. Asterisks as indicators for statistical significance, *, adjusted *Dunn*'s $p < 0.05$; **, adjusted *Dunn*'s $p < 0.01$; ***, adjusted *Dunn*'s $p < 0.001$. NS, not significant. Detailed results between 6 protocols are provided in **Supplementary Table S5.**

Figure S5 Heatmap showing gram-negative (a) and gram-positive (b) species that differ significantly in abundance between protocol PS and other protocols. Color key indicates the mean rank of relative abundance of each species between comparisons in the Kruskal Wallis test. *Dunn*'s *post hoc* tests were followed by the Kruskal Wallis test and *Dunn*'s P values were adjusted by the BH method. Asterisks as indicators for statistical significance, *, adjusted *Dunn*'s $p < 0.05$; **, adjusted *Dunn*'s $p < 0.01$; ***, adjusted *Dunn*'s $p < 0.001$. The color bar indicates phylum assignment of each species, orange, Actinobacteria; yellow, Firmicutes; purple, Bacteroidetes; green, Proteobacteria; pink, Fusobacteria. A list of all species that differ significantly in abundance between the six protocols is provided in **Supplementary Table S6.**

Figure S6 Relative abundance distributions of representative gut bacterial species at the individual level. (a), gram-positive species, (b), gram-negative species. Each point indicates the relative abundance of a given species from an individual sample. Light green, protocol Q; green, protocol MP; blue, protocol MN; purple, protocol ZYMO; orange, protocol MetaHIT; yellow, protocol PS. X-axis indicates six individuals (A to F), Y-axis indicates log2 transformed relative abundance of a given species.

Figure S7 Heatmap showing species that differ significantly in abundance between protocol PS and three Q based protocols. Comparisons were performed on 28 published metagenomic datasets (protocol PS, n=8; protocol Q-6, n=8; protocol Q-9, n=8; protocol Q-15, n=4) from a published benchmark study[11]. Color key indicates the mean rank of relative abundance of each species between comparisons in the Kruskal Wallis test. *Dunn*'s *post hoc* tests were followed by the Kruskal Wallis test. Asterisks as indicators for statistical significance, * adjusted *Dunn*'s $p < 0.05$; ** adjusted *Dunn*'s $p < 0.01$; *** adjusted *Dunn*'s $p < 0.001$. The color bar indicates phylum assignment of each species, orange, Actinobacteria; yellow, Firmicutes; purple, Bacteroidetes; green, Proteobacteria; pink, Fusobacteria and the gram staining characteristics of species, red, gram-positive; blue, gram-negative. Blue indicates species with the same enrichment direction between protocol PS and Q in the present study.

Fig.1

Click here to access/download;Figure;Fig.1.pdf

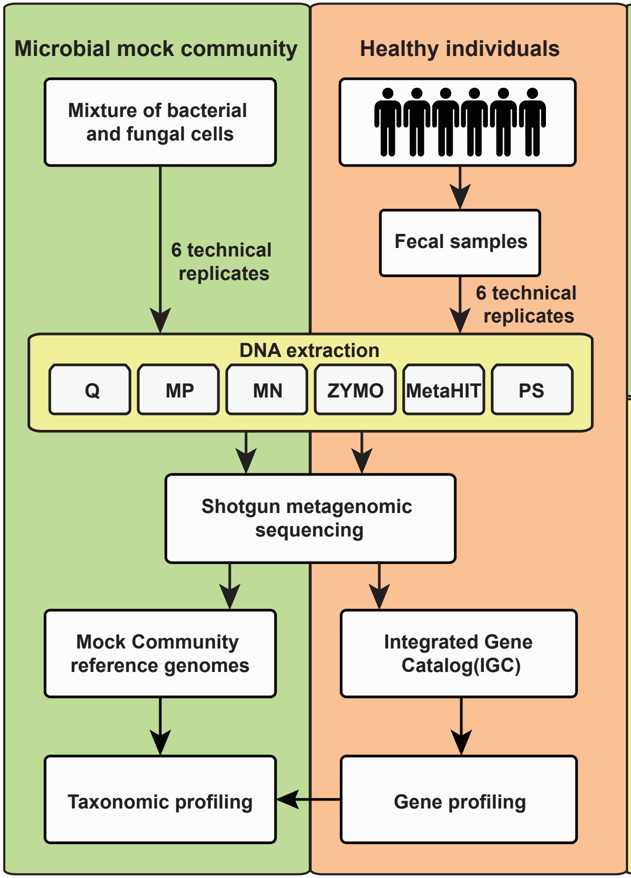

Microbial mock community

Species	Genome Size(Mb)	GC Content(%)	Gram Staining	Composition
Staphylococcus aureus	2.93	32.7	G+	12%
Enterococcus faecalis	3.01	37.5	G+	12%
Listeria monocytogenes	2.95	38	G+	12%
Bacillus subtilis	3.98	43.8	G+	12%
Lactobacillus fermentum	2.08	52.8	G+	12%
Salmonella enterica	4.83	52.2	G-	12%
Escherichia coli	5.47	56.8	G-	12%
Pseudomonas aeruginosa	6.77	66.2	G-	12%
Saccharomyces cerevisiae	13.3	38.4	Yeast	2%
Cryptococcus neoformans	18.9	48.2	Yeast	2%

DNA extraction protocols

Protocol	Abbreviation	Bead diameter
Protocol Q*	Q	0.1mm
MagPure Fast Stool DNA KF Kit B	MP	0.1mm
Macherey Nagel™ NucleoSpin™ Soil	MN	0.6-0.8mm
Zymo Research Quick-DNA™ Fecal/Soil Microbe	ZYMO	0.1 & 0.5mm
MetaHIT protocol	MetaHIT	0.1mm
MOBIO DNeasy PowerSoil	PS	Irregular beads

Fig.2

Click here to access/download;Figure;Fig.2.pdf

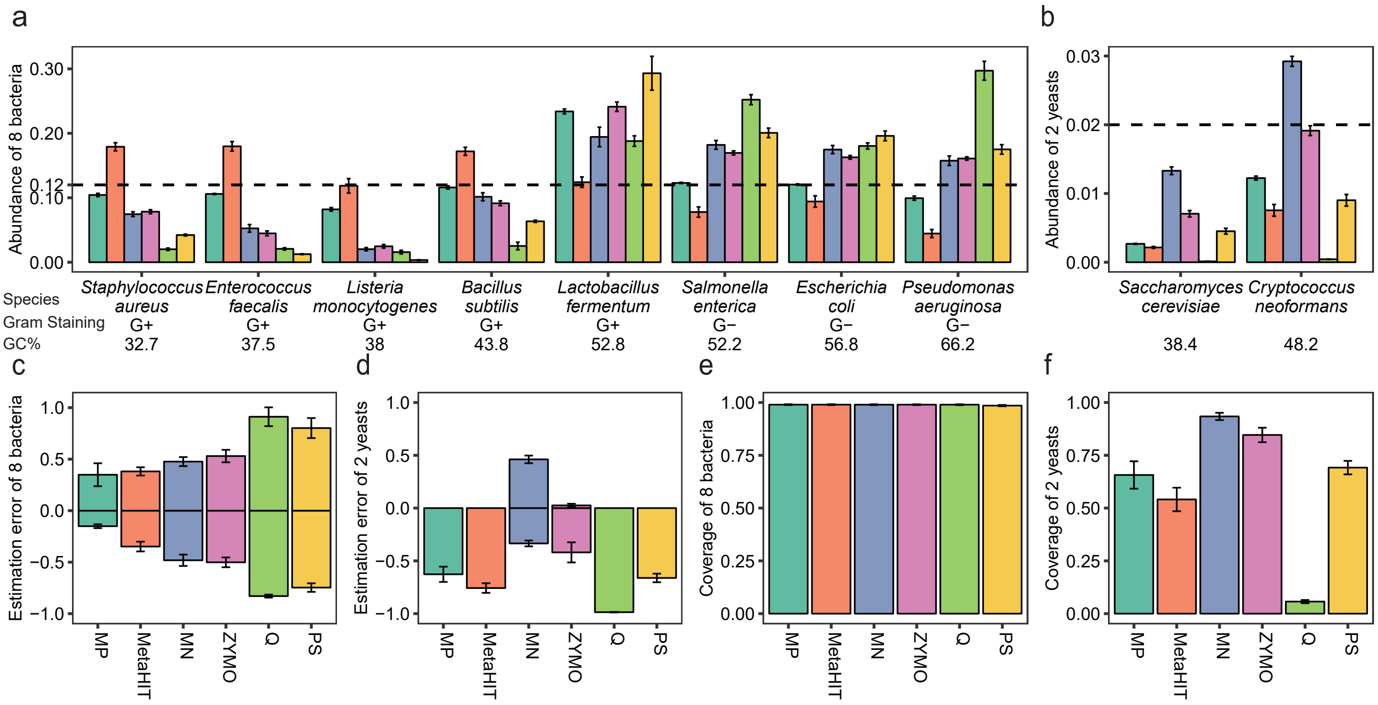

Fig.3

Click here to access/download;Figure;Fig.3.pdf

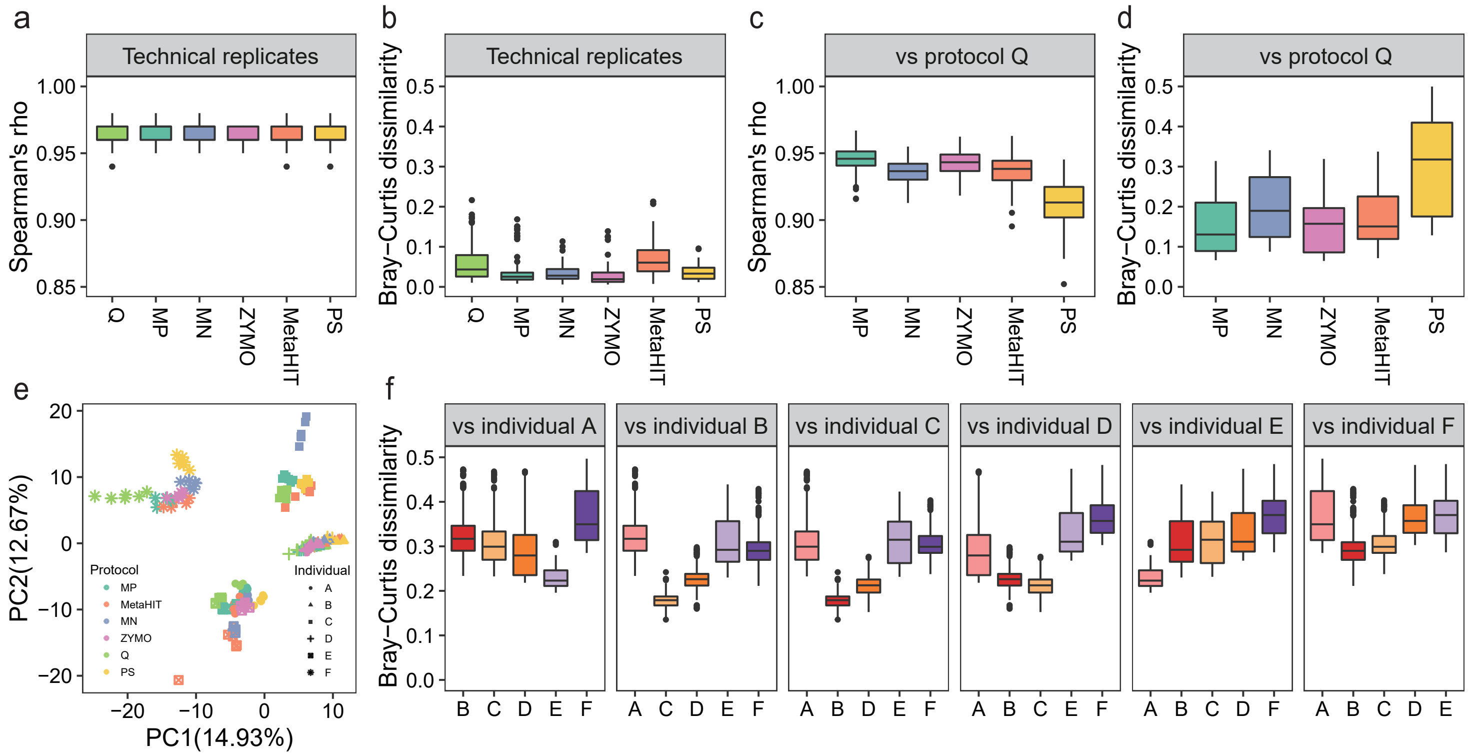

Fig.4

Click here to access/download;Figure;Fig.4.pdf

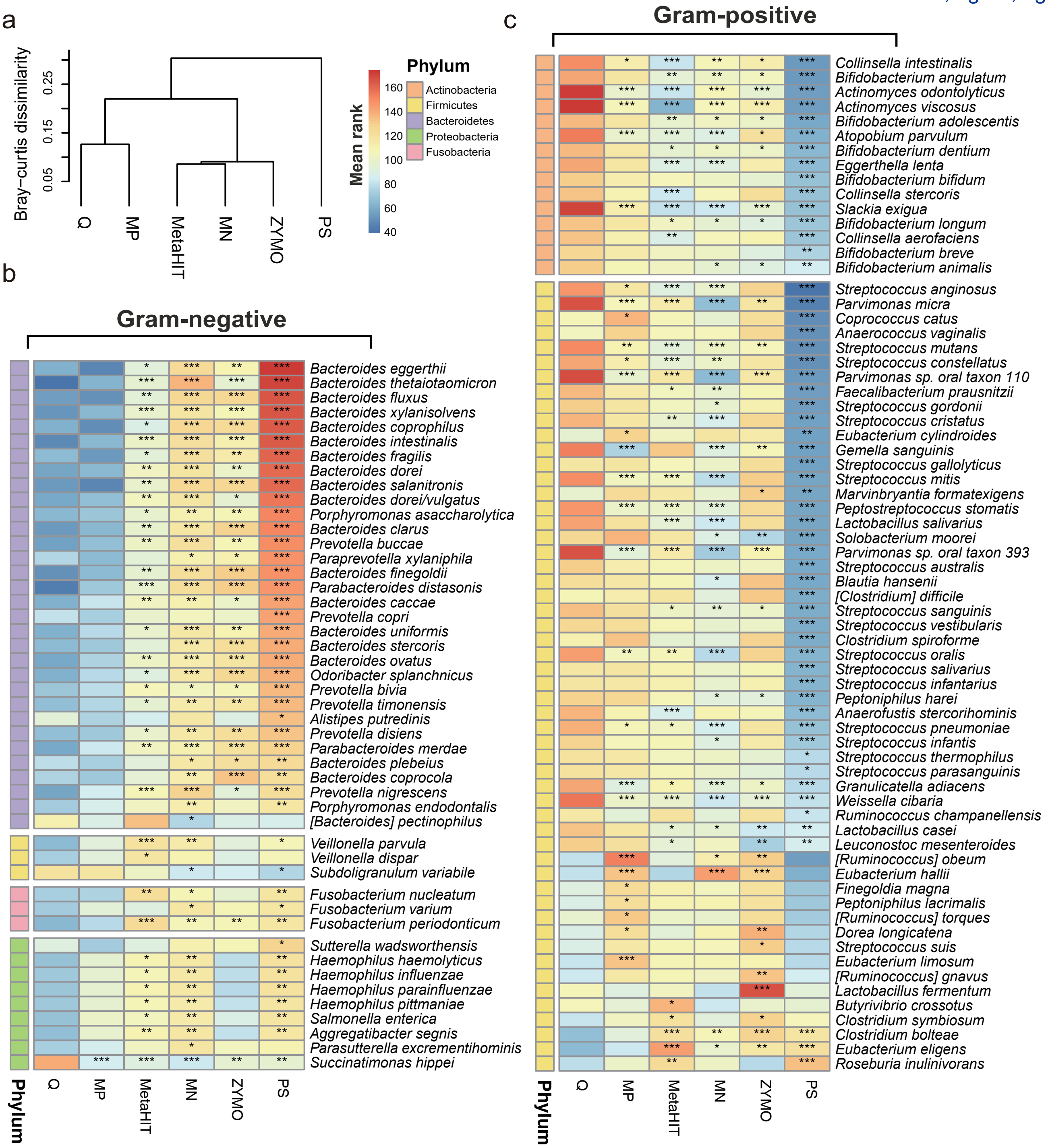

Fig.5

Click here to access/download;Figure;Fig.5.pdf

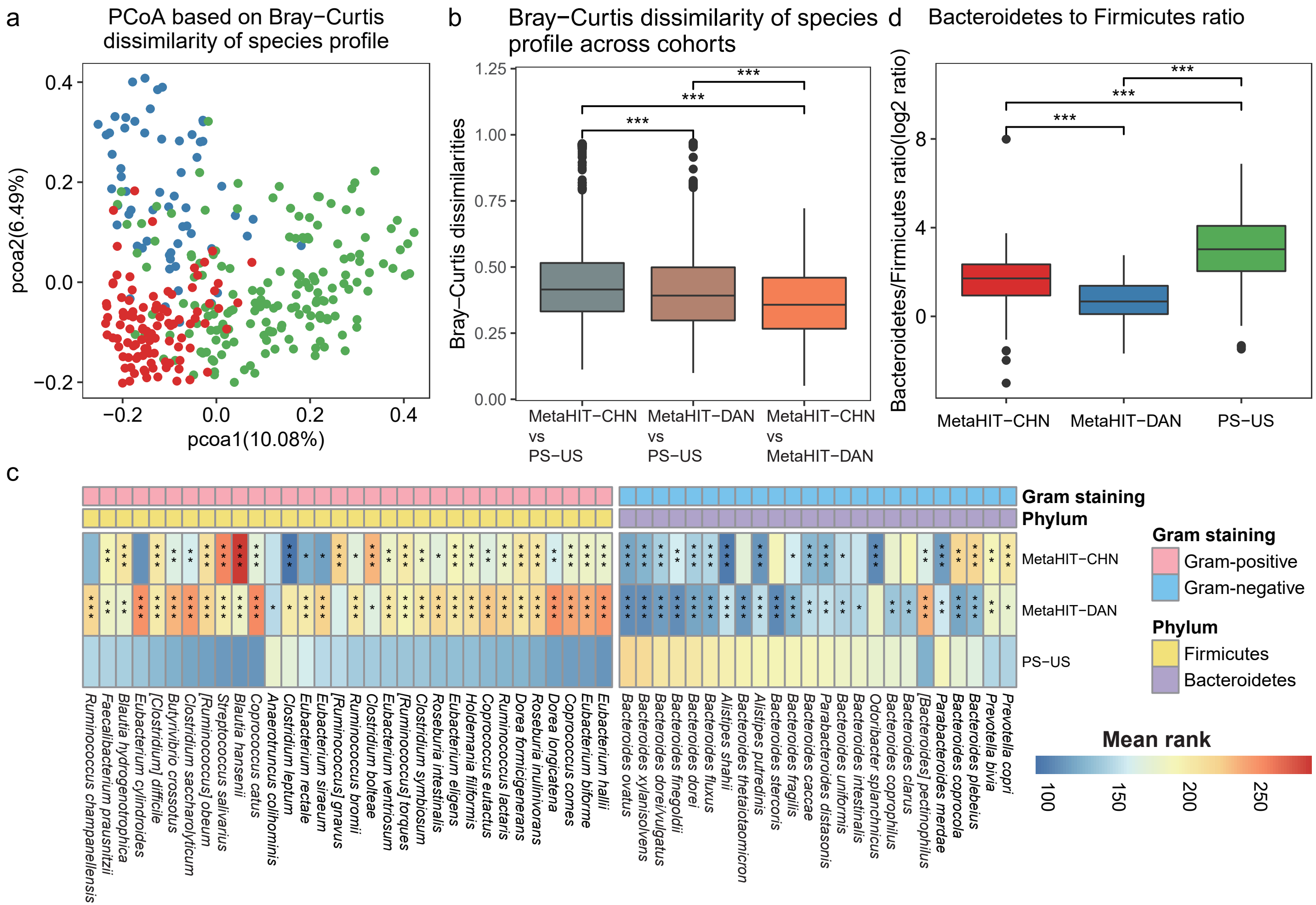

Supplementary Fig.1

Click here to access/download
Supplementary Material
Figure S1.pdf

Supplementary Fig.2

Click here to access/download
Supplementary Material
Figure S2.pdf

Supplementary Fig.3

Click here to access/download
Supplementary Material
Figure S3.pdf

Supplementary Fig.4

Click here to access/download
Supplementary Material
Figure S4.pdf

Supplementary Fig.5

Click here to access/download
Supplementary Material
Figure S5.pdf

Supplementary Fig.6

Click here to access/download
Supplementary Material
Figure S6.pdf

Supplementary Fig.7

Click here to access/download
Supplementary Material
Figure S7.pdf

Additional file

Click here to access/download
Supplementary Material
Additional file 1.docx

Supplementary Tables

Click here to access/download

Supplementary Material

Supplementary Tables S1-9.xlsx

Respond to Reviewers
Click here to access/download;Personal Cover;2020-03-24-GIGA-D-20-00011-rebuttal.docx

We appreciate the reviewers' thorough advice and comments. By the changes made in the revised manuscript and the responses provided below, we hope that we have adequately addressed the reviewers' concerns.

Reviewer reports:

Reviewer #1:

In this study, six DNA extraction protocols were compared for whole genome shotgun metagenomic sequencing using one mock community and fecal samples. From the extraction of the mock community and individual cell cultures, larger beads were found to be associated with greater fungal extraction efficiency, but lower efficiency for bacteria. The MagPure kit had the highest mean accuracy in bacterial abundance from the mock community samples, but the five protocols other than the MetaHIT proposal tended to underestimate gram-positive bacteria and overestimate gram-negative bacteria. From the DNA extraction from human fecal samples, the PowerSoil kit appeared to have higher relative abundances of multiple gram-positive species and lower abundances of gram-negative species compared to all of the other protocols. However, individuals tended to cluster together regardless of DNA extraction method in a PCoA plot. The difference between PowerSoil extracted samples and the MetaHIT protocol were highlighted using previous data of health Chinese, Danish, and US adults where the US samples extracted using the PowerSoil had increased gram-negative and decreased gram-positive levels compared to the Chinese and Danish samples extracted using the MetaHIT protocol.

This is a valuable contribution to the literature as DNA extraction protocols need to be evaluated in order to establish recommended protocols for large-scale studies. Some specific comments on the manuscript are below.

Response:

We thank the reviewer for his/her positive comments on our manuscript. However, we need to clarify that one of our findings was that the use of the PowerSoil kit appeared to produce lower relative abundances of multiple gram-positive species and higher abundances of gram-negative species compared to all of the other protocols.

Major comments:

* Line 87: I commend the authors for including the full protocol details they used in this study. However, while looking through the listed protocols, it appears that automation was not used for any of the extraction protocols. One justification of this study was that standardized protocols are needed for large-scale population studies (line 65), but large-scale population studies would never use fully manual extractions. This should be mentioned as a limitation of the study.

Response: We thank reviewer 1 for this constructive comment.

We have modified the manuscript (in abstract and methods) to clearly describe that all six extraction protocols were manually processed by the same technician in the same lab.

The main aim of our study was to evaluate DNA extraction performance between five protocols and the recently proposed standardized protocol Q [1] for human fecal samples. As demonstrated by Costea et al., the consistent performance of Q (modified based on the commercial QIAamp® DNA Stool Mini Kit) in efficiently extracting DNA of gram-positive bacteria from human fecal samples was extensively assessed and proven by 21 worldwide laboratories. However, the proposed protocol Q has many processing steps **(**~156min /per extraction, **Supplementary Table 1)**, and it can hardly be achieved automatically. On the other hand, except for the protocol Q and protocol MetaHIT, the rest four protocols we tested were all commercial kits and can be adopted in semi-automated DNA extraction systems.

In this study, we demonstrate that the MagPure kit showed highly consistent extraction performance for human fecal samples comparable to protocol Q, but required fewer processing steps and less time (~45min /per extraction, **Supplementary Table 1**). We agree with the reviewer's comment that large-scale studies would never use fully manual extractions, and we believe that our results provide useful information for further developing and improving an automated, standardized fecal DNA extraction protocol/platform. We have also modified the discussion section in our manuscript to mention the limitation that the performance of the MagPure kit on an automatic extraction system was not evaluated in this study, and further efforts are required to assess the stability and consistency between manual and automated DNA extraction using this kit.

* Line 248: It would be helpful to discuss the limitations overall of this study. For example, these results may not extend to other sample types.

Response:
We thank reviewer 1 for this constructive comment.
We agree that our main conclusions concerning the performance of different DNA extraction protocols were drawn from human fecal samples, and these findings could not be directly generalized to other sample types (such as non-human environmental samples and those with high host DNA load and very low biomass) without further detailed studies on different sample types.
This limitation has been discussed in the revised version of the manuscript.

* Line 255: Were any blank samples included for each extraction method? Was sufficient DNA recovered for sequencing?

Response:

We did not include any blank samples for DNA extraction in this study.

We are aware that, for amplicon-based studies and extraction studies on low-biomass samples, blank samples (negative controls) are necessary to assess and trace the sources of possible nucleic acid contamination introduced from multiple experimental procedures.

For most samples, we did extract sufficient DNA from both the mock microbial community (average 0.77µg per sample) and real human fecal samples (average 4.31µg per sample) for shotgun metagenomic sequencing (see details of the DNA yield per sample in **Supplementary Table 2**). Also, all metagenomic datasets generated from DNA extracts of the mock microbial community had more than 97% of the total clean reads aligned to the ten reference genomes used in the mock community (SOAP 2.22, m=0, x=1000, r=1, l=30, M=4, S, p=6, v=5, S, c=0.95; see details of the reads mapping rate per sample in **Supplementary Table 2**), suggesting few contaminations were introduced during the extractions.

* Line 265: More details about the study participants and sample collection would be helpful. For example, what proportion were women? What was the age range of adults? How was the fecal sample collected? How long did it take for the samples to be transported back to the laboratory?

Response: We apologize for the omissions in the Methods section.

We have added detailed information (sex and age) of the six participants in the revised **Supplementary Table S2**.

We have also modified the methods (in line 291-300) to clearly describe the process of collection, transport and storage of fecal samples before DNA extraction: “Six healthy volunteers including one four-year-old child and five adults (32 ± 3 years old) were recruited from BGI Europe employees or family members, Copenhagen, Denmark (See detailed information in **Supplementary Table 2**). All volunteers or the guardian consented to provide fecal samples for this study. About 10-15 grams of stool was freshly collected per participant at home by using a 50mL sterile conical tube, and copies of printed instructions were used to guide the adult volunteers or the child's legal guardian for self-collection of fecal samples. After collection, samples were stored at -20 °C and transported to the laboratory on the second day with ice packs in forty minutes. Then, each sample was diluted with 1~1.5 volumes (15 mL) of Tris-EDTA (TE, 10 mM Tris pH 8.0 and 1 mM EDTA,Thermo Fisher Scientific) buffer, homogenized and divided into 36 aliquots (500 µL per aliquot). All stool aliquots were stored at -80 °C before DNA extraction.”

* Line 278: It would be important in the results to describe the failures - it looks like all samples from specific individuals failed for the PowerSoil and Zymo extractions. If you restrict to only individuals present in all extraction methods, are your results consistent?

Response:

We thank the reviewer for this valuable suggestion.

Yes, we are aware of the extraction failure in specific individuals, as also stated in the Methods section (in line 310-313) : “Six fecal samples extracted using protocol PS (individual E) and 13 fecal samples extracted using protocol ZYMO (six of individual A, six of individual C, and one of individual F) that yielded less than 500ng and failed for library preparation, were removed from further processing.”

As all extractions on the human fecal samples by a given protocol were performed in parallel at the same time, the failure is unlikely to be caused by any laboratory procedures. However, we did not have enough fecal samples for a second-round extraction experiment on these failed samples (as a total of 36 samples per individual were used to generate six technical replicates for six different methods). On the other hand, we have successfully constructed sequencing libraries and sequenced DNA from all 36 extractions of the mock microbial community, although they had a lower microbial DNA yield as compared to the human fecal samples.

One explanation for the failure of samples from specific individuals could be that certain extraction kits (PowerSoil and Zymo) might not effectively remove complex compounds (such as humic acids, polysaccharides, bile acids and lipids, which were not contained in the mock microbial community) in fecal samples which possibly might act as PCR inhibitors to impact sequencing library construction[2].

As we show in **Figure S6** (Relative abundance distributions of representative gut bacterial species at the individual level), the PowerSoil kit yielded obviously higher relative abundances of six selected representative gram-negative species (yellow) in all five individuals than the other five extraction protocols. Consistently, the MP and protocol Q had similar low relative abundances of all gram-negative species (light green and green) in all human fecal samples. These observations at the individual level were in line with the findings based on all samples **(Figure 4)**.

To address the reviewer's concerns, we here also present the intra-individual relative abundances of 40 species in DNA samples extracted by all protocols.

In each individual, PS-extracted samples (yellow) showed consistently lower relative abundances of most gram-positive species (upper panel, **Rebuttal Figure 1a**) and higher relative abundances of most gram-negative species (lower panel, **Rebuttal Figure 1b**) than those extracted using the other protocols. Also, samples extracted by Q and MP showed consistently lower relative abundances of most gram-negative species than PS and other protocols **(Rebuttal Figure 1b)**. These observations were consistent in different individuals and were in line with our main reported findings (**Figure 4**).

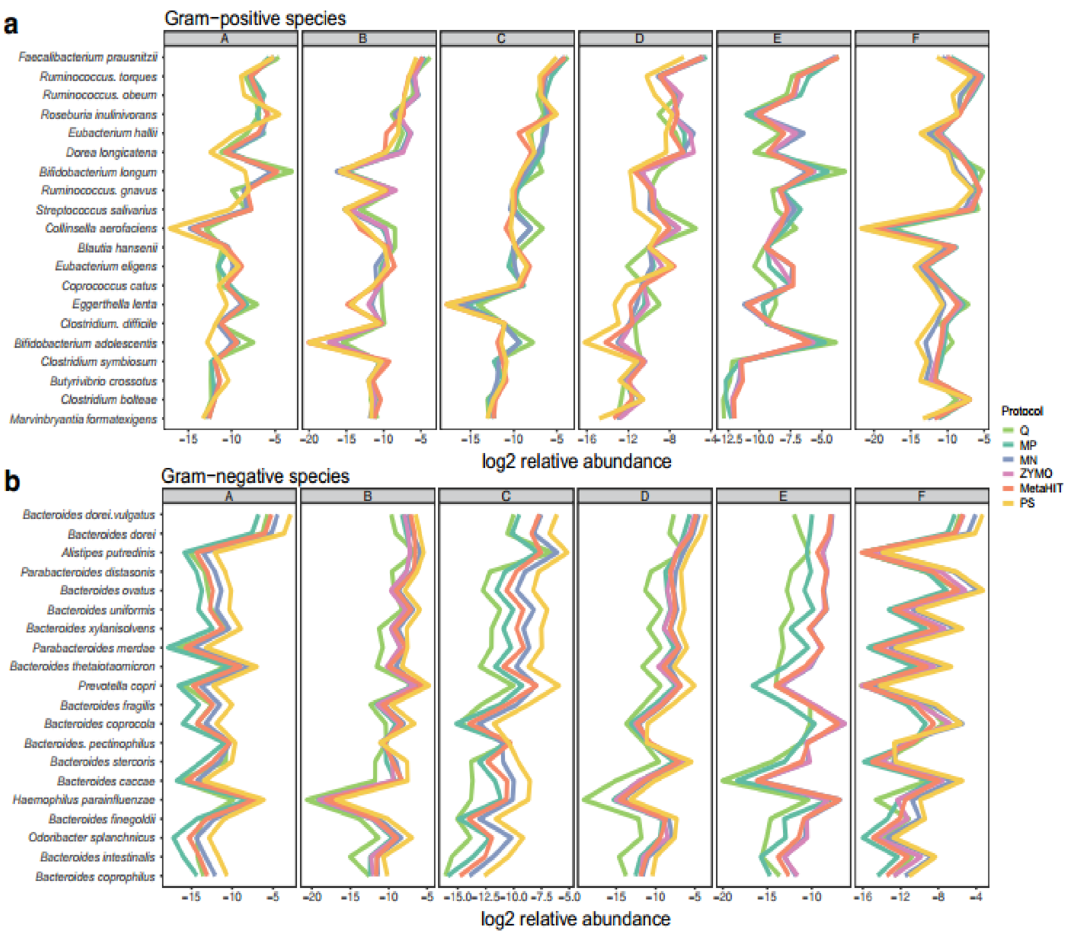

Rebuttal Figure 1 Relative abundance distributions of representative gut bacterial species within each individual.

(a), gram-positive species, (b), gram-negative species. Each line indicates the relative species abundance in the DNA sample extracted using a given extraction protocol. Light green, protocol Q; green, protocol MP; blue, protocol MN; purple, protocol ZYMO; orange, protocol MetaHIT; yellow, protocol PS. X-axis indicates log2 transformed relative abundance of a given species, only top 20 abundant gram-positive (a) or gram-negative (b) species are shown (Y axis). Each panel indicates an individual (A to F).

Minor comments:

* Line 57: Shotgun metagenomics also has its own limitations so you cannot completely ignore 16S rRNA gene sequencing.

Response: We thank reviewer 1 for this constructive suggestion.

In the revised manuscript, we have stated: “During the past two decades, PCR-based amplicon sequencing, a flexible and cost-effective method to determine microbial composition, has greatly improved our understanding of human microbiome. However, considering the known effects of PCR conditions on amplification biases such as primers, specific hypervariable regions, and annealing temperature[3,4], amplicon sequencing is insufficient for accurately evaluating

quantitative performance of bacterial DNA extraction protocols."

* Line 245: Mock communities in a matrix similar to a fecal sample would be ideal since the artificial communities do not reflect potential inhibitors and other materials found in a fecal sample.

Response:

We fully agree with the reviewer that a mock community in a matrix similar to a human fecal sample would be an ideal material to evaluate the performance of fecal DNA extraction protocols. However, due to the difficulty of culturing various kinds of anaerobic gut microbes in the laboratory, there is still no available standardized, commercial mock microbial community related to human feces. Also, as we discussed above, a mixture of microbial communities could hardly mimic the complex mixture of compounds in real fecal samples, which might inhibit the activities of enzymes for PCR-based library construction.

In the revised manuscript, we have extended the limitations of the microbial mock community in line 262-266: "In addition, the mock communities from both studies were both composed of human pathogenic bacteria or bacteria isolated from a non-human environment, which do not reflect the human gut microbial composition. Furthermore, such simple mixtures of bacteria and fungi do not contain other compounds in feces such as humic acids, polysaccharides, bile acids and lipids, which might potentially inhibit the activity of enzymes used for subsequent PCR-based library construction and sequencing[2]."

Reviewer #2:

The manuscript titled "Assessment of fecal DNA extraction protocols for metagenomics studies" by Yang et al. describe the higher efficacy of MP method for the fecal DNA extraction procedure. This study also compares validity and reproducibility of six different DNA extraction method with mock and human fecal samples. As mentioned by the authors, standardized and robust DNA extraction protocol is still needed for the comparison between globally produced gut microbiome data. In addition, I agree with the necessity of new analytical methods for comprehensive and accurate understand of gut mycobiome as well. In that respect, I think your manuscript is timely necessary and important. However, there are some point might be considered in revision.

The most important point is that the microorganisms contained in currently used mock community are not abundant members of human gut microbiome. In the recent study by Sunagawa et al., as referred in your manuscript, the authors construct a mock community considering human gut microbial composition. While MP showed higher mean accuracy in bacterial abundance estimation than other protocol and Q showed lowest recovery of the two yeast genomes in mock sample analysis. Microbiome extracted with Q protocol still have distinct community composition compared to the other method, even with MP protocol, especially in G+ bacteria. So, I recommend the authors check where this discrepancy come from with the other mock samples or manually constructed human microbiome mock samples.

Response: We thank reviewer 2 for this constructive comment.

In the current study, we used a commercial mock community (ZymoBIOMICS Microbial Community Standard, Catalog No. D6300) containing cells of eight bacteria (each making up 12%) and two yeast strains (each contributing 2%). All these species are human pathogens or isolated from a non-human environment, facultative anaerobes (easy to be cultured), and are not high-abundant residents in the human gut.

Similarly, the benchmark study (Sunagawa et al, metioned by the reviewer should be Costea et al, 10.1038/nbt.3960) also used a mock community containing 10 bacterial species that were generally absent from the healthy gut microbiota, including *Prevotella melaninogenica* (G-), *Clostridium perfringens* (G+), *Salmonella enterica* (G-, also used in the current study), *Clostridium difficile* (G+), *Lactobacillus plantarum* (G+), *Clostridium saccharolyticum* (G+), *Yersinia pseudotuberculosis* (G-), *Vibrio cholerae* (G-), *Blautia hansenii* (G+) and *Fusobacterium nucleatum* (G-)(Costea et al, 10.1038/nbt.3960, **Figure 6**). Thus, both studies did not use representative and high-abundant gut microbes for the mock materials.

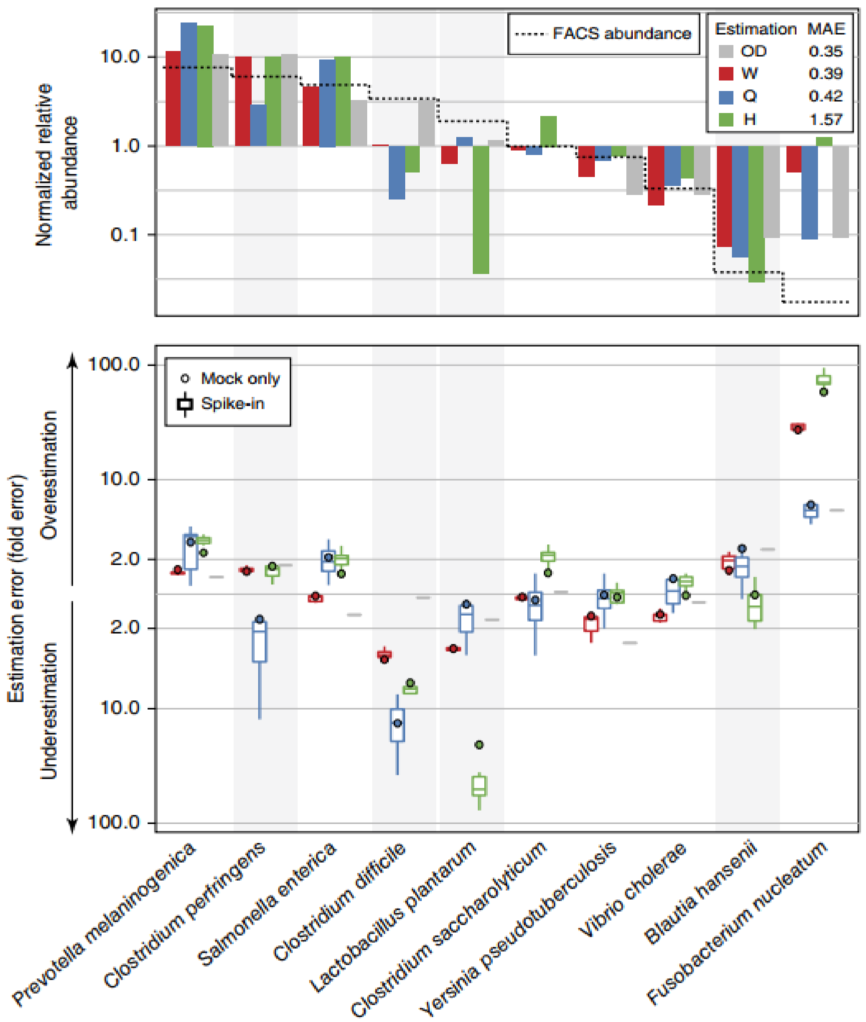

(**Figure 6**, Costea et al, 10.1038/nbt.3960)

We are aware that the assessment of mock microbial community might not fully reflect the extraction performance in real human fecal samples. Also, we did observe the inconsistency of the extraction efficiency of gram-positive bacteria between the mock community and fecal samples. Except for the MetaHIT protocol, all other five protocols underestimated the abundance of four among the five gram-positive bacteria in the mock (*Staphylococcus aureus*, *Enterococcus faecalis*, *Listeria monocytogenes* and *Bacillus subtilis*) but overestimated the abundance of gram-positive *Lactobacillus fermentum* (**Figure 2**). By contrast, four protocols (MN, ZYMP, Q and PS) overestimated the abundance of all three gram-negative bacteria (*Salmonella enterica*, *Escherichia coli* and *Pseudomonas aeruginosa*) (**Figure 2**). As reported by Costea et al (10.1038/nbt.3960, **Figure 6**), regardless of extracting DNA from the mock itself or from fecal sample with a spike-in mock community, protocol Q (blue) underestimated the abundance of gram-positive bacteria including *C. perfringens*, *C. difficile* and *L. plantarum* and overestimated the abundance of three gram-negative members including *P. melaninogenica*, *S. enterica and F. nucleatum*. My suggestion: regardless of whether DNA was extracted from a mock community or from a fecal sample with a spike-in

mock community, protocol Q underestimated the abundances of gram-positive bacteria including *Clostridium perfringens*, *C. difficile* and *Lactobacillus plantarum* and overestimated the abundance of three gram-negative members including *S. enterica*, *Prevotella melaninogenica* and *Fusobacterium nucleatum*. Thus, the observations based on mock communities in the two studies were somehow consistent, suggesting overall different efficiencies of obtaining whole-genome DNA from gram-positive and gram-negative bacteria, as well as variable efficiencies between different gram-positive species.
We note the discrepancy between the DNA extraction performance on mock communities and fecal samples in our study as well as the benchmark study (Costea et al). Both studies have demonstrated that the fecal DNA samples extracted by protocol Q displayed higher relative abundances of multiple gram-positive species than other methods.

As we note in our response to the Reviewer 1, so far, it is still challenging to create a mock microbial community that can mimic human feces. The two reasons are that 1) most of the gut residents are anaerobic and hard to culture and 2) a simple mixture of microbial species will not reflect the complex, highly variable chemical and physical properties of human feces, which might potentially impact the activities of enzymes for downstream library construction and sequencing. Thus, extraction performance based on an MMC will not precisely and unbiasedly reflect extraction performance on human fecal samples.

Also, for both studies, quantitative performance on extracting human gut microbiome between protocols has been interpreted based on bacterial relative abundance but not absolute abundance, which we measured in the mock. Further efforts are still needed to quantify absolute microbial abundances in fecal mock materials with a mixture of both abundant gut microbes and non-living fecal compounds, and in real fecal samples to accurately assess the quantification biases of different protocols.

We have extensively revised our manuscript to discuss the limitations of our study as well as previous ones (in line 251-270), and we hope we have addressed the reviewer's concerns about the discrepancy of the DNA extraction performance on mock communities and fecal samples.

2. As described by the authors, very low levels of mycobiome only in few fecal samples were detected with tested protocols and fungal sequence reads were only identified in one sample both MP and Q protocols. Therefore, I am not sure we can determine that MP is a more effective method for human metagenomic analysis than Q protocol, even though MP showed greater efficacy in mock sample analysis than Q protocol.

Response:
First, a measurable fungal abundance was only detected in few samples in this study. However, these observations do not necessarily imply that there were no fungal

genomes extracted in human feces by the six protocols.
Previous studies have demonstrated very low levels of fungi in human fecal samples[5–7]. As reported by Richard et al, the number of fungi in faces has been shown to be far lower than that of bacteria, with 10^5 to 10^6 fungal cells per gram of fecal matter compared with 10^{11} bacterial cells per gram [7]. In addition, the genome sizes of fungi are also much greater than that of bacteria. Thus, a much greater amount of sequencing data than we generated in the current study is needed to evaluate the performance of fecal mycobiome extraction across protocols. Amplicon-based approaches (18S rRNA-based or ITS-based) seem still to be more cost-effective and appropriate in order to assess the mycobiome in human fecal samples, and such amplicon-based approaches have been successfully applied in several studies[8–10].

Second, our study has shown a positive correlation between the bead size and extraction efficiency of yeast DNA **(Figure 2d,f and Supplementary Figure 2**), and the two protocols (MN using 0.6-0.8mm beads, and ZYMO using a mixture of 0.1&0.5 beads) have shown better performance in recovering genome coverage and abundance of the two yeast strains than other three protocols using 0.1mm beads (MetaHIT, MP and Q). We concur that a limitation of our study is that we did not apply amplicon-based sequencing to assess whether the performance of extracting fungi DNA using the different protocols is the same using mock or fecal samples.

We suggest researchers to consider using protocols with larger beads when their research interests are mainly focused on the mycobiome. Due to the limited sequencing depth of mycobiome from our shotgun metagenomic datasets, we were not able to compare the extracting performance of mycobiome between the MP and Q, or between any two protocols. We have modified our results (in line 188-189 and discussions (in line 243-250) to clearly describe our findings to help the reviewers and readers better understand our results.

References

1. Costea PI, Zeller G, Sunagawa S, Pelletier E, Alberti A, Levenez F, et al. Towards standards for human fecal sample processing in metagenomic studies. Nature Biotechnology [Internet]. Nature Publishing Group; 2017;35:1069–76. Available from: http://dx.doi.org/10.1038/nbt.3960

2. Schrader C, Schielke A, Ellerbroek L, Johne R. PCR inhibitors - occurrence, properties and removal. Journal of Applied Microbiology. 2012;113:1014–26.

3. Orpana AK, Ho TH, Stenman J. Multiple heat pulses during PCR extension enabling amplification of GC-rich sequences and reducing amplification bias. Analytical Chemistry. 2012;

4. Laursen MF, Dalgaard MD, Bahl MI. Genomic GC-content affects the accuracy of 16S rRNA gene sequencing bsed microbial profiling due to PCR bias. Frontiers in Microbiology. 2017;

5. Huffnagle GB, Noverr MC. The emerging world of the fungal microbiome. Trends in Microbiology. 2013.

6. Sam QH, Chang MW, Chai LYA. The fungal mycobiome and its interaction with gut bacteria in the host. International Journal of Molecular Sciences. 2017.

7. Richard ML, Sokol H. The gut mycobiota: insights into analysis, environmental interactions and role in gastrointestinal diseases. Nature Reviews Gastroenterology and Hepatology [Internet]. Springer US; 2019;16:331–45. Available from: http://dx.doi.org/10.1038/s41575-019-0121-2

8. Frau A, Kenny JG, Lenzi L, Campbell BJ, Ijaz UZ, Duckworth CA, et al. DNA extraction and amplicon production strategies deeply influence the outcome of gut mycobiome studies. Scientific Reports [Internet]. Springer US; 2019;9:1–17. Available from: http://dx.doi.org/10.1038/s41598-019-44974-x

9. Nash AK, Auchtung TA, Wong MC, Smith DP, Gesell JR, Ross MC, et al. The gut mycobiome of the Human Microbiome Project healthy cohort. Microbiome. 2017;

10. Zuo T, Wong SH, Cheung CP, Lam K, Lui R, Cheung K, et al. Gut fungal dysbiosis correlates with reduced efficacy of fecal microbiota transplantation in Clostridium difficile infection. Nature Communications. 2018;

Personal Cover
Click here to access/download;Personal Cover;2020-03-24-GIGA-D-20-00011-Coverletter.docx

Dear Editor,

On behalf of all the authors, we hereby submit a revised version of our manuscript entitled: **"*Assessment of fecal DNA extraction protocols for metagenomic studies*".**

We are thankful for the constructive suggestions made by the reviewers. We have accordingly discussed the limitations of the study in the revised manuscript and in the rebuttal letter, regarding i) the DNA extraction failures of human fecal samples, ii) the inconsistent quantitative performance of gram-negative and gram-positive bacteria between extractions on mock communities and human feces, and iii) the lack of validated performance of DNA extraction protocols for fungal communities in human feces. All revised texts are highlighted in blue. We hope that the manuscript after this revision has adequately addressed the reviewers' concerns and could be acceptable for publication in ***GigaScience***.

Yours sincerely,

Huanzi Zhong
