# Supplementary material for: Assessment of fecal DNA extraction protocols for metagenomic studies
Source: Gigascience. 2020 Jul 13;9(7):giaa071. doi: 10.1093/gigascience/giaa071 (PMC7355182; doi:10.1093/gigascience/giaa071)
Supplement: giaa071_Supplemental_Files [file giaa071_supplemental_files.zip › Additional File 1-update.docx]

# DNA extraction protocol Q (Q)[1]

Modified after the QIAamp® DNA Stool Mini Kit Catalog No. 51504

**1^a^.** Homogenize 150 to 200 mg frozen fecal material with 1.0 mL ASL lysis buffer of the kit by vortexing (Vortexer, VWR Mixer mini Vortex (230V EU) Model NO: 945304) for 2 min in a 2 mL tube containing 0.3 g of 0.1 mm glass beads.

**2.** Incubate for 15 min at 95°C.

**3^b^.** Vortex 8 times at maximum speed using the following scheme: vortexing 1 min, resting 5 min.

**4.** Samples are allowed to cool down on ice for 2 min.

**5.** Samples are centrifuged at 16,000 x *g*, 4°C, for 5 min.

**6.** Supernatant is transferred to a new 2 mL tube.

**7.** The pellet is mixed with 300 µL ASL lysis buffer of the kit, and steps 2-5 are repeated.

**8.** Supernatants are pooled in the new 2 mL tube.

**9.** Add 260 µL of 10M ammonium acetate to each lysate tube, mix well, and incubate on ice for 5 min.

**10.** Centrifuge at 16,000 x *g*, 4°C, for 10 min.

**11.** Transfer the supernatant to two 1.5 mL Eppendorf tubes, add one volume of isopropanol, mix well, and incubate on ice for 30 min.

**12.** Centrifuge at 16,000 x *g*, 4°C, 15 min, remove the supernatant using aspiration, wash nucleic acids pellet with 70 % EtOH (0.5 mL) and dry the pellet under vacuum for 3 min.

**13.** Dissolve the nucleic acid pellet in 100 µL of TE (Tris-EDTA) buffer and pool the two aliquots.

**14.** Add 2 µL of DNase-free RNase (10 mg/mL) and incubate at 37°C, 15 min.

**15.** Add 15 µL proteinase K and 200 µL AL buffer to the supernatant, vortex for 15 sec and incubate at 70°C for 10 min.

**16.** Add 200 µL of ethanol (96-100%) to the lysate and mix by vortexing.

**17.** Transfer to a QIAamp spin column and centrifuge at 16,000 x *g* for 1 min, at room temperature (RT).

**18.** Discard flow through, add 500 µL buffer AW1 (Qiagen) and centrifuge at 16,000 x *g* for 1min, at RT.

**19.** Discard flow through, add 500 µL buffer AW2 (Qiagen) and centrifuge at 16,000 x *g* for 1min, at RT

**20.** Dry the column by centrifugation at RT for 1 min.

**21.** Add 200 µL Buffer AE (Qiagen), incubate for 1 min at RT

**22.** Centrifuge for 1 min at 16,000 x *g* to elute DNA.

**Comments:**

**1^a^,** In the benchmark study[1], 0.1 mm sterile zirconia beads (BioSpec, Cat. No. 11079101z) were used for bead beating in the protocol Q, here we used 0.1 mm glass disruptor beads (USA Scientific, Item No.7400-2401).

**3^b^,** In the benchmark study[1], cells were mechanically lyzed by running the Fastprep™ instrument for 8 min 15 sec (series of beating 1 min and resting 5 min), here we used vortexer (VWR Mixer mini Vortex (230V EU) Model NO: 945304).

# MagPure Fast Stool DNA KF Kit B (MP, Catalog No. MD5115-02B)

**1. Sampling:**

Mix the sample well, then add 150-200 mg of human fecal material to a 2 mL falcon tube.

**2. Enrichment**

Centrifuge the 2 mL tube at 20,000 x *g* for 5 min, at 4°C.

Remove the supernatant and keep the sediment.

**3. Shake with glass beads**

Add 0.6 mL of Buffer ATL（PVP-10）, 0.6 mL of Buffer PCI, 300 μL of glass beads (0.1 mm) and a steel ball to the sediment.

Vortex at maximum speed for 15 min.

**4. Cell lysis**

Incubate the tube at 65°C for 20 min, and then centrifuge at 20,000 x *g* for 10 min.

**5. Removal of RNase**

Transfer 400 μL of supernatant to a clean 1.5 mL tube, add 5 μL RNase Solution.

Vortex to mix well and incubate at RT for 15 min.

**6. DNA extraction**

Add 600 μL Buffer MLE, 20 μL Proteinase K, 20 μL MagPure Particles to the tube of step 5 and incubate at RT for 10 min. Vortex 3-5 times to resuspend beads during the incubation.

Place the tube onto a Magnetic frame for 5 min. Discard the supernatant.

Add 700 μL Buffer AW1, Vortex for 30 sec.

Place the tube onto a Magnetic frame for 3min. Discard the supernatant.

Add 700 μL 70% Ethanol, Vortex for 60 sec.

Place the tube onto a Magnetic frame for 3min. Discard the supernatant.

Add 700 μL 70% Ethanol, Vortex for 60 sec.

Place the tube onto a Magnetic frame for 3 min. Discard the supernatant. Dry at RT for 5-7 min.

Load 100 μL Buffer AE into the tube, mix well and incubate at 55°C for 10-15 min with gentle shaking at 300 rpm using a thermomixer.

Place the tube onto a Magnetic frame for 3 min. Transfer 100 μL supernatant to a clean 1.5 mL tube.

# Macherey Nagel™ NucleoSpin™®Soil Kit (MN, Catalog No. 740780.250)

**1. Prepare sample**

Transfer 250–500 mg of human fecal material to a NucleoSpin® Bead Tube Type A.

Add 700 μL Buffer SL1.

**2. Adjust lysis condition**

Add 150 μL Enhancer SX.

Close the NucleoSpin® Bead Tube and shake horizontally for 2–3 sec to mix stool sample and lysis buffer.

**3. Sample lysis**

Attach the NucleoSpin® Bead Tubes horizontally to a vortexer, for example, by taping or using a special adapter (MN Bead Tube Holder, REF: 740469).

Vortex the samples at 70% speed for 10 min at RT and incubate at 65°C for 10 min with gentle shaking at 1000 rpm in a thermomixer.

**4. Precipitate contaminants**

Centrifuge for 2 min at 11,000 x *g* to eliminate the foam caused by the detergent.

Transfer 600 μL of the supernatant to a fresh 2 mL microcentrifuge tube with lid.

Add 150 μL Buffer SL3, close the lid and vortex for 5 sec.

Incubate for 5 min at 0–4°C.

Centrifuge for 4 min at 20,000 x *g*.

**5. Filter lysates**

Place a NucleoSpin® Inhibitor Removal Column (red ring) in a Collection Tube (2 mL, lid).

Load up to 700 μL clear supernatant of step 4 onto the filter.

Centrifuge for 1 min at 11,000 x *g*.

Discard the NucleoSpin® Inhibitor Removal Column.

**6. Adjust binding conditions**

Add 250 μL Buffer SB and close the lid. Vortex for 5 sec.

**7. Bind DNA**

Place a NucleoSpin® DNA Soil Column (green ring) in a Collection Tube (2 mL).

Load 550 μL sample onto the column.

Centrifuge for 1 min at 11,000 x *g*.

Discard flow-through and place the column back into the collection tube.

Load remaining sample onto the column.

Centrifuge for 1 min at 11,000 x *g*.

Discard flow-through and place the column back into the collection tube.

**8. Wash and dry silica membrane**

**1st wash**

Add 500 μL Buffer SB to the NucleoSpin® Soil Column.

Centrifuge for 30 sec at 11,000 x *g*.

Discard flow-through and place the column back into the collection tube.

**2nd wash**

Add 550 μL Buffer SW1 to the NucleoSpin® Soil Column.

Centrifuge for 30 sec at 11,000 x *g*.

Discard flow-through and place the column back into the collection tube.

**3rd wash**

Add 700 μL Buffer SW2 to the NucleoSpin® Soil Column.

Close the lid and vortex for 2 sec. Centrifuge for 30 sec at 11,000 x *g*.

Discard flow-through and place the column back into the collection tube.

**4th wash**

Add 700 μL Buffer SW2 to the NucleoSpin® Soil Column.

Close the lid and vortex for 2 sec. Centrifuge for 30 sec at 11,000 x *g*.

Discard flow-through and place the column back into the collection tube.

**9. Dry silica** **membranes**

Centrifuge for 2 min at 11,000 x *g*.

**10. Elute DNA**

Place the NucleoSpin® Soil Column into a new 1.5 mL microcentrifuge tube.

Add 100 μL Buffer SE to the column.

Do not close the lid and incubate for 1 min at RT (18-25 °C).

Close the lid and centrifuge for 30 sec at 11,000 x *g*.

# Quick-DNA™ Fecal/Soil Microbe (ZYMO, Catalog No. D6010)

**1.** Add 200 mg of fecal sample to a ZR Bashing Bead™ Lysis Tube (0.1 & 0.5 mm). Add 750 µL Bashing Bead™ Buffer to the tube.

**2.** Attach a 2 mL tube holder to a vortexer and vortex 10 min at maximum speed.

**3.** Centrifuge the ZR Bashing Bead™ Lysis Tube in a microcentrifuge at ≥ 10,000 x *g* for 1 min.

**4.** Transfer up to 400 µL supernatant to a Zymo-Spin™ III-F Filter in a Collection Tube and centrifuge at 8,000 x *g* for 1 min.

**5.** Add 1,200 µL of Genomic Lysis Buffer to the filtrate in the Collection Tube from Step 4.

**6.** Transfer 800 µL of the mixture from Step 5 to a Zymo-Spin™ IIC Column4 in a Collection Tube and centrifuge at 10,000 x *g* for 1 min.

**7.** Discard the flow through from the Collection Tube and repeat Step 6.

**8.** Add 200 µL DNA Pre-Wash Buffer to the Zymo-Spin™ IIC Column in a new Collection Tube and centrifuge at 10,000 x *g* for 1 min.

**9.** Add 500 µL g-DNA Wash Buffer to the Zymo-Spin™ IIC Column and centrifuge at 10,000 x *g* for 1 min.

**10.** Transfer the Zymo-Spin™ IIC Column to a clean 1.5 mL microcentrifuge tube and add 100 µL (50 µL minimum) DNA Elution Buffer directly to the column matrix. Centrifuge at 10,000 x *g* for 30 sec to elute the DNA.

**11.** Place a Zymo-Spin™ III-HRC Filter to a clean Collection Tube and add 600 µL Prep Solution. Centrifuge at 8,000 x *g* for 3 min.

**12.** Transfer the eluted DNA to a prepared Zymo-Spin™ III-HRC Filter in a clean 1.5 mL microcentrifuge tube and centrifuge at exactly 16, 000 x *g* for 3 min.

# MetaHIT protocol [2]

A non-kit-based manual protocol developed by the International Human Microbiome Consortium (IHMC) and adopted by MetaHIT (METAgenomics of the Human Intestinal Tract consortium).

1. **Preparation of solutions**
2. **4M** **guanidine thiocyanate**
3. Formulation:

----weigh 12.37 g guanidine thiocyanate (118.2 g/mol) in a flacon tube in a fume hood

----add 13.5 mL water and 2.6 mL Tris-HCl (1M, pH 7.5-8)

----shake on a rocking agitator, and keep out of the light

----complete with water to a 26.1 mL solution

----heat in a water bath at 60-70°C for 10 min

1. Storage: 4°C, and keep out of light
2. **10% N-lauroylsarcosine**
3. Formulation:

----weigh 2 g N-lauroylsarcosine

----complete with water to a 20 mL solution, filter after all material is dissolved

1. Storage: RT
2. **5% N-lauroylsarcosine**
3. Formulation:

----weigh 1 g N-lauroylsarcosine

----complete with water to a 20 mL solution, filter after all material is dissolved

1. Storage: RT
2. **PBS**
3. Formulation:

----14.2% Na_2_HPO_4_: weigh 14.2 g solid, then complete with water to a 100 mL solution (dissolve on heating stirrer)

----12% NaH_2_PO_4_: weigh 12 g solid, then complete with water to a 100 mL solution (dissolve on heating stirrer)

----add 9.32 mL of 14.2% Na_2_HPO_4_, 0.68 mL of 12% NaH_2_PO_4_ and water to a 100 mL solution, check pH≈8 with pH test strip

1. Storage: RT
2. **TENP**
3. Formulation:

----add 1.5 mL of Tris-HCl, 1.2 mL of EDTA, 0.6 mL of NaCl and 300 mg of PVPP with water to 30 mL solution, mixing upside down.

1. Storage: RT
2. **Reagent sterilization**

High temperature sterilizing is needed for 10% N-lauroylsarcosine, 5% N-lauroylsarcosine, and PBS.

1. **Preheating the water bath at 70°C.**
2. **Procedures**

**1. Preparation of the sample**

Transfer 150–200 mg of human fecal material to a 2 mL falcon tube, add 1 mL of PBS and mix well.

**2. Enrichment**

Centrifuge the 2 mL tube at 11,000 x *g* for 5 min at 4°C.

Remove the supernatant.

**3. Cell lysis**

Add 250 μL of Guanidine thiocyanate and 40 μL of 10% N-lauroyl sarcosine, then crush the feces with a sterile toothpick.

Add 500 μL of 5% N-lauroyl sarcosine. Vortex to mix well, then centrifuge for a few seconds. Incubate at 70°C for 1 h.

**4. Shaking with glass beads**

After incubation, add 500 μL of 0.1 mm glass beads and 500 μL of TENP to the tube. Vortex to dissolve the pellet.

Vortex at maximum speed for 15 min.

Centrifuge at 11,000 x *g* for 15 min.

**5. Isopropanol precipitation**

Transfer the supernatant (≈900 μL) to a new 2 mL falcon tube.

Add 900 μL of isopropanol, gently mix well by inversion.

Incubate at -20°C for 1 h.

**6. Resuspending**

Centrifuge at 11,000 x *g* for 15 min, and then remove the supernatant.

Add 450 μL of PBS and 50 μL of potassium acetate, using the pipette to dissolve the pellet.

Vortex to mix well, then incubate at 4°C for 1 h.

**7. 100% Ethanol precipitation**

Centrifuge the tube at 11,000 x *g* in 4°C for 20 min.

Transfer 500 μL of the supernatant to a new 1.5 mL falcon tube, then add 1 mL of 100% ethanol. Vortex to mix well, incubate at -20°C for 1 h.

Centrifuge the tube at 11,000 x *g* at 4°C for 15 min.

**8. Washing the sediment**

Add 1 mL of 75% ethanol to the sediment, and vortex to mix well.

Centrifuge at 11,000 x *g* in 4°C for 5 min, then remove the supernatant.

Centrifuge for a few seconds, and then using the pipette to remove the residual supernatant.

Incubate at RT for 3-5 min.

**9. Dissolution and RNA digestion**

Add 50-100 μL of TE buffer according to the amount of sediment and 2 μL of RNase A, then vortex to mix well. Centrifuge at 1000 x *g* for 3 seconds.

Incubate in a water bath at 37°C for 30 min. During the incubation, flip the tube every 10 min to promote the dissolution of the sediment and RNA digestion.

**10. The extracted DNA should be stored at -20°C after incubation.**

# MOBIO DNeasy PowerSoil (PS, Catalog No. 12888-100)

**Procedure**

**1.** Add 250 mg of fecal sample to the PowerBead Tube provided. Gently vortex to mix.

**2.** Add 60 µL of Solution C1 and invert several times or vortex briefly.

**3.** Secure PowerBead Tubes horizontally using a Vortex Adaptor for 12 tubes (MN Bead Tube Holder, REF: 740469).

**4.** Vortex at maximum speed for 15 min.

**5.** Centrifuge tubes at 10,000 x *g* for 30 sec .

**6.** Transfer the supernatant to a clean 2 mL Collection Tube.

**7.** Add 250 µL of solution C2 and vortex for 5 sec. Incubate at 2-8°C for 5 min.

**8.** Centrifuge the tubes for 1 min at 10,000 x *g*.

**9.** Avoiding the pellet, transfer up to 600 µL of the supernatant to a clean 2 mL Collection Tube.

**10.** Add 200 µL of solution C3 and vortex briefly. Incubate at 2-8°C for 5 min.

**11.** Centrifuge the tubes for 1 min at 10,000 x *g.*

**12.** Avoiding the pellet, transfer up to 750 µL of the supernatant to a clean 2 mL Collection Tube.

**13.** Shake to mix Solution C4 and add 1200 µL to the supernatant. Vortex for 5 sec.

**14.** Load 675 µL onto an MB Spin Column and centrifuge at 10,000 x *g* for 1 min. Discard flow-through.

**15.** Repeat step 14 twice, until all of the sample has been processed.

**16.** Add 500 µL of solution C5. Centrifuge for 30 sec at 10,000 x *g*

**17.** Discard the flow-through. Centrifuge for 1 min at 10,000 x *g*

**18.** Carefully place the MB Spin Column into a clean 2 mL Collection Tube. Avoiding splashing any Solution C5 onto the column

**19.** Add 100 µL of solution C6 to the center of the white filter membrane. Alternatively, you can use sterile DNase-free PCR-grade water for this step.

**20.** Centrifuge at RT for 30 sec at 10,000 x *g*. Discard the MB Spin Column. The DNA is now ready for downstream applications.

# References

1. Costea PI, Zeller G, Sunagawa S, Pelletier E, Alberti A, Levenez F, et al. Towards standards for human fecal sample processing in metagenomic studies. Nat Biotechnol. Nature Publishing Group; 2017;35:1069–76.

2. Qin J, Li R, Raes J, Arumugam M, Burgdorf KS, Manichanh C, et al. ARTICLES A human gut microbial gene catalogue established by metagenomic sequencing. 2010;464.
