# Supplementary material for: Assessment of fecal DNA extraction protocols for metagenomic studies
Source: Gigascience. 2020 Jul 13;9(7):giaa071. doi: 10.1093/gigascience/giaa071 (PMC7355182; doi:10.1093/gigascience/giaa071)
Supplement: giaa071_Supplemental_Files [file giaa071_supplemental_files.zip › Figure S2.pdf]

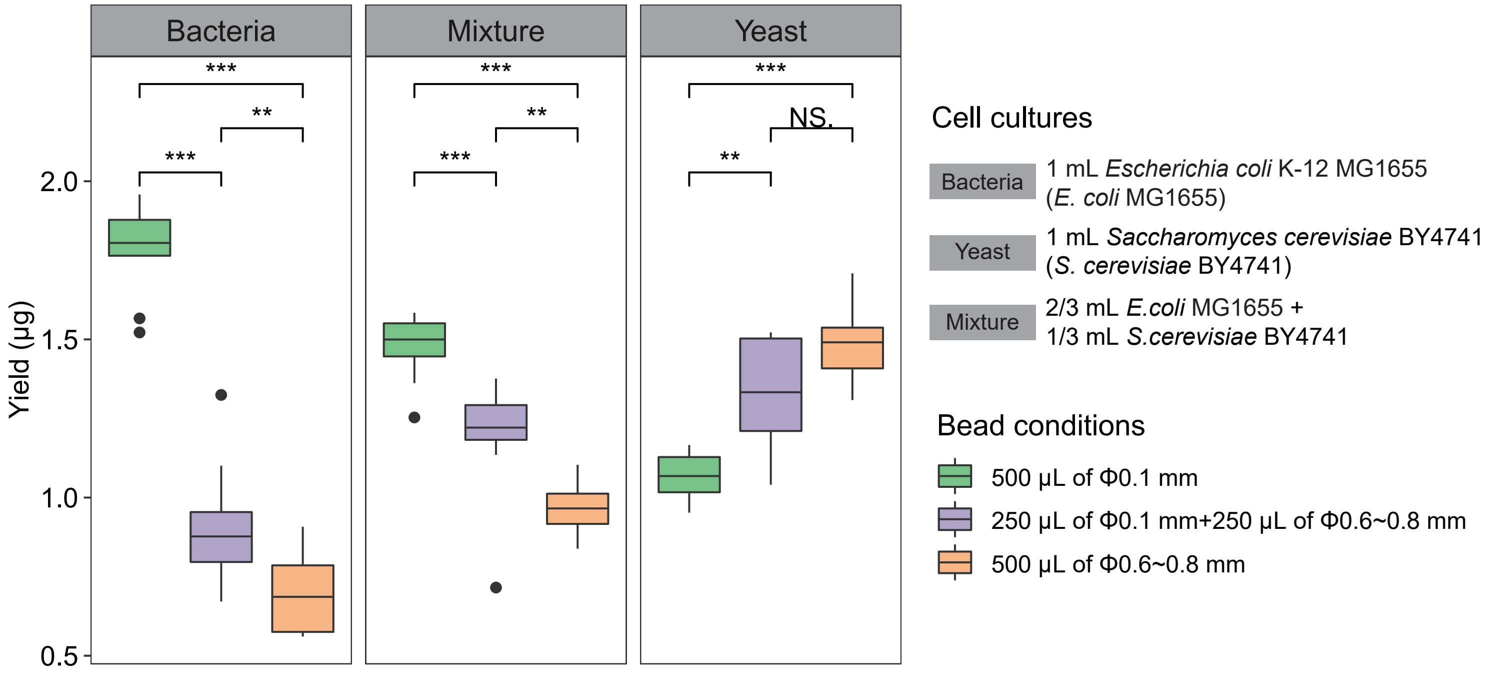

Bacteria
Mixture
Yeast
***
**
***
***
**
***
***
NS.
**
Yield (μg)
2.0
1.5
1.0
0.5
Cell cultures
Bacteria
1 mL *Escherichia coli* K-12 MG1655 (*E. coli* MG1655)
Yeast
1 mL *Saccharomyces cerevisiae* BY4741 (*S. cerevisiae* BY4741)
Mixture
2/3 mL *E.coli* MG1655 + 1/3 mL *S.cerevisiae* BY4741
Bead conditions
500 μL of Φ0.1 mm
250 μL of Φ0.1 mm+250 μL of Φ0.6~0.8 mm
500 μL of Φ0.6~0.8 mm
