# Supplementary material for: Assessment of fecal DNA extraction protocols for metagenomic studies
Source: Gigascience. 2020 Jul 13;9(7):giaa071. doi: 10.1093/gigascience/giaa071 (PMC7355182; doi:10.1093/gigascience/giaa071)
Supplement: giaa071_Supplemental_Files [file giaa071_supplemental_files.zip › Figure S5.pdf]

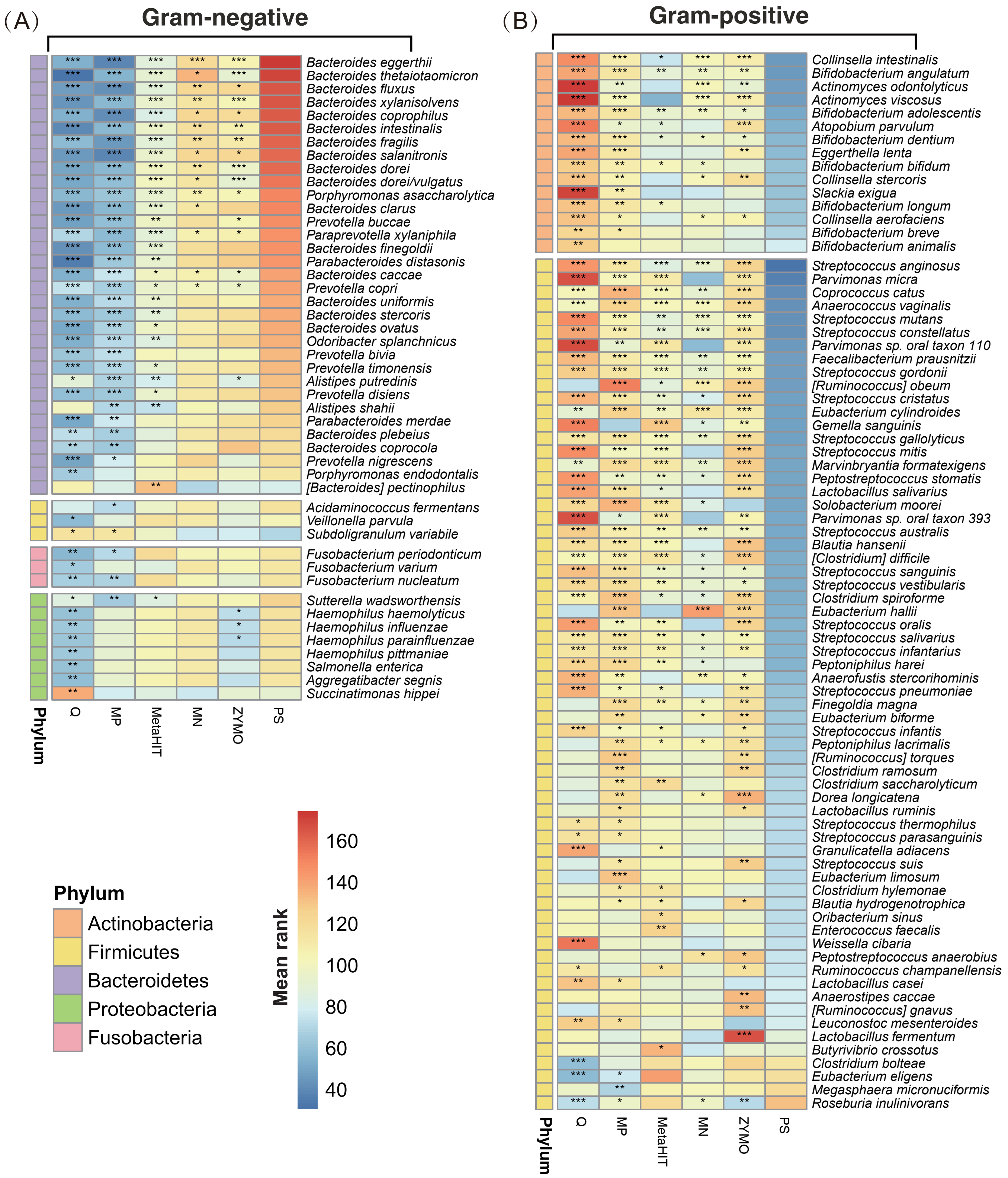
(A)
Gram-negative
Bacteroides eggerthii
Bacteroides thetaiotaomicron
Bacteroides fluxus
Bacteroides xylanisolvens
Bacteroides coprophilus
Bacteroides intestinalis
Bacteroides fragilis
Bacteroides salanitronis
Bacteroides dorei
Bacteroides dorei/vulgatus
Porphyromonas asaccharolytica
Bacteroides clarus
Prevotella buccae
Paraprevotella xylaniphila
Bacteroides finegoldii
Parabacteroides distasonis
Bacteroides caccae
Prevotella copri
Bacteroides uniformis
Bacteroides stercoris
Bacteroides ovatus
Odoribacter splanchnicus
Prevotella bivia
Prevotella timonensis
Alistipes putredinis
Prevotella disiens
Alistipes shahii
Parabacteroides merdae
Bacteroides plebeius
Bacteroides coprocola
Prevotella nigrescens
Porphyromonas endodontalis
[Bacteroides] pectinophilus
Acidaminococcus fermentans
Veillonella parvula
Subdoligranulum variabile
Fusobacterium periodonticum
Fusobacterium varium
Fusobacterium nucleatum
Sutterella wadsworthensis
Haemophilus haemolyticus
Haemophilus influenzae
Haemophilus parainfluenzae
Haemophilus pittmaniae
Salmonella enterica
Aggregatibacter segnis
Succinatimonas hippei
Phylum
Q
MP
MetaHIT
MN
ZYMO
PS
Phylum
Actinobacteria
Firmicutes
Bacteroidetes
Proteobacteria
Fusobacteria
Mean rank
160
140
120
100
80
60
40
(B)
Gram-positive
Collinsella intestinalis
Bifidobacterium angulatum
Actinomyces odontolyticus
Actinomyces viscosus
Bifidobacterium adolescentis
Atopobium parvulum
Bifidobacterium dentium
Eggerthella lenta
Bifidobacterium bifidum
Collinsella stercoris
Slackia exigua
Bifidobacterium longum
Collinsella aerofaciens
Bifidobacterium breve
Bifidobacterium animalis
Streptococcus anginosus
Parvimonas micra
Coprococcus catus
Anaerococcus vaginalis
Streptococcus mutans
Streptococcus constellatus
Parvimonas sp. oral taxon 110
Faecalibacterium prausnitzii
Streptococcus gordonii
[Ruminococcus] obeum
Streptococcus cristatus
Eubacterium cylindroides
Gemella sanguinis
Streptococcus gallolyticus
Streptococcus mitis
Marvinbryantia formatexigens
Peptostreptococcus stomatis
Lactobacillus salivarius
Solobacterium moorei
Parvimonas sp. oral taxon 393
Streptococcus australis
Blautia hansenii
[Clostridium] difficile
Streptococcus sanguinis
Streptococcus vestibularis
Clostridium spiroforme
Eubacterium hallii
Streptococcus oralis
Streptococcus salivarius
Streptococcus infantarius
Peptoniphilus harei
Anaerofustis stercorihominis
Streptococcus pneumoniae
Finegoldia magna
Eubacterium biforme
Streptococcus infantis
Peptoniphilus lacrimalis
[Ruminococcus] torques
Clostridium ramosum
Clostridium saccharolyticum
Dorea longicatena
Lactobacillus ruminis
Streptococcus thermophilus
Streptococcus parasanguinis
Granulicatella adiacens
Streptococcus suis
Eubacterium limosum
Clostridium hylemonae
Blautia hydrogenotrophica
Oribacterium sinus
Enterococcus faecalis
Weissella cibaria
Peptostreptococcus anaerobius
Ruminococcus champanellensis
Lactobacillus casei
Anaerostipes caccae
[Ruminococcus] gnavus
Leuconostoc mesenteroides
Lactobacillus fermentum
Butyrivibrio crossotus
Clostridium bolteae
Eubacterium eligens
Megasphaera micronuciformis
Roseburia inulinivorans
Phylum
Q
MP
MetaHIT
MN
ZYMO
PS
