# Supplementary figures and images for: Assessment of fecal DNA extraction protocols for metagenomic studies
Source: Gigascience. 2020 Jul 13;9(7):giaa071. doi: 10.1093/gigascience/giaa071 (PMC7355182; doi:10.1093/gigascience/giaa071)

(A)

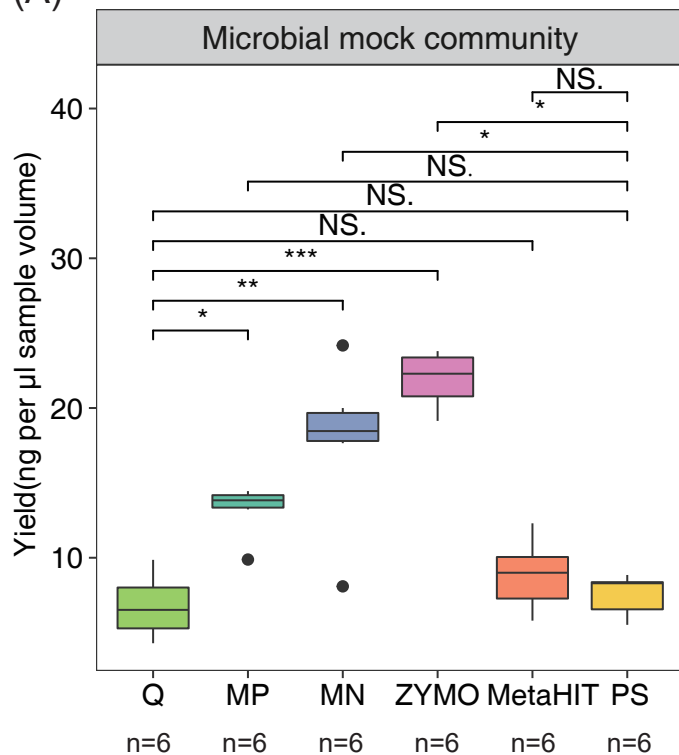

(B)

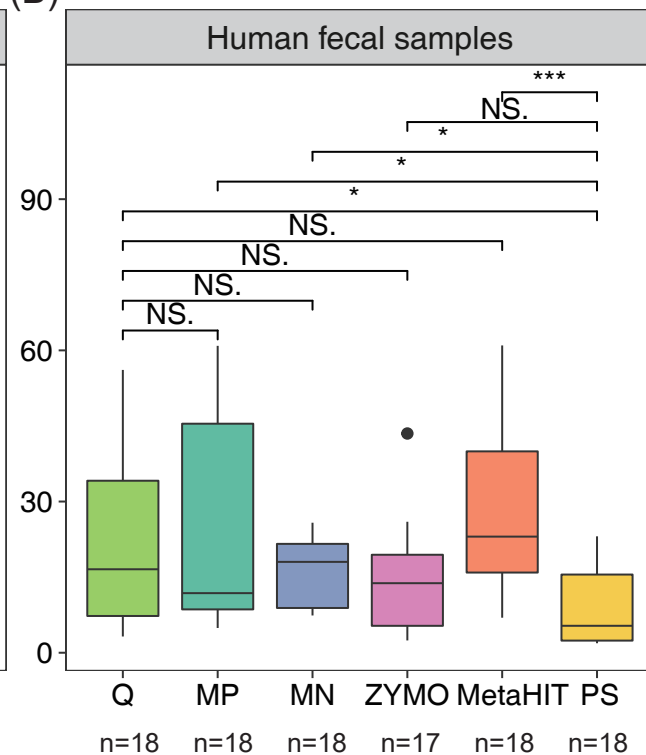

Supplement: giaa071_Supplemental_Files [file giaa071_supplemental_files.zip › Figure S1.pdf]

(A)

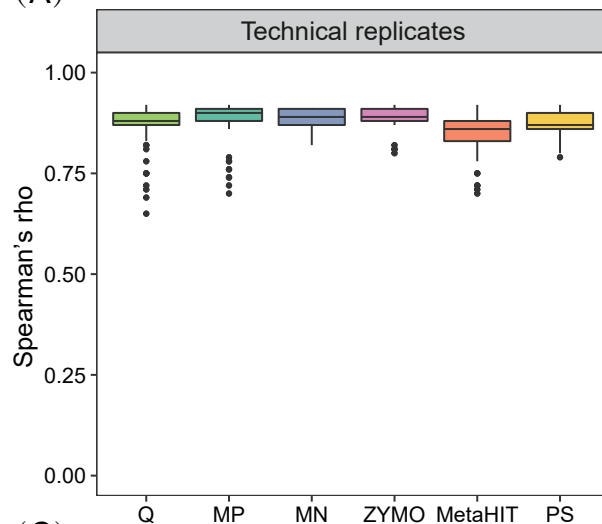

(B)

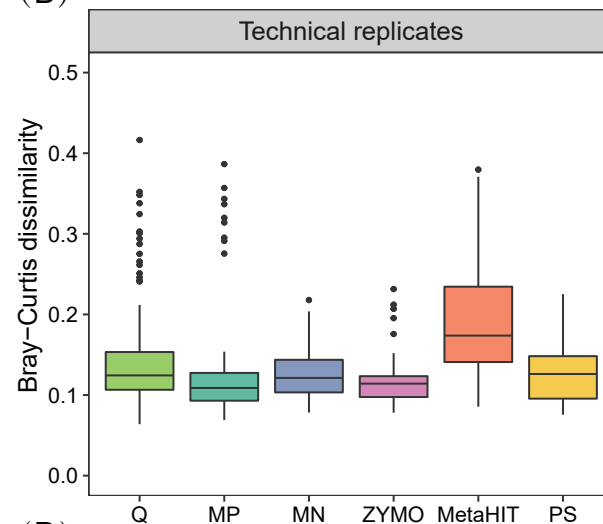

(C)

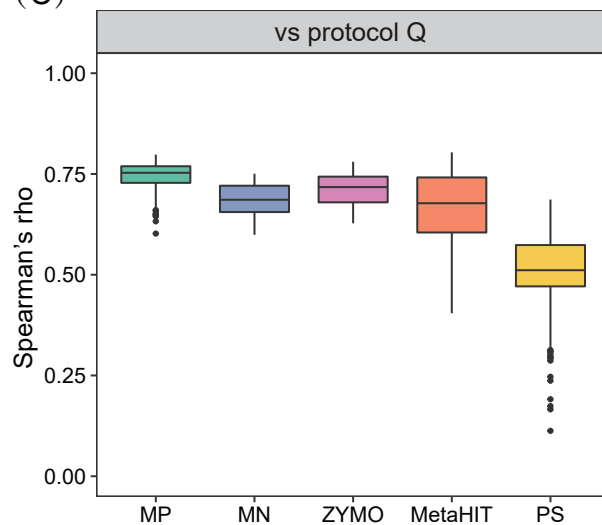

(D)

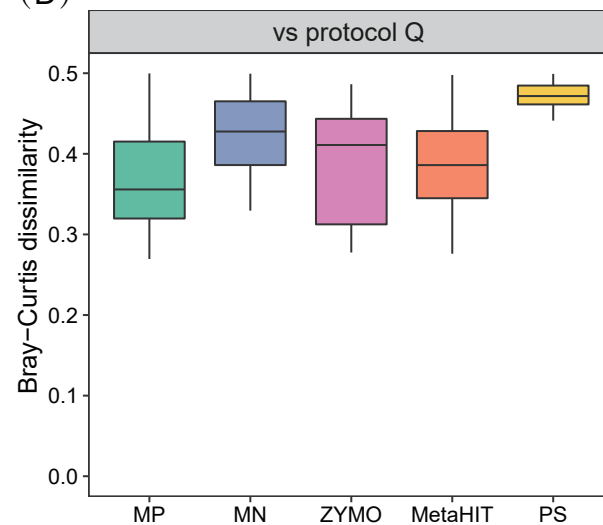

Supplement: giaa071_Supplemental_Files [file giaa071_supplemental_files.zip › Figure S3.pdf]

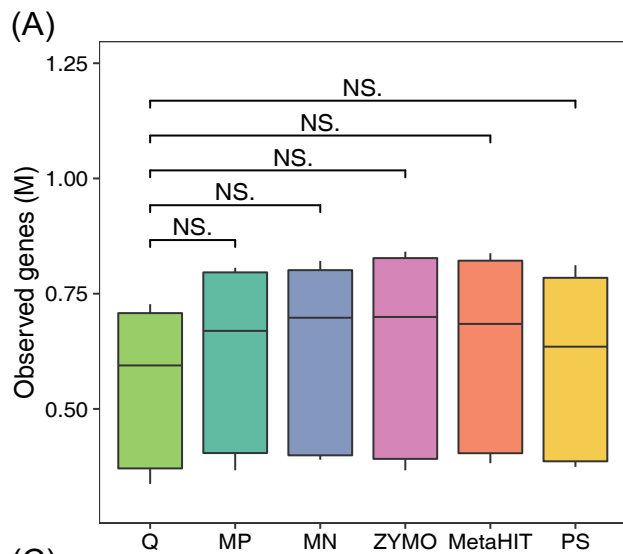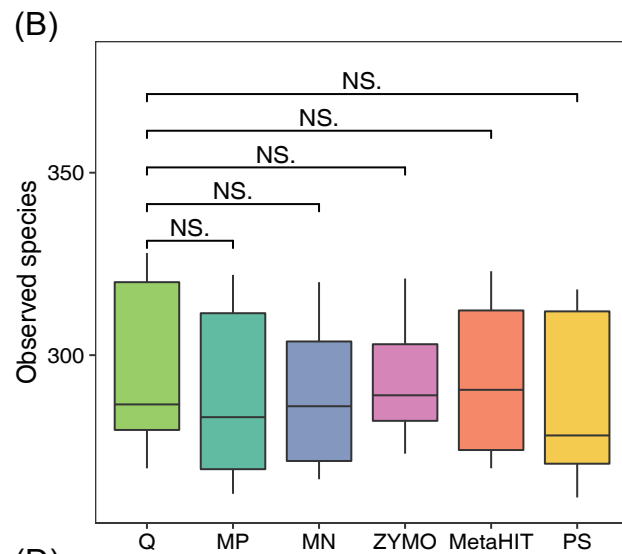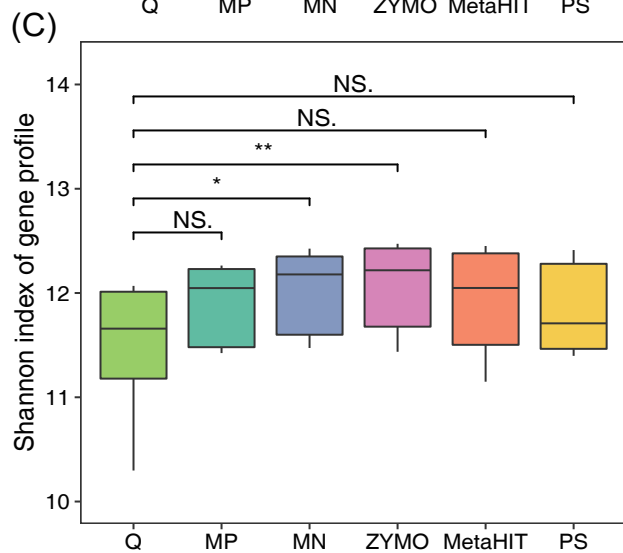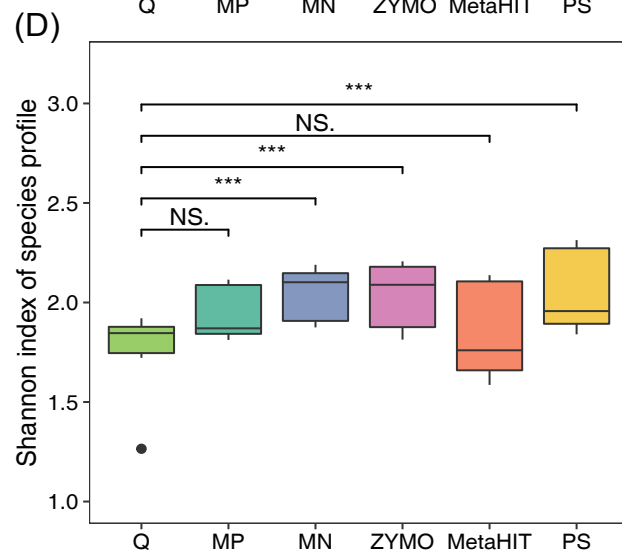

Supplement: giaa071_Supplemental_Files [file giaa071_supplemental_files.zip › Figure S4.pdf]

(A)

Gram-positive species

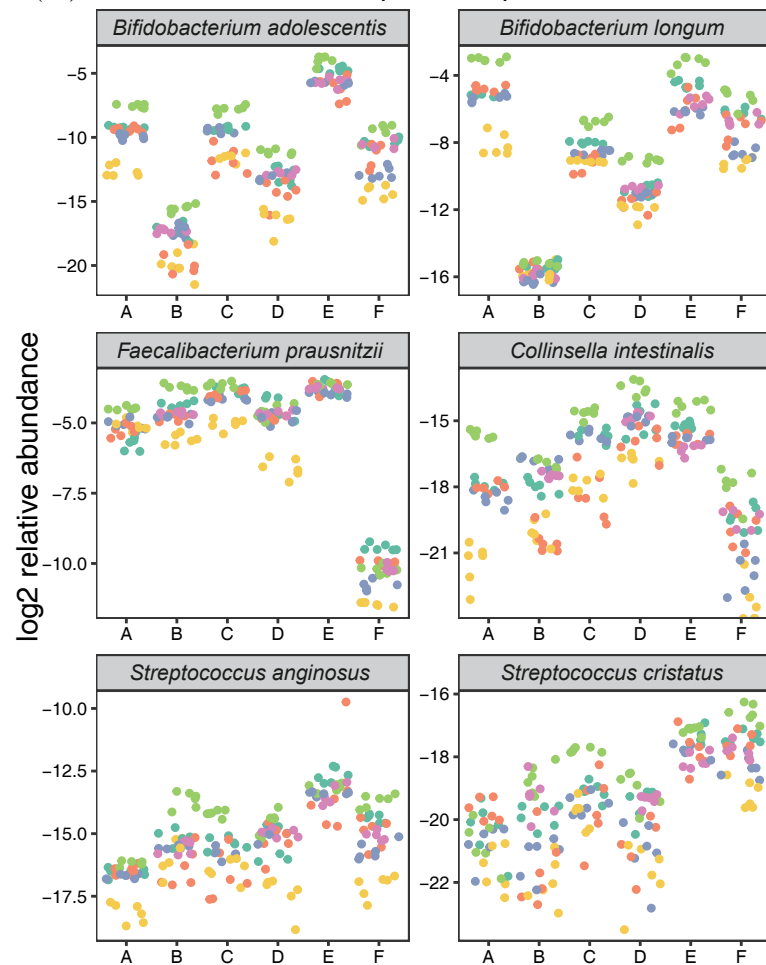

(B)

Gram-negative species

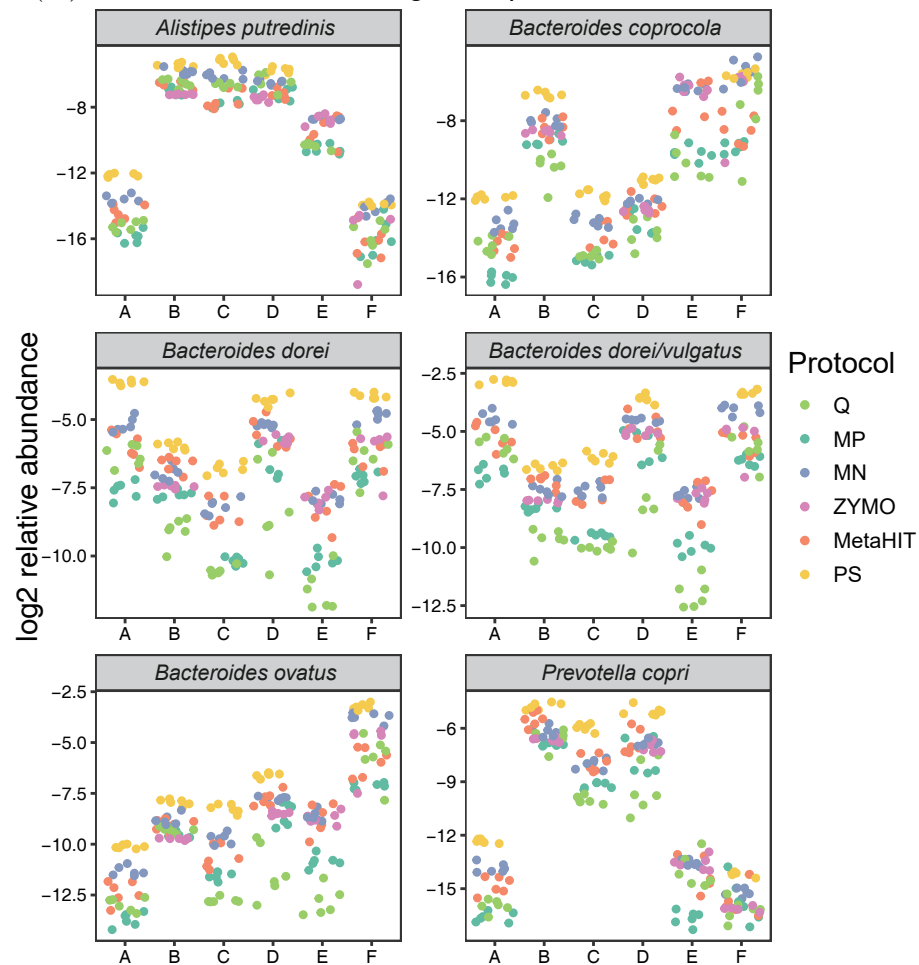

Supplement: giaa071_Supplemental_Files [file giaa071_supplemental_files.zip › Figure S6.pdf]

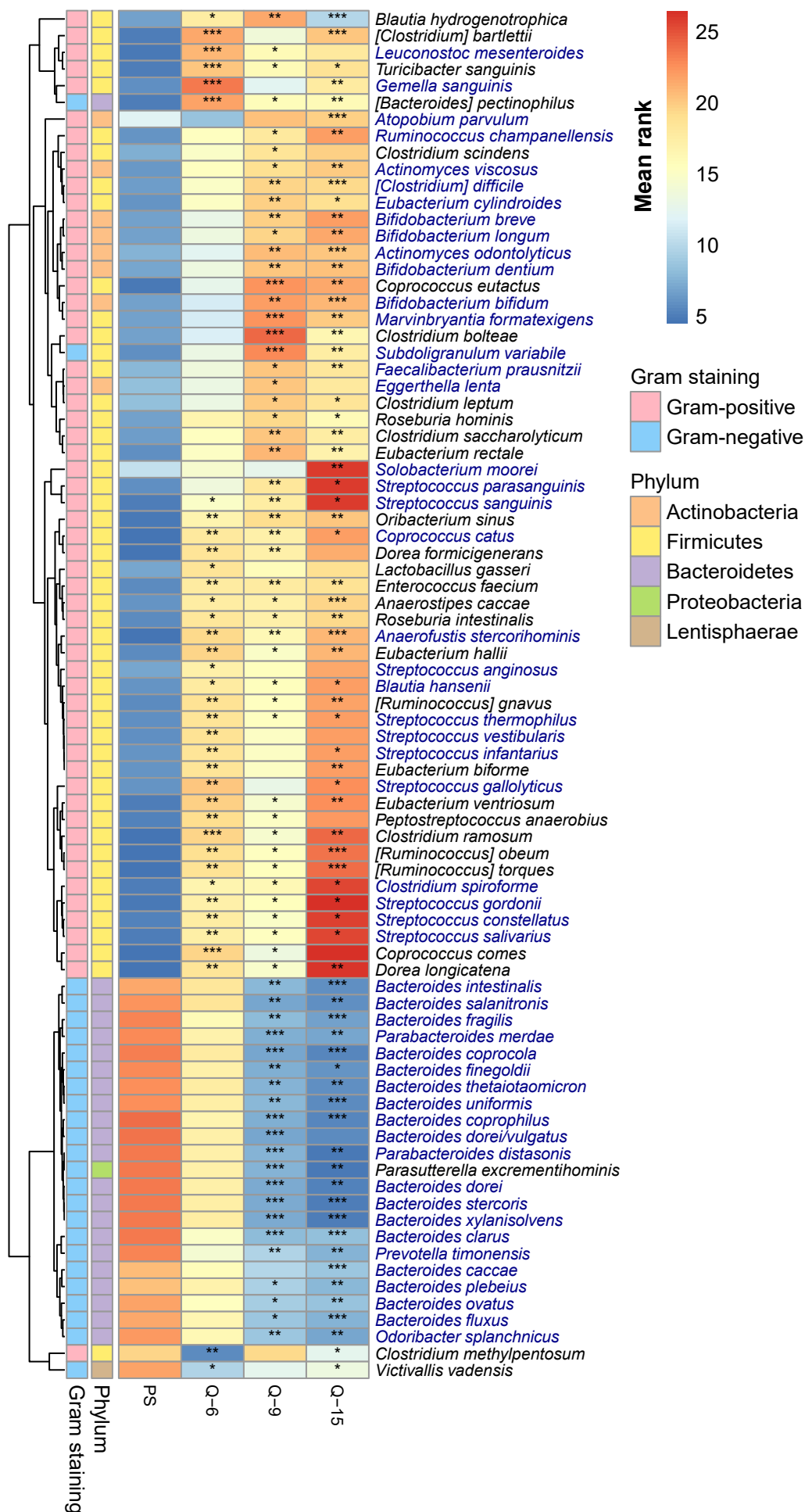

Supplement: giaa071_Supplemental_Files [file giaa071_supplemental_files.zip › Figure S7.pdf]
