# Supplementary material for: Assessment of fecal DNA extraction protocols for metagenomic studies
Source: Gigascience. 2020 Jul 13;9(7):giaa071. doi: 10.1093/gigascience/giaa071 (PMC7355182; doi:10.1093/gigascience/giaa071)
Supplement: giaa071_Response_to_Reviewer_Comments_Original_Submission [file giaa071_response_to_reviewer_comments_original_submission.pdf]

# Author's Response To Reviewer Comments

Close

We appreciate the reviewers' thorough advice and comments. By the changes made in the revised manuscript and the responses provided below, we hope that we have adequately addressed the reviewers' concerns.

Reviewer reports:
Reviewer #1:
In this study, six DNA extraction protocols were compared for whole genome shotgun metagenomic sequencing using one mock community and fecal samples. From the extraction of the mock community and individual cell cultures, larger beads were found to be associated with greater fungal extraction efficiency, but lower efficiency for bacteria. The MagPure kit had the highest mean accuracy in bacterial abundance from the mock community samples, but the five protocols other than the MetaHIT proposal tended to underestimate gram-positive bacteria and overestimate gram-negative bacteria. From the DNA extraction from human fecal samples, the PowerSoil kit appeared to have higher relative abundances of multiple gram-positive species and lower abundances of gram-negative species compared to all of the other protocols. However, individuals tended to cluster together regardless of DNA extraction method in a PCoA plot. The difference between PowerSoil extracted samples and the MetaHIT protocol were highlighted using previous data of health Chinese, Danish, and US adults where the US samples extracted using the PowerSoil had increased gram-negative and decreased gram-positive levels compared to the Chinese and Danish samples extracted using the MetaHIT protocol.

This is a valuable contribution to the literature as DNA extraction protocols need to be evaluated in order to establish recommended protocols for large-scale studies. Some specific comments on the manuscript are below.

Response:
We thank the reviewer for his/her positive comments on our manuscript. However, we need to clarify that one of our findings was that the use of the PowerSoil kit appeared to produce lower relative abundances of multiple gram-positive species and higher abundances of gram-negative species compared to all of the other protocols.

Major comments:

* Line 87: I commend the authors for including the full protocol details they used in this study. However, while looking through the listed protocols, it appears that automation was not used for any of the extraction protocols. One justification of this study was that standardized protocols are needed for large-scale population studies (line 65), but large-scale population studies would never use fully manual extractions. This should be mentioned as a limitation of the study.

Response: We thank reviewer 1 for this constructive comment.

We have modified the manuscript (in abstract and methods) to clearly describe that all six extraction protocols were manually processed by the same technician in the same lab.

The main aim of our study was to evaluate DNA extraction performance between five protocols and the recently proposed standardized protocol Q [1] for human fecal samples. As demonstrated by Costea et al., the consistent performance of Q (modified based on the commercial QIAamp® DNA Stool Mini Kit) in efficiently extracting DNA of gram-positive bacteria from human fecal samples was extensively assessed and proven by 21 worldwide laboratories. However, the proposed protocol Q has many processing steps (~156min /per extraction, Supplementary Table 1), and it can hardly be achieved automatically. On the other hand, except for the protocol Q and protocol MetaHIT, the rest four protocols we tested were all commercial kits and can be adopted in semi-automated DNA extraction systems.

In this study, we demonstrate that the MagPure kit showed highly consistent extraction performance for

human fecal samples comparable to protocol Q, but required fewer processing steps and less time (~45min /per extraction, Supplementary Table 1). We agree with the reviewer's comment that large-scale studies would never use fully manual extractions, and we believe that our results provide useful information for further developing and improving an automated, standardized fecal DNA extraction protocol/platform. We have also modified the discussion section in our manuscript to mention the limitation that the performance of the MagPure kit on an automatic extraction system was not evaluated in this study, and further efforts are required to assess the stability and consistency between manual and automated DNA extraction using this kit.

* Line 248: It would be helpful to discuss the limitations overall of this study. For example, these results may not extend to other sample types.

Response:
We thank reviewer 1 for this constructive comment.
We agree that our main conclusions concerning the performance of different DNA extraction protocols were drawn from human fecal samples, and these findings could not be directly generalized to other sample types (such as non-human environmental samples and those with high host DNA load and very low biomass) without further detailed studies on different sample types.
This limitation has been discussed in the revised version of the manuscript.

* Line 255: Were any blank samples included for each extraction method? Was sufficient DNA recovered for sequencing?

Response:
We did not include any blank samples for DNA extraction in this study.
We are aware that, for amplicon-based studies and extraction studies on low-biomass samples, blank samples (negative controls) are necessary to assess and trace the sources of possible nucleic acid contamination introduced from multiple experimental procedures.

For most samples, we did extract sufficient DNA from both the mock microbial community (average 0.77μg per sample) and real human fecal samples (average 4.31μg per sample) for shotgun metagenomic sequencing (see details of the DNA yield per sample in Supplementary Table 2). Also, all metagenomic datasets generated from DNA extracts of the mock microbial community had more than 97% of the total clean reads aligned to the ten reference genomes used in the mock community (SOAP 2.22, m=0, x=1000, r=1, l=30, M=4, S, p=6, v=5, S, c=0.95; see details of the reads mapping rate per sample in Supplementary Table 2), suggesting few contaminations were introduced during the extractions.

* Line 265: More details about the study participants and sample collection would be helpful. For example, what proportion were women? What was the age range of adults? How was the fecal sample collected? How long did it take for the samples to be transported back to the laboratory?

Response: We apologize for the omissions in the Methods section.
We have added detailed information (sex and age) of the six participants in the revised Supplementary Table S2.
We have also modified the methods (in line 291-300) to clearly describe the process of collection, transport and storage of fecal samples before DNA extraction: "Six healthy volunteers including one four-year-old child and five adults (32 ± 3 years old) were recruited from BGI Europe employees or family members, Copenhagen, Denmark (See detailed information in Supplementary Table 2). All volunteers or the guardian consented to provide fecal samples for this study. About 10-15 grams of stool was freshly collected per participant at home by using a 50mL sterile conical tube, and copies of printed instructions were used to guide the adult volunteers or the child's legal guardian for self-collection of fecal samples. After collection, samples were stored at -20 °C and transported to the laboratory on the second day with ice packs in forty minutes. Then, each sample was diluted with 1~1.5 volumes (15 mL) of Tris-EDTA (TE, 10 mM Tris pH 8.0 and 1 mM EDTA,Thermo Fisher Scientific) buffer, homogenized and divided into 36 aliquots (500 μL per aliquot). All stool aliquots were stored at -80 °C before DNA extraction."

* Line 278: It would be important in the results to describe the failures - it looks like all samples from specific individuals failed for the PowerSoil and Zymo extractions. If you restrict to only individuals present in all extraction methods, are your results consistent?

Response:
We thank the reviewer for this valuable suggestion.
Yes, we are aware of the extraction failure in specific individuals, as also stated in the Methods section (in line 310-313) : “Six fecal samples extracted using protocol PS (individual E) and 13 fecal samples extracted using protocol ZYMO (six of individual A, six of individual C, and one of individual F) that yielded less than 500ng and failed for library preparation, were removed from further processing.”

As all extractions on the human fecal samples by a given protocol were performed in parallel at the same time, the failure is unlikely to be caused by any laboratory procedures. However, we did not have enough fecal samples for a second-round extraction experiment on these failed samples (as a total of 36 samples per individual were used to generate six technical replicates for six different methods). On the other hand, we have successfully constructed sequencing libraries and sequenced DNA from all 36 extractions of the mock microbial community, although they had a lower microbial DNA yield as compared to the human fecal samples.

One explanation for the failure of samples from specific individuals could be that certain extraction kits (PowerSoil and Zymo) might not effectively remove complex compounds (such as humic acids, polysaccharides, bile acids and lipids, which were not contained in the mock microbial community) in fecal samples which possibly might act as PCR inhibitors to impact sequencing library construction[2].

As we show in Figure S6 (Relative abundance distributions of representative gut bacterial species at the individual level), the PowerSoil kit yielded obviously higher relative abundances of six selected representative gram-negative species (yellow) in all five individuals than the other five extraction protocols. Consistently, the MP and protocol Q had similar low relative abundances of all gram-negative species (light green and green) in all human fecal samples. These observations at the individual level were in line with the findings based on all samples (Figure 4).

To address the reviewer's concerns, we here also present the intra-individual relative abundances of 40 species in DNA samples extracted by all protocols.
In each individual, PS-extracted samples (yellow) showed consistently lower relative abundances of most gram-positive species (upper panel, Rebuttal Figure 1a) and higher relative abundances of most gram-negative species (lower panel, Rebuttal Figure 1b) than those extracted using the other protocols. Also, samples extracted by Q and MP showed consistently lower relative abundances of most gram-negative species than PS and other protocols (Rebuttal Figure 1b). These observations were consistent in different individuals and were in line with our main reported findings (Figure 4).

Rebuttal Figure 1 Relative abundance distributions of representative gut bacterial species within each individual.
(a), gram-positive species, (b), gram-negative species. Each line indicates the relative species abundance in the DNA sample extracted using a given extraction protocol. Light green, protocol Q; green, protocol MP; blue, protocol MN; purple, protocol ZYMO; orange, protocol MetaHIT; yellow, protocol PS. X-axis indicates log2 transformed relative abundance of a given species, only top 20 abundant gram-positive (a) or gram-negative (b) species are shown (Y axis). Each panel indicates an individual (A to F).

Minor comments:
* Line 57: Shotgun metagenomics also has its own limitations so you cannot completely ignore 16S rRNA gene sequencing.

Response: We thank reviewer 1 for this constructive suggestion.
In the revised manuscript, we have stated: “During the past two decades, PCR-based amplicon sequencing, a flexible and cost-effective method to determine microbial composition, has greatly improved our understanding of human microbiome. However, considering the known effects of PCR conditions on amplification biases such as primers, specific hypervariable regions, and annealing temperature[3,4], amplicon sequencing is insufficient for accurately evaluating quantitative performance of bacterial DNA extraction protocols.”

* Line 245: Mock communities in a matrix similar to a fecal sample would be ideal since the artificial communities do not reflect potential inhibitors and other materials found in a fecal sample.

Response:

We fully agree with the reviewer that a mock community in a matrix similar to a human fecal sample would be an ideal material to evaluate the performance of fecal DNA extraction protocols. However, due to the difficulty of culturing various kinds of anaerobic gut microbes in the laboratory, there is still no available standardized, commercial mock microbial community related to human feces. Also, as we discussed above, a mixture of microbial communities could hardly mimic the complex mixture of compounds in real fecal samples, which might inhibit the activities of enzymes for PCR-based library construction.

In the revised manuscript, we have extended the limitations of the microbial mock community in line 262-266: "In addition, the mock communities from both studies were both composed of human pathogenic bacteria or bacteria isolated from a non-human environment, which do not reflect the human gut microbial composition. Furthermore, such simple mixtures of bacteria and fungi do not contain other compounds in feces such as humic acids, polysaccharides, bile acids and lipids, which might potentially inhibit the activity of enzymes used for subsequent PCR-based library construction and sequencing[2]."

Reviewer #2:
The manuscript titled "Assessment of fecal DNA extraction protocols for metagenomics studies" by Yang et al. describe the higher efficacy of MP method for the fecal DNA extraction procedure. This study also compares validity and reproducibility of six different DNA extraction method with mock and human fecal samples. As mentioned by the authors, standardized and robust DNA extraction protocol is still needed for the comparison between globally produced gut microbiome data. In addition, I agree with the necessity of new analytical methods for comprehensive and accurate understand of gut mycobiome as well. In that respect, I think your manuscript is timely necessary and important. However, there are some point might be considered in revision.

The most important point is that the microorganisms contained in currently used mock community are not abundant members of human gut microbiome. In the recent study by Sunagawa et al., as referred in your manuscript, the authors construct a mock community considering human gut microbial composition. While MP showed higher mean accuracy in bacterial abundance estimation than other protocol and Q showed lowest recovery of the two yeast genomes in mock sample analysis. Microbiome extracted with Q protocol still have distinct community composition compared to the other method, even with MP protocol, especially in G+ bacteria. So, I recommend the authors check where this discrepancy come from with the other mock samples or manually constructed human microbiome mock samples.

Response: We thank reviewer 2 for this constructive comment.

In the current study, we used a commercial mock community (ZymoBIOMICS Microbial Community Standard, Catalog No. D6300) containing cells of eight bacteria (each making up 12%) and two yeast strains (each contributing 2%). All these species are human pathogens or isolated from a non-human environment, facultative anaerobes (easy to be cultured), and are not high-abundant residents in the human gut.

Similarly, the benchmark study (Sunagawa et al, metioned by the reviewer should be Costea et al, 10.1038/nbt.3960) also used a mock community containing 10 bacterial species that were generally absent from the healthy gut microbiota, including Prevotella melaninogenica (G-), Clostridium perfringens (G+), Salmonella enterica (G-, also used in the current study), Clostridium difficile (G+), Lactobacillus plantarum (G+), Clostridium saccharolyticum (G+), Yersinia pseudotuberculosis (G-), Vibrio cholerae (G-), Blautia hansenii (G+) and Fusobacterium nucleatum (G-)(Costea et al, 10.1038/nbt.3960, Figure 6). Thus, both studies did not use representative and high-abundant gut microbes for the mock materials.

(Figure 6, Costea et al, 10.1038/nbt.3960)

We are aware that the assessment of mock microbial community might not fully reflect the extraction performance in real human fecal samples. Also, we did observe the inconsistency of the extraction efficiency of gram-positive bacteria between the mock community and fecal samples. Except for the MetaHIT protocol, all other five protocols underestimated the abundance of four among the five gram-positive bacteria in the mock (Staphylococcus aureus, Enterococcus faecalis, Listeria monocytogenes and Bacillus subtilis) but overestimated the abundance of gram-positive Lactobacillus fermentum (Figure 2). By contrast, four protocols (MN, ZYMP, Q and PS) overestimated the abundance of all three gram-

negative bacteria (Salmonella enterica, Escherichia coli and Pseudomonas aeruginosa) (Figure 2). As reported by Costea et al (10.1038/nbt.3960, Figure 6), regardless of extracting DNA from the mock itself or from fecal sample with a spike-in mock community, protocol Q (blue) underestimated the abundance of gram-positive bacteria including C. perfringens, C. difficile and L. plantarum and overestimated the abundance of three gram-negative members including P. melaninogenica, S. enterica and F. nucleatum. My suggestion: regardless of whether DNA was extracted from a mock community or from a fecal sample with a spike-in mock community, protocol Q underestimated the abundances of gram-positive bacteria including Clostridium perfringens, C. difficile and Lactobacillus plantarum and overestimated the abundance of three gram-negative members including S. enterica, Prevotella melaninogenica and Fusobacterium nucleatum. Thus, the observations based on mock communities in the two studies were somehow consistent, suggesting overall different efficiencies of obtaining whole-genome DNA from gram-positive and gram-negative bacteria, as well as variable efficiencies between different gram-positive species.
We note the discrepancy between the DNA extraction performance on mock communities and fecal samples in our study as well as the benchmark study (Costea et al). Both studies have demonstrated that the fecal DNA samples extracted by protocol Q displayed higher relative abundances of multiple gram-positive species than other methods.

As we note in our response to the Reviewer 1, so far, it is still challenging to create a mock microbial community that can mimic human feces. The two reasons are that 1) most of the gut residents are anaerobic and hard to culture and 2) a simple mixture of microbial species will not reflect the complex, highly variable chemical and physical properties of human feces, which might potentially impact the activities of enzymes for downstream library construction and sequencing. Thus, extraction performance based on an MMC will not precisely and unbiasedly reflect extraction performance on human fecal samples.

Also, for both studies, quantitative performance on extracting human gut microbiome between protocols has been interpreted based on bacterial relative abundance but not absolute abundance, which we measured in the mock. Further efforts are still needed to quantify absolute microbial abundances in fecal mock materials with a mixture of both abundant gut microbes and non-living fecal compounds, and in real fecal samples to accurately assess the quantification biases of different protocols.

We have extensively revised our manuscript to discuss the limitations of our study as well as previous ones (in line 251-270), and we hope we have addressed the reviewer's concerns about the discrepancy of the DNA extraction performance on mock communities and fecal samples.

2. As described by the authors, very low levels of mycobiome only in few fecal samples were detected with tested protocols and fungal sequence reads were only identified in one sample both MP and Q protocols. Therefore, I am not sure we can determine that MP is a more effective method for human metagenomic analysis than Q protocol, even though MP showed greater efficacy in mock sample analysis than Q protocol.

Response:
First, a measurable fungal abundance was only detected in few samples in this study. However, these observations do not necessarily imply that there were no fungal genomes extracted in human feces by the six protocols.
Previous studies have demonstrated very low levels of fungi in human fecal samples[5–7]. As reported by Richard et al, the number of fungi in faces has been shown to be far lower than that of bacteria, with 105 to 106 fungal cells per gram of fecal matter compared with 1011 bacterial cells per gram [7]. In addition, the genome sizes of fungi are also much greater than that of bacteria. Thus, a much greater amount of sequencing data than we generated in the current study is needed to evaluate the performance of fecal mycobiome extraction across protocols. Amplicon-based approaches (18S rRNA-based or ITS-based) seem still to be more cost-effective and appropriate in order to assess the mycobiome in human fecal samples, and such amplicon-based approaches have been successfully applied in several studies[8–10].

Second, our study has shown a positive correlation between the bead size and extraction efficiency of yeast DNA (Figure 2d,f and Supplementary Figure 2), and the two protocols (MN using 0.6-0.8mm beads, and ZYMO using a mixture of 0.1&0.5 beads) have shown better performance in recovering genome coverage and abundance of the two yeast strains than other three protocols using 0.1mm beads (MetaHIT, MP and Q). We concur that a limitation of our study is that we did not apply amplicon-based sequencing to assess whether the performance of extracting fungi DNA using the different protocols is

the same using mock or fecal samples.

We suggest researchers to consider using protocols with larger beads when their research interests are mainly focused on the mycobiome. Due to the limited sequencing depth of mycobiome from our shotgun metagenomic datasets, we were not able to compare the extracting performance of mycobiome between the MP and Q, or between any two protocols. We have modified our results (in line 188-189 and discussions (in line 243-250) to clearly describe our findings to help the reviewers and readers better understand our results.

References

1. Costea PI, Zeller G, Sunagawa S, Pelletier E, Alberti A, Levenez F, et al. Towards standards for human fecal sample processing in metagenomic studies. Nature Biotechnology [Internet]. Nature Publishing Group; 2017;35:1069–76. Available from: http://dx.doi.org/10.1038/nbt.3960
2. Schrader C, Schielke A, Ellerbroek L, Johne R. PCR inhibitors - occurrence, properties and removal. Journal of Applied Microbiology. 2012;113:1014–26.
3. Orpana AK, Ho TH, Stenman J. Multiple heat pulses during PCR extension enabling amplification of GC-rich sequences and reducing amplification bias. Analytical Chemistry. 2012;
4. Laursen MF, Dalgaard MD, Bahl MI. Genomic GC-content affects the accuracy of 16S rRNA gene sequencing bsed microbial profiling due to PCR bias. Frontiers in Microbiology. 2017;
5. Huffnagle GB, Noverr MC. The emerging world of the fungal microbiome. Trends in Microbiology. 2013.
6. Sam QH, Chang MW, Chai LYA. The fungal mycobiome and its interaction with gut bacteria in the host. International Journal of Molecular Sciences. 2017.
7. Richard ML, Sokol H. The gut mycobiota: insights into analysis, environmental interactions and role in gastrointestinal diseases. Nature Reviews Gastroenterology and Hepatology [Internet]. Springer US; 2019;16:331–45. Available from: http://dx.doi.org/10.1038/s41575-019-0121-2
8. Frau A, Kenny JG, Lenzi L, Campbell BJ, Ijaz UZ, Duckworth CA, et al. DNA extraction and amplicon production strategies deeply influence the outcome of gut mycobiome studies. Scientific Reports [Internet]. Springer US; 2019;9:1–17. Available from: http://dx.doi.org/10.1038/s41598-019-44974-x
9. Nash AK, Auchtung TA, Wong MC, Smith DP, Gesell JR, Ross MC, et al. The gut mycobiome of the Human Microbiome Project healthy cohort. Microbiome. 2017;
10. Zuo T, Wong SH, Cheung CP, Lam K, Lui R, Cheung K, et al. Gut fungal dysbiosis correlates with reduced efficacy of fecal microbiota transplantation in Clostridium difficile infection. Nature Communications. 2018;

Close
