# Supplementary material for: Assessment of fecal DNA extraction protocols for metagenomic studies
Source: Gigascience. 2020 Jul 13;9(7):giaa071. doi: 10.1093/gigascience/giaa071 (PMC7355182; doi:10.1093/gigascience/giaa071)
Supplement: giaa071_Reviewer_1_Report_Original_Submission — Emily Vogtmann -- 2/19/2020 Reviewed [file giaa071_reviewer_1_report_original_submission.pdf]

Reviewer Report

Title: Assessment of fecal DNA extraction protocols for metagenomic studies

Version: Original Submission **Date:** 2/19/2020

Reviewer name: Emily Vogtmann

Reviewer Comments to Author:

In this study, six DNA extraction protocols were compared for whole genome shotgun metagenomic sequencing using one mock community and fecal samples. From the extraction of the mock community and individual cell cultures, larger beads were found to be associated with greater fungal extraction efficiency, but lower efficiency for bacteria. The MagPure kit had the highest mean accuracy in bacterial abundance from the mock community samples, but the five protocols other than the MetaHIT proposal tended to underestimate gram-positive bacteria and overestimate gram-negative bacteria. From the DNA extraction from human fecal samples, the PowerSoil kit appeared to have higher relative abundances of multiple gram-positive species and lower abundances of gram-negative species compared to all of the other protocols. However, individuals tended to cluster together regardless of DNA extraction method in a PCoA plot. The difference between PowerSoil extracted samples and the MetaHIT protocol were highlighted using previous data of health Chinese, Danish, and US adults where the US samples extracted using the PowerSoil had increased gram-negative and decreased gram-positive levels compared to the Chinese and Danish samples extracted using the MetaHIT protocol. This is a valuable contribution to the literature as DNA extraction protocols need to be evaluated in order to establish recommended protocols for large-scale studies. Some specific comments on the manuscript are below.

Major comments:

* Line 87: I commend the authors for including the full protocol details they used in this study. However, while looking through the listed protocols, it appears that automation was not used for any of the extraction protocols. One justification of this study was that standardized protocols are needed for large-scale population studies (line 65), but large-scale population studies would never use fully manual extractions. This should be mentioned as a limitation of the study.
* Line 248: It would be helpful to discuss the limitations overall of this study. For example, these results may not extend to other sample types.
* Line 255: Were any blank samples included for each extraction method? Was sufficient DNA recovered for sequencing?
* Line 265: More details about the study participants and sample collection would be helpful. For example, what proportion were women? What was the age range of adults? How was the fecal sample collected? How long did it take for the samples to be transported back to the laboratory?
* Line 278: It would be important in the results to describe the failures - it looks like all samples from specific individuals failed for the PowerSoil and Zymo extractions. If you restrict to only individuals present in all extraction methods, are your results consistent?

Minor comments:

* Line 57: Shotgun metagenomics also has its own limitations so you cannot completely ignore 16S rRNA gene sequencing.
* Line 245: Mock communities in a matrix similar to a fecal sample would be ideal since the artificial communities do not reflect potential inhibitors and other materials found in a fecal sample.

Methods

Are the methods appropriate to the aims of the study, are they well described, and are necessary controls included? Choose an item.

Conclusions

Are the conclusions adequately supported by the data shown? Choose an item.

Reporting Standards

Does the manuscript adhere to the journal's guidelines on [minimum standards of reporting?]() Choose an item.

Choose an item.

Statistics

Are you able to assess all statistics in the manuscript, including the appropriateness of statistical tests used? Choose an item.

Quality of Written English

Please indicate the quality of language in the manuscript: Choose an item.

Declaration of Competing Interests

Please complete a declaration of competing interests, considering the following questions:

- Have you in the past five years received reimbursements, fees, funding, or salary from an organisation that may in any way gain or lose financially from the publication of this manuscript, either now or in the future?
- Do you hold any stocks or shares in an organisation that may in any way gain or lose financially from the publication of this manuscript, either now or in the future?
- Do you hold or are you currently applying for any patents relating to the content of the manuscript?
- Have you received reimbursements, fees, funding, or salary from an organization that holds or has applied for patents relating to the content of the manuscript?
- Do you have any other financial competing interests?
- Do you have any non-financial competing interests in relation to this paper?

If you can answer no to all of the above, write 'I declare that I have no competing interests' below. If your reply is yes to any, please give details below.

I declare that I have no competing interests.

I agree to the open peer review policy of the journal. I understand that my name will be included on my report to the authors and, if the manuscript is accepted for publication, my named report including any attachments I upload will be posted on the website along with the authors' responses. I agree for my report to be made available under an Open Access Creative Commons CC-BY license (http://creativecommons.org/licenses/by/4.0/). I understand that any comments which I do not wish to be included in my named report can be included as confidential comments to the editors, which will not be published.

Choose an item.

To further support our reviewers, we have joined with Publons, where you can gain additional credit to further highlight your hard work (see: https://publons.com/journal/530/gigascience). On publication of this paper, your review will be automatically added to Publons, you can then choose whether or not to claim your Publons credit. I understand this statement.

Yes Choose an item.
