# Supplementary material for: Assessment of fecal DNA extraction protocols for metagenomic studies
Source: Gigascience. 2020 Jul 13;9(7):giaa071. doi: 10.1093/gigascience/giaa071 (PMC7355182; doi:10.1093/gigascience/giaa071)
Supplement: giaa071_Reviewer_2_Report_Original_Submission — Young-Do Nam -- 2/27/2020 Reviewed [file giaa071_reviewer_2_report_original_submission.pdf]

Reviewer Report

Title: Assessment of fecal DNA extraction protocols for metagenomic studies

Version: Original Submission **Date:** 2/27/2020

Reviewer name: Young-Do Nam

Reviewer Comments to Author:

The manuscript titled "Assessment of fecal DNA extraction protocols for metagenomics studies" by Yang et al. describe the higher efficacy of MP method for the fecal DNA extraction procedure. This study also compares validity and reproducibility of six different DNA extraction method with mock and human fecal samples. As mentioned by the authors, standardized and robust DNA extraction protocol is still needed for the comparison between globally produced gut microbiome data. In addition, I agree with the necessity of new analytical methods for comprehensive and accurate understand of gut mycobiome as well. In that respect, I think your manuscript is timely necessary and important. However, there are some point might be considered in revision.

1. The most important point is that the microorganisms contained in currently used mock community are not abundant members of human gut microbiome. In the recent study by Sunagawa et al., as referred in your manuscript, the authors construct a mock community considering human gut microbial composition. While MP showed higher mean accuracy in bacterial abundance estimation than other protocol and Q showed lowest recovery of the two yeast genomes in mock sample analysis, microbiome extracted with Q protocol still have distinct community composition compared to the other method, even with MP protocol, especially in G+ bacteria. So, I recommend the authors check where this discrepancy come from with the other mock samples or manually constructed human microbiome mock samples.
2. As described by the authors, very low levels of mycobiome only in few fecal samples were detected with tested protocols and fungal sequence reads were only identified in one sample both MP and Q protocols. Therefore, I am not sure we can determine that MP is a more effective method for human metagenomic analysis than Q protocol, even though MP showed greater efficacy in mock sample analysis than Q protocol.

Methods

Are the methods appropriate to the aims of the study, are they well described, and are necessary controls included? Choose an item.

Conclusions

Are the conclusions adequately supported by the data shown? Choose an item.

Reporting Standards

Does the manuscript adhere to the journal's guidelines on [minimum standards of reporting?] Choose an item.

Choose an item.

Statistics

Are you able to assess all statistics in the manuscript, including the appropriateness of statistical tests used? Choose an item.

Quality of Written English

Please indicate the quality of language in the manuscript: Choose an item.

Declaration of Competing Interests

Please complete a declaration of competing interests, considering the following questions:

- Have you in the past five years received reimbursements, fees, funding, or salary from an organisation that may in any way gain or lose financially from the publication of this manuscript, either now or in the future?
- Do you hold any stocks or shares in an organisation that may in any way gain or lose financially from the publication of this manuscript, either now or in the future?
- Do you hold or are you currently applying for any patents relating to the content of the manuscript?
- Have you received reimbursements, fees, funding, or salary from an organization that holds or has applied for patents relating to the content of the manuscript?
- Do you have any other financial competing interests?
- Do you have any non-financial competing interests in relation to this paper?

If you can answer no to all of the above, write 'I declare that I have no competing interests' below. If your reply is yes to any, please give details below.

I declare that I have no competing interests

I agree to the open peer review policy of the journal. I understand that my name will be included on my report to the authors and, if the manuscript is accepted for publication, my named report including any attachments I upload will be posted on the website along with the authors' responses. I agree for my report to be made available under an Open Access Creative Commons CC-BY license (http://creativecommons.org/licenses/by/4.0/). I understand that any comments which I do not wish to be included in my named report can be included as confidential comments to the editors, which will not be published.

Choose an item.

To further support our reviewers, we have joined with Publons, where you can gain additional credit to further highlight your hard work (see: https://publons.com/journal/530/gigascience). On publication of this paper, your review will be automatically added to Publons, you can then choose whether or not to claim your Publons credit. I understand this statement.

Yes Choose an item.
